# Robust Asymmetric Localization of Planar Polarity Proteins Is Associated with Organization into Signalosome-like Domains of Variable Stoichiometry

**DOI:** 10.1016/j.celrep.2016.11.021

**Published:** 2016-12-06

**Authors:** Helen Strutt, Jessica Gamage, David Strutt

**Affiliations:** 1 Bateson Centre, Department of Biomedical Science, University of Sheffield, Western Bank, Sheffield S10 2TN, UK

**Keywords:** signaling, polarity, planar polarity, PCP, frizzled, stoichiometry

## Abstract

In developing epithelia, the core planar polarity proteins physically interact with each other and localize asymmetrically at opposite cell ends, forming intercellular complexes that link the polarity of neighboring cells. Using quantitative imaging to examine the composition of the core protein complex in vivo, we find that complex composition is unexpectedly plastic. The transmembrane proteins Frizzled and Flamingo form a stoichiometric nucleus in the complex, while the relative levels of the other four core proteins can vary independently. Exploring the functional consequences of this, we show that robust cell polarization is achieved over a range of complex stoichiometries but is dependent on maintaining appropriate levels of the components Frizzled and Strabismus. We propose that the core proteins assemble into signalosome-like structures, where stable association is not dependent on one-to-one interactions with binding partners, and signaling functions can act over a wide range of complex compositions.

## Highlights

• Planar polarity proteins form signalosome-like complexes of variable stoichiometry
• Flamingo and Frizzled form a stoichiometric nucleus within the complex
• Asymmetric localization of core proteins is robust to changes in complex composition
• Core protein stability in complexes is not dependent on complex stoichiometry

## Graphical Abstract

**Figure:**
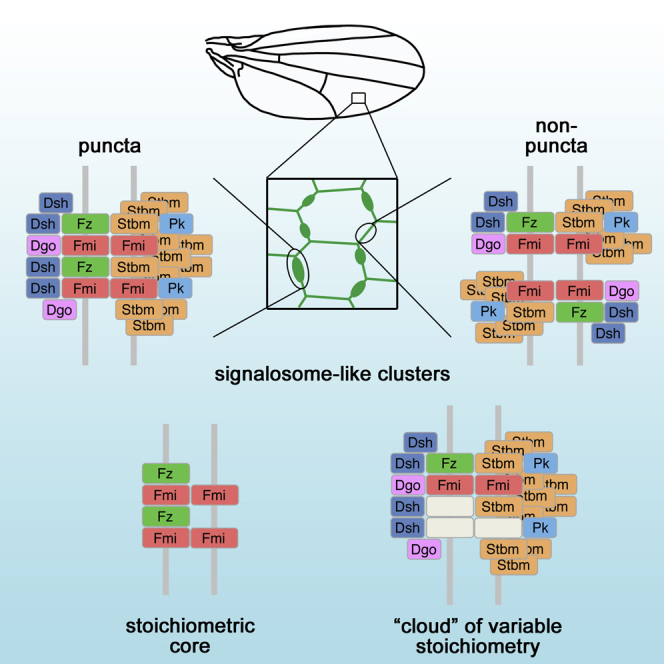

## Teaser

Strutt et al. show that the composition of the core planar polarity complex is highly plastic, with stable association of complex components occurring over a wide range of stoichiometries and with cell polarization being robust to changes in complex composition, suggesting a signalosome-like organization.

## Introduction

During animal development, many epithelia are polarized in the plane of the tissue. One of the best-characterized systems that controls this planar polarity involves the core planar polarity proteins (known hereafter as the core proteins). These localize asymmetrically within cells at the level of the adherens junctions and control the production of polarized structures and polarized cell behavior (Goodrich and Strutt, 2011, Wallingford, 2012).

**Figure 1:**
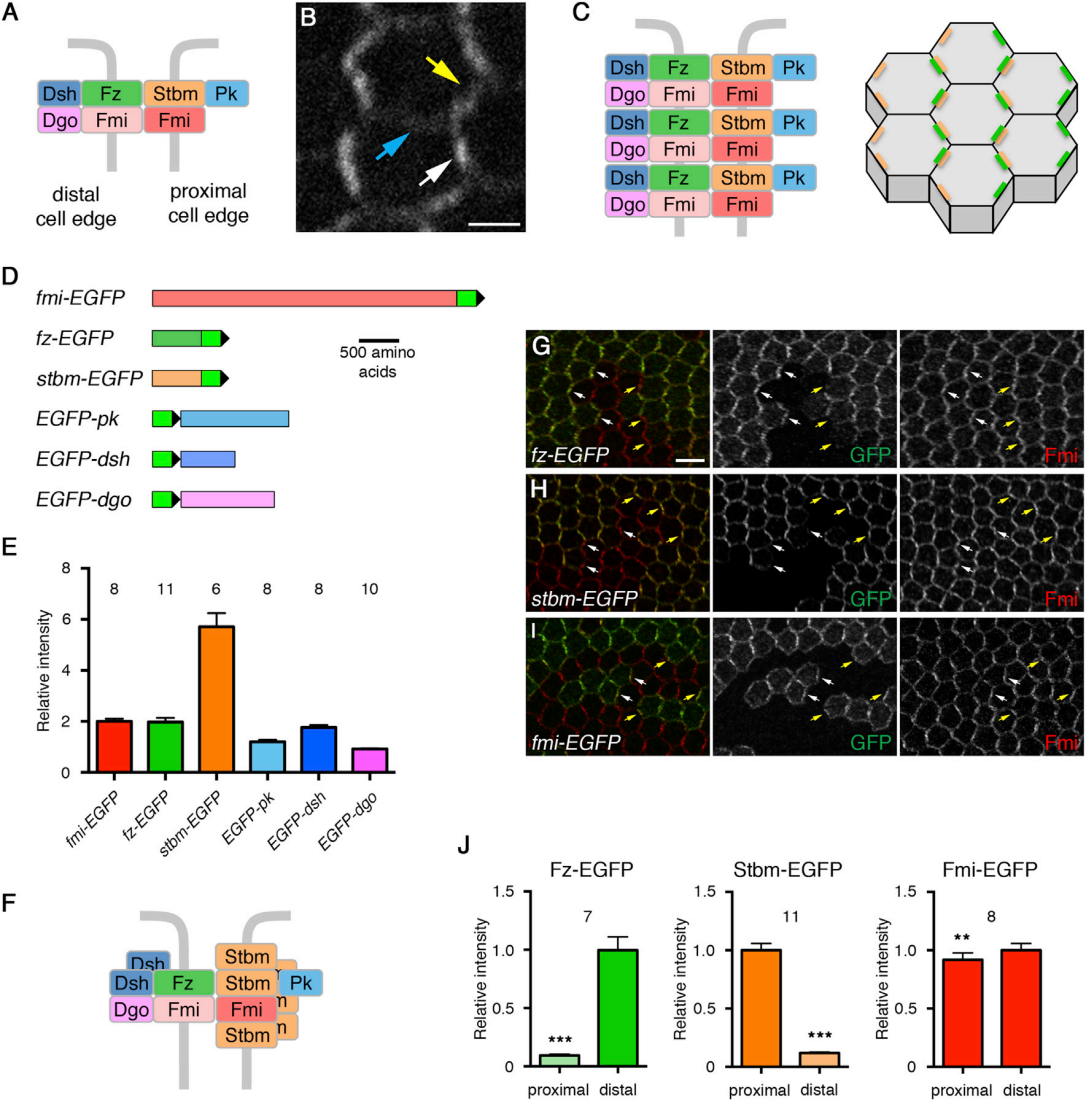
Core Planar Polarity Complex Stoichiometry (A) Diagram of the core proteins localizing to proximal and distal cell ends, based on known localizations and protein-protein interactions. (B) Live image of a *fmi-EGFP* pupal wing. Core proteins localize predominantly at the level of the apical adherens junctions. Arrows point to a punctum (white) and a non-punctum region (yellow) in the proximal-distal junctions and to a lateral junction (blue) where no puncta are seen. Proximal is to the left and distal is to the right in this and all later images. Scale bar, 2 μm. (C) Core protein complexes in the same orientation undergo local clustering in membrane subdomains (puncta, left), which leads to overall cellular asymmetry (right, where green represents distal Fz-containing complexes in puncta and orange represents proximal Stbm-containing complexes in puncta). (D) Diagram of the tagged core proteins. Bright green is EGFP, and the black triangle is the position of the residual *LoxP* site (not to scale). Fmi, Fz, Pk: EGFP tag inserted into the endogenous genomic locus by in vivo homologous recombination. Stbm, Dsh, Dgo: EGFP tag inserted into a P[acman] rescue construct. (E) Mean intensity of the EGFP-tagged core proteins in puncta at 28 hr after puparium formation (APF), normalized to 2 units of Fmi-EGFP. Flies were homozygous for the tagged gene. ANOVA analysis shows that Fmi/Fz/Dsh and Pk/Dgo are not significantly different to each other. On this and all subsequent graphs, the number of wings is indicated above the respective column. (F) Diagram of the core complex in puncta, based on stoichiometry data. Note half the amount of Fz is shown here, compared to the stoichiometry graph, to compensate for there being more Fz-EGFP than endogenous Fz in puncta. (G–I) High-resolution images of *fz-EGFP* (G), *stbm-EGFP* (H), and *fmi-EGFP* (I) twin clones with untagged protein, revealing asymmetric cellular localizations on clone boundaries. Arrows point to puncta on distal (white) or proximal (yellow) cell boundaries. Scale bar, 5 μm. (J) Mean intensity of puncta on proximal and distal cell edges. Fmi-EGFP is slightly enriched distally, where Fz is higher; also, Fmi-EGFP puncta are significantly smaller (84%, p = 0.02, paired t test) on proximal cell edges than on distal cell edges. ^∗∗∗^p < 0.001; ^∗∗^p < 0.01 (paired t test comparing proximal and distal puncta in the same wing). Error bars are SEM.

The core proteins have been well studied in the *Drosophila* pupal wing epithelium. Here, they localize asymmetrically on proximal and distal cell edges and regulate the orientation of an actin-rich trichome, which emerges from the distal end of each cell. The seven-pass transmembrane protein Frizzled (Fz) localizes distally, with the cytoplasmic proteins Dishevelled (Dsh) and Diego (Dgo); Strabismus (Stbm; also known as Van Gogh), a four-pass transmembrane protein, localizes proximally with the cytoplasmic protein Prickle (Pk); and the atypical cadherin Flamingo (Fmi; also known as Starry Night) localizes both proximally and distally, where it can mediate homophilic adhesion between neighboring cells (Figure 1A). Thus, the core proteins form an intercellular complex, bridging neighboring cells and allowing them to coordinate their polarity (Strutt and Strutt, 2009, Devenport, 2014).

Fmi and Fz form the essential nucleus of this complex. In the absence of Fmi activity, Fz, Dsh, and Dgo are lost from junctions (Axelrod, 2001, Feiguin et al., 2001, Shimada et al., 2001, Strutt, 2001) and Stbm and Pk levels are reduced (Bastock et al., 2003, Tree et al., 2002). In turn, if Fz is absent, Fmi localizes poorly to junctions and is predominantly found in the apical plasma membrane (Usui et al., 1999, Strutt and Strutt, 2008). Furthermore, the Fmi-Fz interaction is a key symmetry breaking step: Fmi localizes better to junctions between cells when Fz is only present in one cell than it does when Fz is present in both cells (Strutt and Strutt, 2008; see also Struhl et al., 2012). The activity of the other four core proteins is required to redistribute these Fmi:Fmi-Fz intercellular complexes so that they are localized at one end of the cell, in the same orientation, with the overall direction of polarity being dependent on upstream tissue-specific cues (Goodrich and Strutt, 2011, Devenport, 2014).

The molecular mechanisms that sort the core proteins to opposite cell ends are not understood. However, the core proteins can self-organize: clones of cells lacking Fz activity recruit core proteins to clone boundaries (Usui et al., 1999, Strutt, 2001), resulting in a reversal of polarity on one side of the clone, which can be propagated over several rows of cells (Vinson and Adler, 1987). Moreover, computational models have shown that positive and negative feedback interactions between the core proteins may be sufficient to amplify a slight bias in localization or activity of one of the proteins (e.g., Amonlirdviman et al., 2005, Le Garrec et al., 2006, Burak and Shraiman, 2009, Schamberg et al., 2010).

Cellular asymmetry of the core proteins correlates with their concentration into membrane subdomains that we term puncta (Figure 1B): asymmetry within puncta is greater than in other junctional regions (Strutt et al., 2011, Cho et al., 2015). Furthermore, core proteins within puncta are highly stable, with lower rates of turnover than elsewhere in the junctions (Strutt et al., 2011; see also Butler and Wallingford, 2015, Chien et al., 2015). Thus, we hypothesize that the feedback interactions that sort the core proteins onto proximal and distal membranes primarily act locally, resulting in the production of spatially distinct, polarized membrane subdomains (Figure 1C).

There are many unanswered questions about the nature of the core complex and how its components become sorted into puncta. First, the molecular interactions between the complex components are incompletely characterized, and the overall composition of the complex is not known. Stbm, Pk, Dsh, and Dgo can each interact directly with each of the others (Tree et al., 2002, Jenny et al., 2003, Jenny et al., 2005, Das et al., 2004), and the mouse Fz homolog Fzd5 can directly bind the Dsh homolog Dvl1 (Tauriello et al., 2012). Fmi and Fz, and mouse homologs of Fmi and Stbm (Celsr1 and Vangl2), have also been reported to co-immunoprecipitate (Chen et al., 2008, Devenport and Fuchs, 2008), and Stbm, Pk, and Dsh/Dvl can homodimerize (Jenny et al., 2003, Kishida et al., 1999). How these interactions translate into a functioning complex in vivo, and how they might promote sorting, remains unclear. Current hypotheses are that Pk and Stbm inhibit recruitment of Dsh to junctions by Fz (Amonlirdviman et al., 2005), that Dgo and Pk compete for binding to Dsh (Jenny et al., 2005), and that Pk mediates negative feedback interactions by excluding Stbm-Fmi complexes from junctions (Cho et al., 2015).

Second, it is not understood why the core proteins within puncta are more stable than those not in puncta. One possibility is that the composition of the complex might change as the core proteins become sorted into puncta. For example, there is evidence that the cytoplasmic proteins (Pk, Dsh, and Dgo) promote clustering (Feiguin et al., 2001, Tree et al., 2002, Bastock et al., 2003), so their levels might increase as core proteins enter puncta. Alternatively, if these cytoplasmic proteins mediate negative feedback interactions, their levels might decrease as asymmetry increases, as they are no longer needed.

Finally, it is not known why asymmetry is robust to changes in core protein levels, as such changes might be expected to disrupt feedback interactions. Loss of Dsh ubiquitination leads to the accumulation of Dsh and the other core proteins at junctions (Strutt et al., 2013a); nevertheless, only minor defects in core protein asymmetry are seen. However, the degree to which feedback is robust to changes in individual protein concentrations has not been systematically tested.

Here, we carried out a detailed study of core protein levels at cell junctions. We assumed that the proteins detected at junctions are all part of complexes, as genetic studies have shown that individual core proteins have little or no localization to junctions in the absence of their transmembrane partners (Axelrod, 2001, Feiguin et al., 2001, Shimada et al., 2001, Strutt, 2001, Tree et al., 2002, Bastock et al., 2003, Das et al., 2004, Strutt and Strutt, 2008). This allowed us to use relative concentrations at junctions to infer core complex composition. We examined protein levels both by immunolabeling of endogenous proteins and by live imaging of EGFP-tagged proteins. Immunolabeling shows qualitative differences in protein levels but is not fully quantitative, as detection may be non-linear, antibodies may saturate, and background staining may vary. For quantitative analyses, we measured the light intensity released from an EGFP tag by live imaging (Coffman and Wu, 2012). By comparing the fluorescence intensities of different tagged molecules, relative concentrations can be determined. This technique has been utilized in many contexts, from microorganisms to vertebrates (Chiu et al., 2002, Damle et al., 2006, McGill et al., 2009). Importantly, the amount of GFP fluorescence has been shown to increase linearly with the number of fluorescent molecules in vitro (Chiu et al., 2001) and in vivo (Wu and Pollard, 2005), suggesting that addition of the EGFP tag to different molecules does not affect EGFP fluorescence.

Using this methodology, we have determined the relative concentrations of each of the core proteins at junctions in pupal wings in vivo. By manipulating gene dosage, we then investigated how modulating complex composition affects asymmetry. This gives us unexpected insights into the relationships among core complex composition, sorting into asymmetric junctional puncta, and the acquisition of cellular asymmetry, and it leads us to suggest that the complex is organized into signalosome-like structures.

## Results

### In Vivo Stoichiometry of the Core Planar Polarity Proteins in Puncta

To determine the relative stoichiometry of the core planar polarity proteins in vivo, we tagged each of them with EGFP (Figure 1D) and then imaged pupal wings expressing each tagged protein under the same conditions.

For this approach, the tagged proteins must all be expressed at endogenous levels. For Fmi, Fz, and Pk, the EGFP tag was inserted into the endogenous locus by in vivo homologous recombination. For Stbm, Dsh, and Dgo, the EGFP tag was inserted into a P[acman] rescue construct by recombineering and then integrated into the genome; the transgenic animals were then crossed into appropriate mutant backgrounds to maintain normal gene dosage.

The EGFP-tagged proteins localized asymmetrically at junctions in the pupal wing, and trichome polarity in the adult fly wing was normal, suggesting that the proteins can replace endogenous protein function (Figure S1). Western blotting was used to compare the overall cellular levels of core proteins in pupal wings expressing only the tagged protein to those expressing only endogenous protein (Figure S2). Finally, twin clone experiments were used to compare the amount of tagged and untagged protein in junctional puncta, as well as the degree of asymmetry (Figure S1; Tables S1 and S2). Five of the core proteins behaved similarly to the endogenous proteins in these assays. However, Fz-EGFP was expressed at higher levels than endogenous Fz as detected by western blotting, and more was seen in puncta, which we speculate may be because the EGFP tag alters Fz stability. Therefore, measurements of core protein stoichiometry will show artificially high levels of Fz-EGFP. Importantly, however, for all the core proteins, the intensity of EGFP fluorescence in flies expressing one copy of tagged protein and one copy of endogenous protein was approximately half that of flies expressing two copies of tagged protein (Table S1). This suggests that the tagged proteins compete similarly to the endogenous proteins for inclusion into puncta.

We first measured the stoichiometry of the core proteins within puncta, as core proteins in puncta are predominantly stable and highly asymmetrically organized, consistent with ordered arrays of aligned complexes (Figure 1C). As a control, we ascertained that all puncta have similar compositions; co-immunolabeling pupal wings for Fmi and each of the EGFP-tagged core proteins showed that Fmi intensity in different puncta increased linearly with EGFP intensity (Figure S3). Live imaging of pupal wings was then carried out on flies expressing each of the tagged core proteins, and the mean intensity of EGFP fluorescence in puncta was determined (see Experimental Procedures).

Comparing mean EGFP puncta intensity in each of the fly lines showed that for every two molecules of Fmi-EGFP within puncta, there are approximately two molecules of Fz-EGFP and EGFP-Dsh, six molecules of Stbm-EGFP, and one molecule of EGFP-Pk and EGFP-Dgo (Figure 1E). As more Fz-EGFP than endogenous Fz is seen in puncta in twin clone experiments (Figure S1; Table S1), we estimate that the amount of endogenous Fz in puncta would be closer to one molecule for every two Fmi molecules. Therefore, instead of puncta exhibiting one-to-one ratios of the different components (e.g., Figure 1A), we see a more complex organization, as summarized in Figure 1F.

We then investigated the degree of asymmetry of the core proteins within puncta by making twin clones and examining puncta intensity on clone boundaries, where tissue expressing tagged protein was adjacent to tissue expressing untagged protein. As previously observed (Strutt et al., 2011), Fz-EGFP is highly enriched in distal puncta compared to proximal puncta, while the reverse is seen for Stbm-EGFP. Fmi-EGFP levels are similar in proximal and distal puncta (Figures 1G–1J). This is consistent with the view that within puncta core proteins are assembled into complexes of the approximate composition shown in Figure 1F, aligned in a common orientation.

### Core Protein Stoichiometry Is Similar in Puncta and Non-puncta Junctional Domains, Despite Differing Stable Protein Proportions

**Figure 2:**
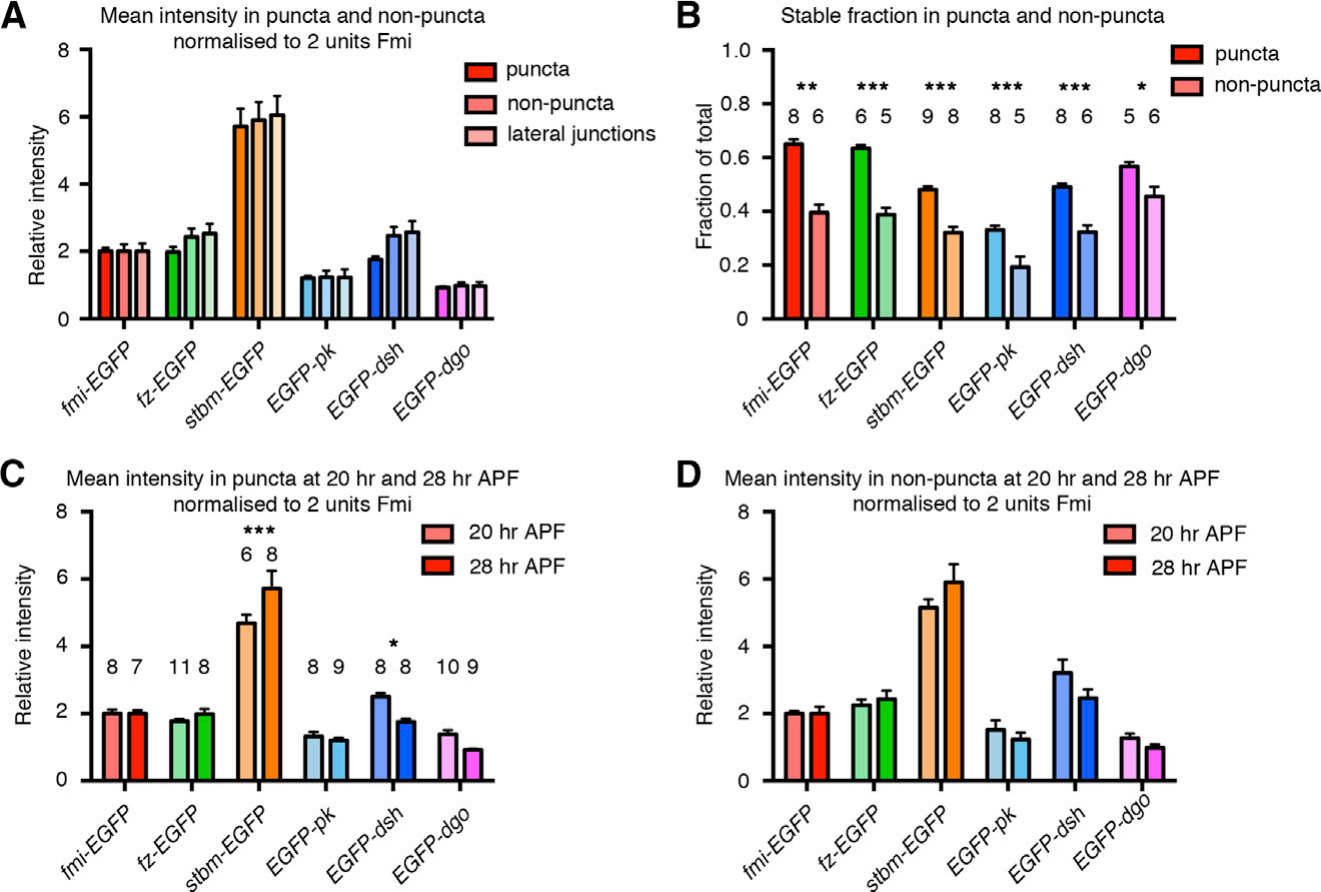
Stoichiometry and Stability in Puncta and Non-puncta Junctional Domains (A) Mean intensity of EGFP fluorescence in puncta, non-puncta and lateral junctions at 28 hr APF, normalized to 2 units of Fmi-EGFP in each region. Slight increases in the relative levels of Fz-EGFP and EGFP-Dsh are seen in non-puncta and lateral junctions, but these are not statistically significant (comparing puncta and lateral junctions, p = 0.17 for Fz-EGFP and p = 0.06 for EGFP-Dsh, two-way ANOVA). Sample sizes as in Figure 1E. (B) Stable proportions of each tagged protein, as determined by FRAP analysis at 28 hr APF, in puncta and non-puncta. ^∗∗∗^p < 0.001; ^∗∗^p < 0.01; ^∗^p < 0.05 (curve plateaux compared using an extra sum-of-squares F test). (C and D) Mean intensity of EGFP-tagged core proteins in puncta (C) and non-puncta (D) at 20 hr APF and 28 hr APF, normalized to 2 units of Fmi-EGFP. ^∗∗∗^p < 0.001; ^∗^p < 0.05 (20 hr and 28 hr values compared by two-way ANOVA). Error bars are SEM.

In junctional domains that lack large puncta, core protein complex distribution is less ordered, and complexes are less stable (Strutt et al., 2011; see below). We hypothesized that core complexes in these regions might not be fully assembled and the stoichiometry of the complex in these regions might therefore be different. To test this, we determined the intensity of fluorescence in junctions, excluding the puncta. As this may not fully exclude smaller puncta from the “non-puncta” regions, we also measured the fluorescence intensity on lateral junctional domains, where puncta are not observed (Figure 1B; Strutt et al., 2011). Puncta were roughly three times as bright as non-puncta and lateral cell junctions (Figure S4A), but no significant difference was observed in the relative stoichiometry of the core proteins in puncta, non-puncta, or lateral junctions (Figure 2A).

The similar relative core protein levels and corresponding complex composition in puncta and non-puncta were surprising, considering that Fz and Fmi show lower stability in non-puncta (Strutt et al., 2011). We therefore investigated whether all core proteins show reduced stability in non-puncta regions.

Stable proportions of the endogenously expressed tagged core proteins were determined using fluorescence recovery after photobleaching (FRAP). All six core proteins had a larger stable proportion in puncta than non-puncta, although the stable proportion varied for each protein (Figure 2B). Interestingly, Fmi and Fz had similar stable proportions in puncta and also smaller similar stable proportions in non-puncta, consistent with them forming a nucleus to the complex (Strutt and Strutt, 2008).

In summary, overall core protein stoichiometry is similar regardless of whether complexes are concentrated in stable ordered domains (puncta) or more sparsely distributed in less ordered non-puncta regions. However, the stable and unstable proportions of the core proteins vary between puncta regions and non-puncta regions. This indicates an uncoupling between complex composition and protein stability, such that stability is not promoted by an altered complex composition but by the local concentration of complexes of the same orientation.

### Stoichiometry Is Different at an Earlier Stage of Development

As core complex stoichiometry is similar in puncta and non-puncta, one possibility was that a single optimal composition is necessary for junctional localization of the core protein complex. If this were true, stoichiometry would also be the same in tissue in which core protein localization is overall less asymmetric. At earlier stages of wing development, cells are undergoing junctional remodelling; the core proteins show only weak cellular asymmetry and form only small puncta (Aigouy et al., 2010, Strutt et al., 2011).

Interestingly, complex stoichiometry was different in earlier-stage wings. The relative amount of Stbm in puncta was lower in younger wings, while the relative amount of Dsh was increased (Figure 2C). Similar trends were seen in non-puncta, but the differences were not statistically significant (Figures 2D, S4B, and S4C). We conclude that in fact complex stoichiometry is not fixed.

### The Stoichiometry of the Cytoplasmic Proteins in Complexes Is Dependent on Expression Levels

**Figure 3:**
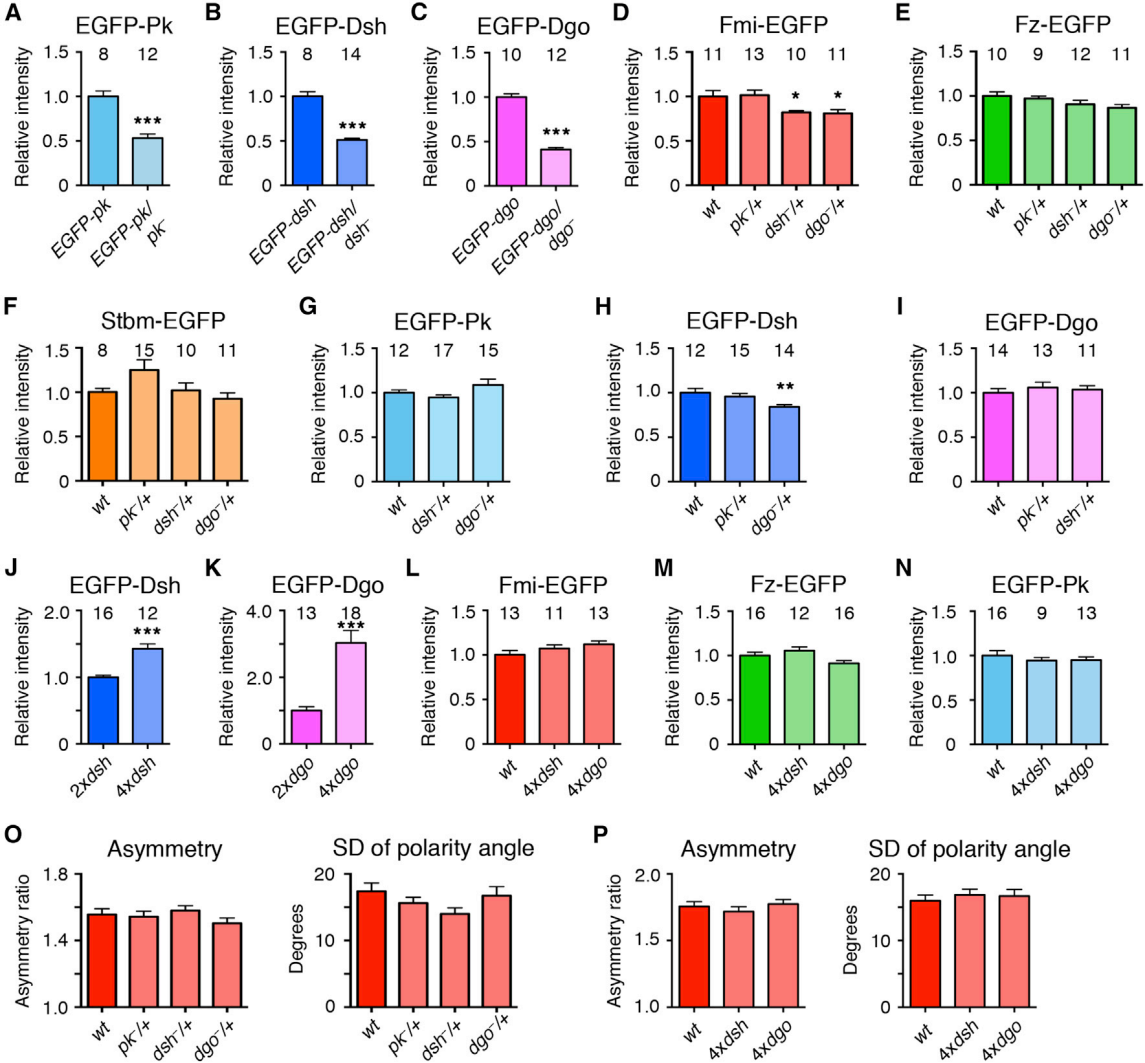
The Effects on Complex Stoichiometry of Altering Gene Dosage of Cytoplasmic Core Proteins (A–C) Relative mean intensity of puncta in live images of wings homozygous for the indicated EGFP-tagged core gene or carrying one copy of the EGFP-tagged gene in a null mutant background. ^∗∗∗^p < 0.001 (ANOVA comparison to wild-type). (D–I) Relative mean intensity of puncta in live images of wings carrying EGFP-tagged core proteins, in a wild-type background, or in wings heterozygous for *pk-sple*^*13*^, *dsh*^*V26*^, or *dgo*^*380*^. ^∗∗^p < 0.01; ^∗^p < 0.05 (ANOVA comparison to wild-type). (J and K) Relative mean intensity of puncta in live pupal wings, comparing flies carrying one dose of endogenous gene and one dose of tagged gene with those carrying two doses of endogenous gene and two doses of tagged gene. Note that when *dgo* dosage is doubled, three times as much Dgo enters puncta, possibly due to cooperative effects. ^∗∗∗^p < 0.001 (unpaired t test). (L–N) Relative mean intensity of Fmi-EGFP (L), Fz-EGFP (M), and EGFP-Pk (N) puncta in live images of wild-type wings or wings homozygous for *P[acman]-dsh* or *P[acman]-dgo*. Data compared to wild-type by ANOVA. (O and P) Fmi-EGFP asymmetry and SD of polarity angle in images from live pupae of wings heterozygous for *pk-sple*^*13*^, *dsh*^*V26*^, and *dgo*^*380*^ (O) or in flies homozygous for *P[acman]-dsh* or *P[acman]-dgo* (P) (see Experimental Procedures for details of polarity quantitation). There were no significant differences to wild-type (ANOVA). Sample sizes as in (D) and (L). Error bars are SEM.

We next investigated what happens to stoichiometry if gene dosage of one of the core proteins is altered. First, we examined the effects of halving the dosage of the three cytoplasmic core proteins (Pk, Dsh, or Dgo). Pupal wings expressing two copies of EGFP-tagged protein and no endogenous protein were compared to wings carrying one copy of tagged protein in a heterozygous null mutant background. In each case, the levels of EGFP-tagged protein at junctions was roughly halved in the heterozygous mutant (Figures 3A–3C; Table S3). This was true for both puncta or non-puncta, suggesting that the amount of the cytoplasmic proteins is limiting and that furthermore, under conditions of reduced protein, there is not preferential recruitment to puncta. This was confirmed by immunostaining endogenous proteins (Figures S5A–S5C). Interestingly, halving the junctional amount of any of the cytoplasmic proteins had little effect on the levels of any of the other core proteins, either in puncta or non-puncta (Figures 3D–3I; Table S3).

P[acman] constructs in wild-type instead of mutant backgrounds were then used to double gene dosage. Doubling Dsh or Dgo dosage caused the amount of protein in puncta to increase, consistent with idea that levels of these components are normally limiting (Figures 3J, 3K, and S5D–S5F). Again, this did not alter the levels of other core proteins (Figure 3L–3N; Table S3). This indicates that excess cytoplasmic protein can enter core protein complexes, resulting in variable stoichiometries relative to the transmembrane proteins that recruit them, and that their levels in puncta are limited by their concentration within the cell rather than by specific binding partners.

Computational modeling has suggested that the cytoplasmic proteins play key roles in promoting feedback interactions between the core proteins (e.g., Amonlirdviman et al., 2005, Le Garrec et al., 2006, Burak and Shraiman, 2009, Schamberg et al., 2010). However, modulating their levels in puncta had no effect on core protein asymmetry (Figures 3O and 3P), indicating that feedback and generation of asymmetry does not depend on an exact ratio or concentration of particular cytoplasmic core proteins at the cell junctions.

### The Core Complex Is Assembled Around a Stoichiometric Frizzled-Flamingo Nucleus

**Figure 4:**
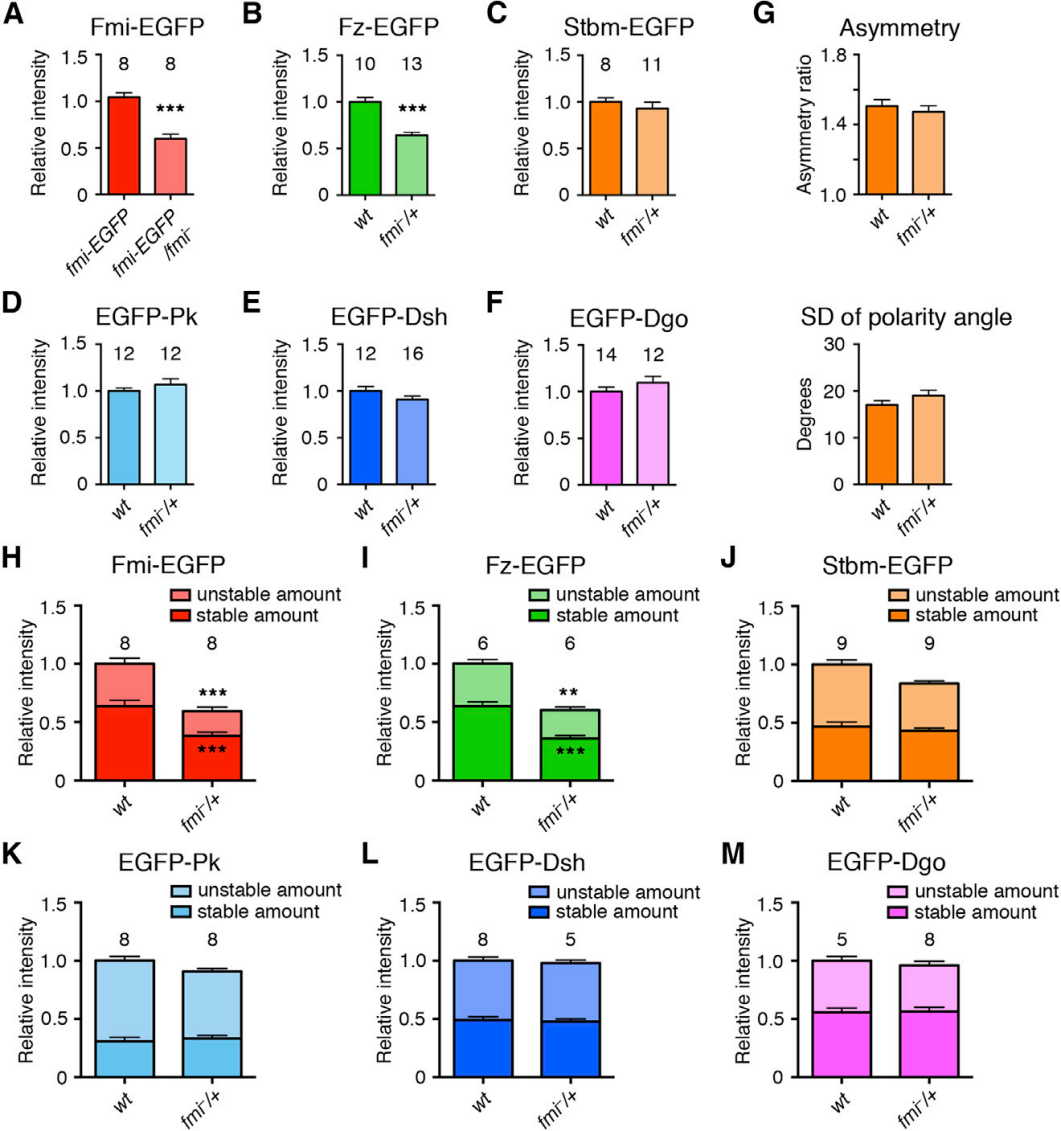
The Core Complex Contains a Stoichiometric Nucleus of Flamingo and Frizzled (A–F) Relative mean intensity of puncta in wings carrying EGFP-tagged core proteins in a wild-type background or in wings heterozygous for *fmi*^*E59*^. ^∗∗∗^p < 0.001 (ANOVA). (G) EGFP asymmetry and SD of polarity angle in live images of wings expressing *stbm-EGFP*, in a wild-type background, or in flies heterozygous for *fmi*^*E59*^ (as above). Similar results were seen imaging *fmi-EGFP*. No significant difference between wild-type and mutant was seen (ANOVA). (H–M) Stable and unstable amounts of EGFP-tagged core proteins in puncta in a wild-type background or in wings heterozygous for *fmi*^*E59*^. Stable proportions, as determined by FRAP, were multiplied by total puncta intensity. Unpaired t tests were used to test for significance of changes in the stable amount (lower asterisks) or the total amount of protein (upper asterisks). ^∗∗∗^p < 0.001; ^∗∗^p < 0.01.

We then examined what happens to the other core proteins if the dosage of Fmi is altered. As with the cytoplasmic core proteins, halving *fmi* dosage caused a reduction in protein levels, both in puncta and non-puncta (Figures 4A and S5G; Table S3). Notably, halving the amount of Fmi at junctions caused a corresponding decrease in levels of Fz-EGFP (Figures 4B and S5H; Table S3). This suggests that Fmi levels control how much Fz enters the complex, consistent with the idea that Fmi and Fz form a stoichiometric nucleus for the complex. In contrast, levels of the other complex components were negligibly affected (Figures 4C–4F, S5I, and S5J; Table S3), and cellular asymmetry was also unaffected (Figure 4G).

*fmi* gene dosage was also increased by expressing a *P[acman]-fmi-EGFP* rescue construct in a wild-type background. This caused an increase in cellular protein levels but only a very mild increase in levels of Fmi in puncta (Figures S5K and S5L; Table S3), suggesting that the amount of Fmi that can enter puncta is limited.

We hypothesized that the plasticity in puncta composition might be explained by differences in core protein stability. In particular, the stable amounts of the core proteins might maintain a constant stoichiometry relative to each other, but this might be masked by the presence of an additional unstable population that varies depending on cellular concentration.

We first considered whether decreasing Fmi levels in the cell and hence in puncta might primarily result in a loss of the unstable Fmi in puncta, and thus, there would be an increase in the proportion of stable Fmi. FRAP analysis showed that this was not the case: when Fmi levels were decreased by halving *fmi* dosage, its stable proportion was unaltered, and thus, both the stable and unstable amounts were decreased (Figure 4H). Similarly, the stable proportion of Fz was also unchanged in *fmi* heterozygotes, leading to smaller stable and unstable amounts (Figure 4I). Thus, the stable ratios of Fmi and Fz remain constant.

We next examined whether the stable proportions of Stbm, Pk, Dsh, and Dgo decrease when *fmi* dosage is lowered to maintain a constant ratio of stable protein. Surprisingly, the stable amounts of these proteins were unaltered (Figures 4J–4M), indicative of varying stable ratios relative to Fmi and Fz. Taken together, these results are consistent with the conclusion that Fmi and Fz maintain a stoichiometric, stable nucleus, while the levels and stability of the other core proteins can vary independently of this.

### Maintaining Levels of Frizzled and Strabismus within the Complex Is Essential for Strong Asymmetry

Halving *fz* dosage had little effect on its levels in puncta (Figure S6A), and in western blots, no significant decrease in the amount of Fz in the cell was seen (Figure S6E). As some Fz is normally targeted to the lysosome (Strutt and Strutt, 2008), degradation of excess protein may be reduced when gene dosage is lowered in order to maintain levels at junctions. Interestingly, increased Fz levels in puncta (as a result of the higher cellular levels of Fz-EGFP compared to endogenous Fz; see Figure S1H) had no effect on the levels of other core proteins tested (Figures S6C and S6D). In particular, Fmi levels are not increased, suggesting that although there is a stoichiometric nucleus of one molecule of Fz to two molecules to Fmi, such that a minimum threshold level of Fmi is required to initiate Fz recruitment (Figure 4B), once this nucleus is present above a certain concentration, more Fz can become incorporated into complexes.

Halving *stbm* dosage also did not affect Stbm levels in puncta (Figure S6B), although in this case, cellular levels were decreased (Figure S6F). Furthermore, doubling *stbm* gene dosage increased the amount in the cell (Figure S6F), but no more was seen in puncta (Figure S6G). This suggests that Stbm protein is in excess in the cell, but no more can enter junctional complexes.

**Figure 5:**
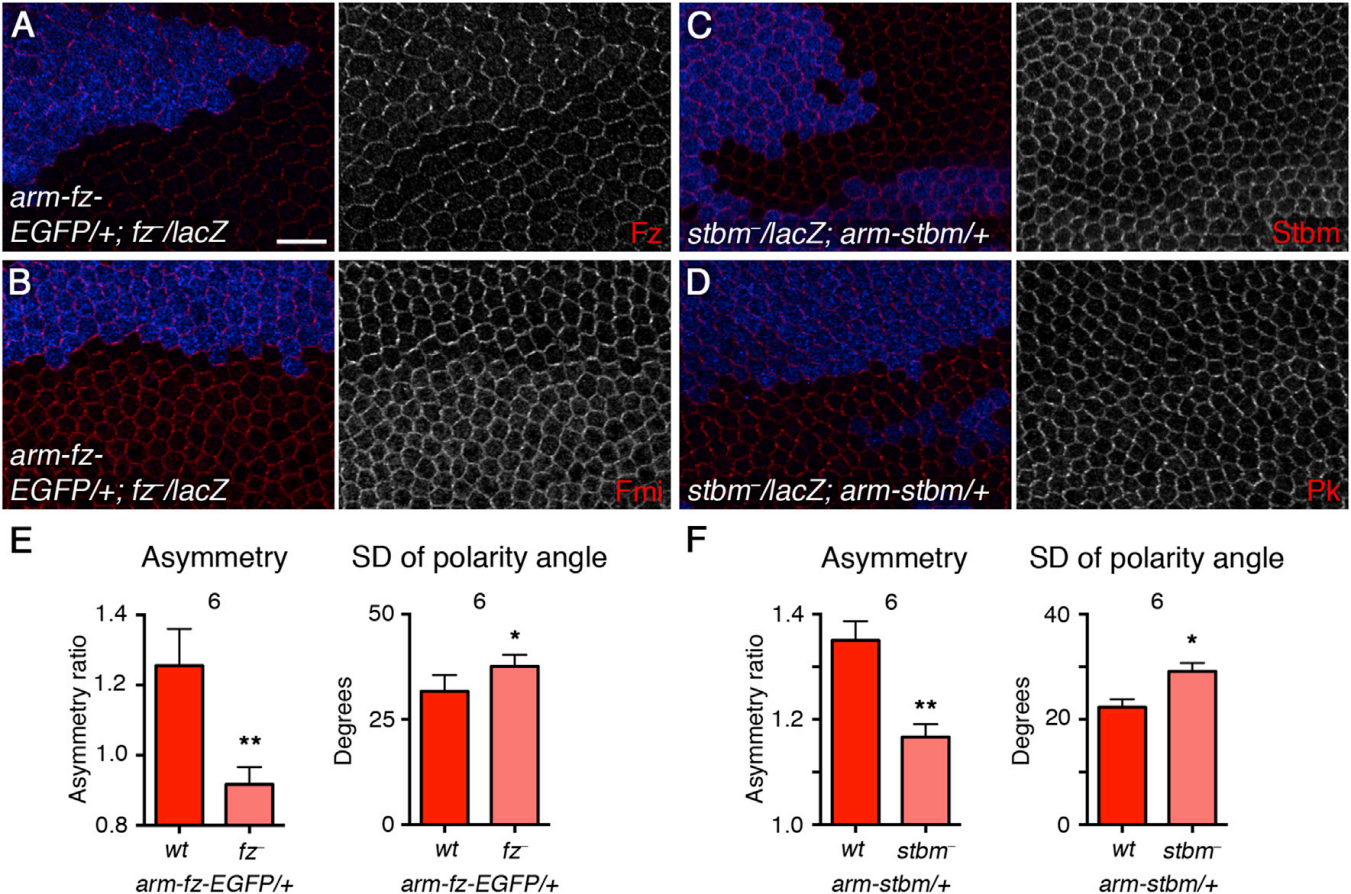
Decreasing Cellular Levels of Frizzled or Strabismus Causes Defects in Asymmetry (A and B) Pupal wings carrying one copy of *arm-fz-EGFP*, with *fz*^*P21*^ clones marked by loss of β-gal staining (blue). Wings stained for Fz (A, red) or Fmi (B, red). Note that higher levels of the Fz-EGFP fusion used here have been shown to function normally (Strutt, 2001; Figure S7K). Scale bar, 10 μm. (C and D) Pupal wings carrying one copy of *arm-stbm*, with *stbm*^*6*^ clones marked by loss of β-gal staining (blue). Wings stained for Stbm (C, red) or Pk (D, red). (E and F) Asymmetry and SD of polarity angle. (E) Pupal wings carrying one copy of *arm-fz-EGFP*, in *fz*^*P21*^ clones or in twinspot tissue (wild-type background). (F) Pupal wings carrying one copy of *arm-stbm*, in *stbm*^*6*^ clones or in twinspot tissue (wild-type background). Wings were immunostained for Fmi, but similar results were obtained by Fz or Stbm immunostaining. Note that the overall variation in the polarity angle is high in wild-type tissue next to *fz*^*P21*^ clones (averaging 30°), because of the strong boundary effects, leading to non-autonomous defects on the orientation of core protein localization outside of the clone. ^∗∗^p < 0.0; ^∗^p < 0.05 (paired t test used to compare polarity in wild-type tissue and mutant clones in the same wing). Error bars are SEM.

In order to test if maintaining levels of Fz and Stbm in puncta is important for feedback and generation of asymmetry, we expressed both proteins at artificially low levels using single copies of transgenes under control of the *armadillo* (*arm*) promoter, and we then examined their levels in clones lacking endogenous protein. In both cases, the levels of protein in puncta were modestly decreased (Figures 5A and 5C; Table S4). This caused only minor changes in junctional levels of the other core proteins (Figures S7A–S7H; Table S4). However, reduced levels of Fz led to some Fmi localizing at the apical plasma membrane, as in *fz* mutant tissue (Figure 5B, Strutt and Strutt, 2008). A slight increase in Pk levels was seen when Stbm levels in puncta were lowered, consistent with Stbm negatively regulating Pk levels (Figure 5D, Strutt et al., 2013b).

Strikingly, when the amount of either Fz or Stbm in puncta was lowered, even by a modest degree, asymmetry was significantly reduced (Figures 5E and 5F) and trichome orientation defects were seen (Figures S7I and S7J). Therefore, while levels of the other core proteins in the complex can be altered without affecting asymmetry, reduced levels of Fz and Stbm relative to their binding partners cause strong defects in cell polarization.

### Asymmetry Is Sensitive to Large Modulations of Core Complex Stoichiometry

**Figure 6:**
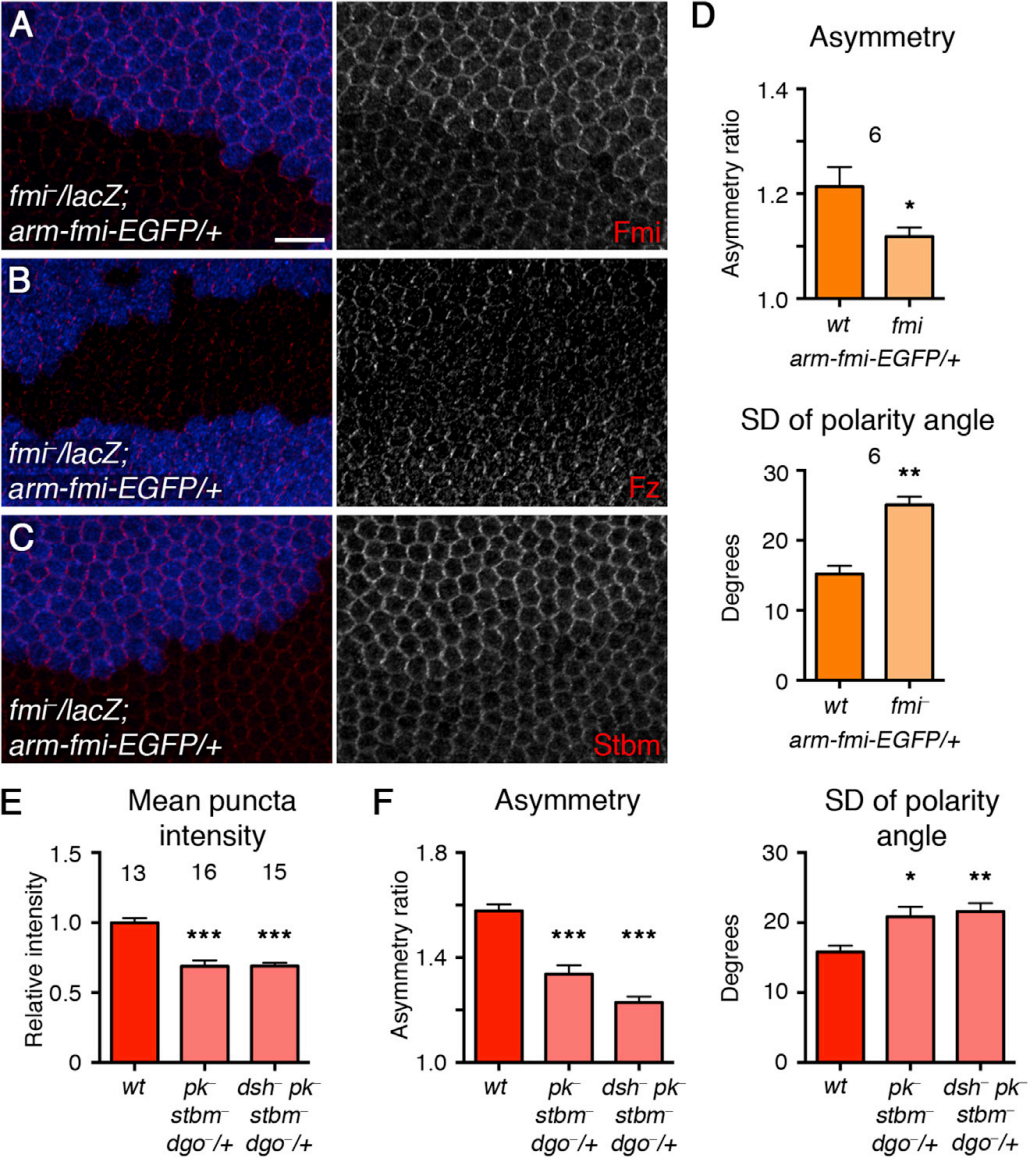
The Effects of Large Decreases in Flamingo Levels and Large Modulations in Core Complex Stoichiometry (A–C) Pupal wings carrying one copy of *arm-fmi-EGFP*, with *fmi*^*E59*^ clones marked by loss of β-gal staining (blue). Wings labeled for Fmi (A), Fz (B), and Stbm (C) in red. Scale bar, 10 μm. (D) Asymmetry and SD of polarity angle for wings carrying one copy of *arm-fmi-EGFP*, in *fmi*^*E59*^ clones or in twinspot tissue (wild-type background). Wings were immunostained for Stbm, but similar results were obtained by immunostaining for other core proteins. ^∗∗^p < 0.01; ^∗^p < 0.5 (paired t test used to compare polarity in wild-type tissue and mutant clones in the same wing). (E) Relative mean intensity of puncta in *fmi-EGFP/+* live pupal wings in a wild-type background and in flies triply heterozygous for *pk-sple*^*13*^, *stbm*^*6*^, and *dgo*^*380*^ or quadruply heterozygous for *dsh*^*V26*^, *pk-sple*^*13*^, *stbm*^*6*^, and *dgo*^*380*^. ^∗∗∗^p < 0.001 relative to wild-type (ANOVA). (F) Asymmetry and SD of polarity angle, measuring EGFP fluorescence in the same genotypes as in (E). ^∗∗∗^p < 0.001; ^∗∗^p < 0.01; ^∗^p < 0.05 relative to wild-type (ANOVA). Error bars are SEM.

Finally, we asked whether asymmetry can also be disrupted by more severe alterations in core complex composition. As halving *fmi* dosage did not affect asymmetry, we lowered *fmi* dosage further, by expressing a single copy of *fmi* under the *arm* promoter, in *fmi* mutant clones. A clear decrease in the levels of Fmi, Fz, and Stbm was observed by immunostaining (Figures 6A–6C; Table S4), but there was only a mild reduction in levels of Pk, Dsh, and Dgo (Figures S7L–S7N; Table S4). However, core protein asymmetry was reduced (Figure 6D), suggesting that a threshold amount of Fmi is necessary for robust polarization. However, this degree of reduced asymmetry was not sufficient to cause defects in trichome orientation (Figure S7O).

Second, we examined the effects of halving the dosage of multiple core genes simultaneously. Notably, while the levels of Fmi in puncta were only mildly altered when the dosage of a single core gene was halved (Figure 3D), Fmi levels were significantly decreased in triple or quadruple heterozygotes (Figure 6E). This was accompanied by a decrease in asymmetry (Figure 6F, mild defects in trichome orientation are seen; see Figures S7P and S7Q). Therefore, we conclude that the composition of the core complex can vary considerably without any deleterious effects on overall asymmetry, but the extent of this plasticity is limited to within-threshold levels of each protein.

## Discussion

There are two key challenges for the core pathway in coordinating cell polarity within developing tissues: first to be able to respond dynamically to polarizing cues, and second to establish a sufficiently stable polarized state. These two requirements act in tension and suggest that pathway organization requires features in common both with rapidly responding signaling pathways and with long-lasting structural components of cells. To investigate how the core pathway achieves this balance, we have investigated the in vivo organization of the core planar polarity complex and how this translates into sorting of core proteins into stable membrane subdomains and cellular asymmetry.

Using quantitative imaging of the core complex in pupal wings, we first determined the in vivo stoichiometry of the complex and further showed that this is not fixed, with levels of the cytoplasmic components and Stbm able to vary relative to levels of Fz and Fmi. In contrast, levels of Fz and Fmi are interdependent, consistent with the notion that these proteins form a stoichiometric nucleus within the complex. Importantly, the stable proportions of core proteins at junctions are maintained over a range of different complex stoichiometries. Furthermore, complex composition is the same in more stable locally polarized domains and less stable unpolarized domains; thus, the size of the stable proportions is correlated with local order of polarity, not complex composition. Finally, asymmetry is robust to changes in complex composition, but the amount of Fz and Stbm within the complex must be maintained relative to their binding partners for normal asymmetry.

A caveat to our approach is that our ability to measure local complex stoichiometry is limited to the resolution of confocal microscopy (∼200 nm), whereas protein complexes would be expected to be at least an order of magnitude smaller. Nevertheless, the lack of variation in our measurements between different puncta and in junctions overall suggests that there is little spatial variation in complex composition in cell junctions, and the figures we obtain can be regarded as plausible local averages of complex composition.

**Figure 7:**
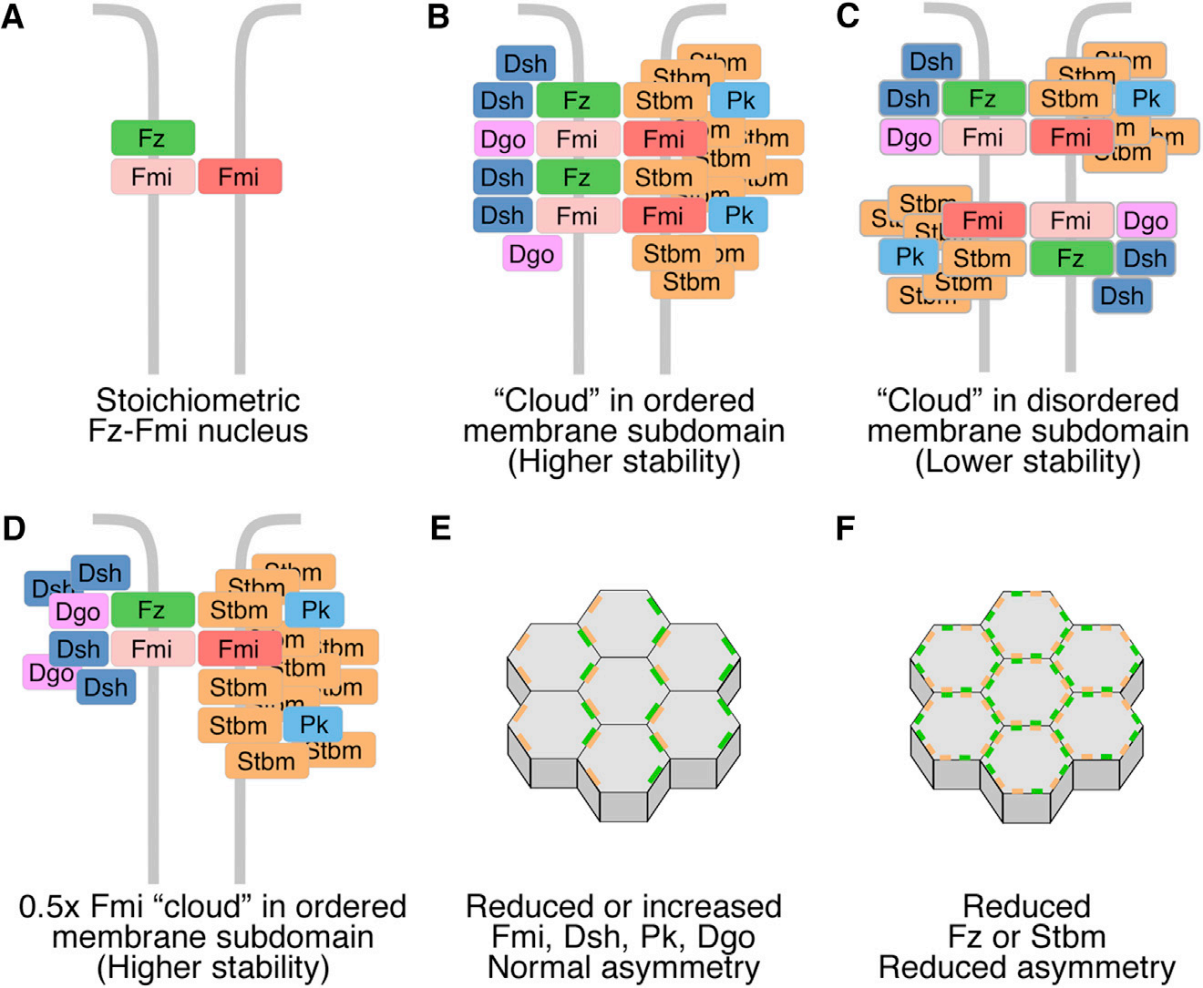
Cloud Model of Core Planar Polarity Complex Composition (A) Fz levels are dependent on Fmi levels, consistent with Fmi and Fz forming a stoichiometric nucleus to the complex. (B and C) Above a threshold concentration of Fmi and Fz at junctions, the core proteins form a signalosome-like cloud around the Fz-Fmi nucleus. Puncta contain ordered arrays of complexes of the same composition, which show higher stability, possibly due to cooperative interactions (B). Non-puncta have complexes of the same overall composition as puncta, but they are less ordered and less densely packed and core proteins are less stable (C). (D) The amount of core proteins associating in this cloud at junctions is dependent on their cellular concentration, but not on stoichiometric interactions with the Fz-Fmi nucleus, resembling the organization seen in signalosomes. For instance, halving Fmi levels reduces the amount of Fmi and Fz at junctions but does not alter the amounts of the other core proteins or their stability. (E) Altering levels of Fmi, Pk, Dsh, or Dgo at junctions alters the stoichiometry of the complex, but asymmetry is normal. (F) Reducing levels of Fz and Stbm at junctions disrupts asymmetry.

The cytoplasmic core proteins depend on the transmembrane proteins for their recruitment to junctions (Axelrod, 2001, Feiguin et al., 2001, Shimada et al., 2001, Tree et al., 2002, Bastock et al., 2003, Das et al., 2004). However, we find that relative levels of individual cytoplasmic proteins within the complex can increase or decrease independently of the other proteins. For example, Dsh and Dgo can be present at levels that are several-fold higher than the levels of their binding partners Fz and Fmi. We therefore suggest a “cloud model,” whereby complex composition is determined by cellular concentration rather than by stoichiometric binding interactions. We propose that the core proteins need a minimum concentration of Fmi and Fz to nucleate at junctions. Above this threshold concentration, multiple binding interactions allow a cloud of Stbm, Pk, Dsh, and Dgo to associate at junctions (Figures 7B–7D). Binding sites have been mapped between Stbm and Pk, as well as between Dsh and Dgo (Jenny et al., 2003, Jenny et al., 2005), and Stbm, Pk, and Dsh contain dimerization motifs (Jenny et al., 2003, Kishida et al., 1999). In addition to this, Fmi, Fz, Stbm, and Dsh contain putative PDZ (PSD-95, Discs Large, ZO-1) binding motifs of unknown function, which may interact with PDZ-containing scaffolding factors (Wolff and Rubin, 1998, Djiane et al., 2005, Wasserscheid et al., 2007, Johnston et al., 2013). Membrane interaction motifs, for example the DEP (Dishevelled, Egl10, and Pleckstrin) domain (Simons et al., 2009) and the Pk prenylation motif (Jenny et al., 2003, Lin and Gubb, 2009, Strutt et al., 2013b), may also promote association of the core proteins to junctions independently of protein-protein interaction sites. A further possibility is that once recruited to the complex, locally occurring post-translational modifications may alter protein mobility, allowing complex components to remain associated even in the absence of an ongoing direct interaction with a binding partner. For example, Dsh is phosphorylated only upon recruitment to junctions by Fz (Axelrod, 2001, Shimada et al., 2001).

Interestingly, the features we describe in our cloud model bear a striking resemblance to those seen for higher-order assemblies of signaling molecules known as signalosomes (Bienz, 2014, Wu and Fuxreiter, 2016). Such complexes are thought to be dynamic clusters of signaling molecules that typically assemble at ligand-bound receptors. Polymerization of signaling molecules into signalosomes increases their local concentration, often non-stoichiometrically, and cooperativity of assembly allows threshold responses to ligand stimulation. We therefore propose that core proteins assemble into signalosome-like structures, but importantly, unlike in the conventional view of signalosomes, core protein assembly is not a transient response to ligand binding but part of the process of robust establishment of stable cell polarity.

Another feature of signalosomes is that the polymerization of downstream signaling proteins is nucleated by a core that is often sub-stoichiometric (Wu and Fuxreiter, 2016)—a role that could be fulfilled by Fmi and Fz. We previously proposed that Fmi and Fz form a nucleus for the complex, which is key for symmetry breaking (Figure 7A; Strutt and Strutt, 2008). Consistent with this, we now show that Fz and Fmi levels at junctions have a stoichiometric relationship. Furthermore, FRAP analysis shows that similar proportions of Fmi and Fz are stable, both in puncta and non-puncta.

One of our original hypotheses was that the overall composition of the core complex might be different in highly polarized puncta, where protein stability is high, than in disordered non-puncta, where stability is lower. However, we find that the composition of the complex is very similar in puncta and non-puncta. Why then is core protein stability higher in puncta?

We suggest that there are two levels of organization of the core proteins. In both puncta and non-puncta, they nucleate around Fz-Fmi backbones into sub-microscopic signalosome-like structures of similar composition (Figures 7B and 7C). Then, under the influence of positive feedback interactions, some of these domains grow into locally ordered puncta of the same composition but higher stability. Clustering into puncta is most likely a result of cooperative interactions, whereby complexes of similar orientation associate with each other, dependent on their local concentration. Such *cis* interactions may then result in increased stability and lower turnover. An example of this is the immunological synapse, in which protein-protein interactions cause diffusional trapping and clustering of signaling molecules (Douglass and Vale, 2005). Similarly, in vitro experiments have suggested that multivalent complexes undergo phase transitions, and spontaneously cluster together once their local concentration exceeds a threshold value (Li et al., 2012). In addition, clustering of the core proteins into puncta may be associated with the formation of stable interactions with the cytoskeleton. For example, the stability of E-cadherin clusters at adherens junctions is dependent on the actin cytoskeleton (reviewed in Yap et al., 2015), and cortical actin activity also regulates the mobility of GPI-linked proteins in nanoclusters (Goswami et al., 2008).

Most feedback models describing planar polarity rely on mass action kinetics, whereby proteins interact and exert positive and negative feedback, depending on their relative concentration (e.g., Amonlirdviman et al., 2005, Le Garrec et al., 2006, Burak and Shraiman, 2009, Schamberg et al., 2010). Pk, Dsh, and Dgo have been suggested to be key factors mediating feedback (Amonlirdviman et al., 2005, Jenny et al., 2005, Cho et al., 2015), but we have found that their relative concentrations can vary considerably, without any apparent defects in protein asymmetry (Figure 7E). One possibility is that feedback interactions are slowed down when relative concentrations are changed, but no defect is apparent as the system reaches a steady state. In addition, multiple redundant feedback interactions may exist, and a perturbation in the rate of just one of these will have little overall effect on asymmetry. This would be consistent with our data showing that altering the dosage of one component does not affect asymmetry, but altering stoichiometry more severely (by altering the dosage of several components) is deleterious. Strikingly, however, if the levels of either Fz or Stbm relative to Fmi are lowered, then asymmetry is severely compromised. Therefore, it appears that the core complex cannot undergo normal feedback interactions if the Fz-Fmi:Fmi-Stbm backbone is out of balance (Figure 7F). Interestingly, the exquisite sensitivity of feedback to levels of Fz and Stbm appears to have caused the system to evolve so that their levels are buffered against changes: by altering rates of degradation in the case of Fz or by maintaining excess levels of protein in cellular pools for Stbm.

Overall, our data are consistent with a model in which feedback operates in the context of organization of the core proteins into higher-order signalosome-like structures. We propose that such an organization concentrates components at junctions where they can participate in feedback. This both increases the efficiency of feedback and buffers against fluctuations in protein levels, for example following cell division. Ultimately, this promotes the biphasic partitioning of the core proteins to opposite cell ends.

## Experimental Procedures

Additional information regarding molecular biology, fly stocks, antibodies, imaging, and image analysis is available in Supplemental Experimental Procedures.

### Western Blotting

For pupal wing westerns, 28 hr after puparium formation (APF) pupal wings were dissected directly into sample buffer. One pupal wing equivalent was used per lane. A Bio-Rad ChemiDoc XRS+ was used for imaging, and band intensities from three biological replicates were quantified using ImageJ. Data were compared used unpaired t tests or ANOVA for multiple comparisons.

### Imaging and FRAP Analysis in Pupal Wings

For live imaging, pupae were prepared and imaged at 28 hr APF (unless otherwise stated) as previously described (Strutt et al., 2011). For FRAP, regions of interest (ROIs) of ∼2 μm^2^ were selected for puncta and non-puncta. After imaging, ROIs were manually reselected in ImageJ and quantitated. Control unbleached regions were also quantitated to control for acquisition bleaching. Data were corrected for acquisition bleaching and normalized against an average of the prebleached values and the first postbleach value. Data from ROIs in the same wing were averaged, and Prism (v6 GraphPad) was used to fit a one-phase exponential association curve for each wing. Data from several wings were then used to fit a final exponential association curve, and an extra sum-of-squares F test was performed to compare curve plateaux (Y_max_) between puncta and non-puncta.

To combine the FRAP data with the puncta stoichiometry data, the normalized stoichiometry data were multiplied by the stable and unstable proportions. Standard errors for each experiment were combined, and plotted as the square root of the sum of each error squared. To compare relative stable and unstable amounts in puncta and non-puncta, each dataset was normalized to 2 units of Fmi-EGFP, and two-way ANOVA with Holm-Šídák correction was performed.

To compare stable and unstable amounts between EGFP-tagged protein in a wild-type and *fmi*^*E59*^ heterozygous background, prebleached values were averaged for each wing and multiplied by the stable proportion (1 − Y_max_). Stable amounts were then averaged across wings and compared using unpaired t tests.

### Puncta Detection and Quantitation

Membrane masks were generated using Packing Analyzer (Aigouy et al., 2010), and automated puncta detection was carried out using a MATLAB script (see Supplemental Experimental Procedures and Data S1 for MATLAB scripts). The mean intensity of puncta and non-puncta membranes was determined. For live imaging, background due to autofluorescence was subtracted, and mean puncta intensity was averaged across wings, and compared using unpaired t tests or ANOVA.

For comparing intensity of individual puncta labeled with two different antibodies in the same wing, a puncta mask was generated as above for one channel, and this mask was then used to measure puncta intensity in both channels.

For quantitating puncta in clones in fixed images, wild-type and mutant regions were separated, and puncta detection applied to each region separately. Puncta intensity was compared between control and mutant regions in the same wing using paired t tests.

For measuring puncta asymmetry on the borders of *fmi-EGFP*, *fz-EGFP*, and *stbm-EGFP* twin clones, puncta were detected over the whole image on the basis of Fmi staining. Puncta on proximal and distal cell ends were selected manually in ImageJ, and mean intensity and puncta area were measured for Fmi, Fz, or Stbm. Proximal and distal puncta intensity were averaged per wing and compared in the same wing using paired t tests.

### Polarity Measurement

A MATLAB script was used to determine the angle of maximum asymmetry for each cell (see Supplemental Experimental Procedures and Data S1 for supplemental MATLAB scripts). The vector polarity was then averaged for all cells in the image to give a mean vector polarity (asymmetry ratio on plots). The SD in the cell-by-cell polarity angle was used as a measure of the coordination in polarity between cells. Averaging between wings and statistical tests were similar to those for puncta detection.

## Author Contributions

H.S., J.G., and D.S. designed experiments and wrote MATLAB scripts. H.S. and J.G. conducted experiments and analyzed the data. H.S. and D.S. wrote the paper.

## Supplemental Information

Document S1. Supplemental Experimental Procedures, Figures S1–S7, Tables S1–S4

Data S1. MATLAB Scripts, Related to Experimental Procedures MATLAB scripts for measuring mean puncta intensity, mean intensity on proximo-distal versus lateral membranes, and polarity.

Document S2. Article plus Supplemental Information
