# Supplementary material for: Robust Asymmetric Localization of Planar Polarity Proteins Is Associated with Organization into Signalosome-like Domains of Variable Stoichiometry
Source: Cell Rep. 2016 Dec 6;17(10):2660–71. doi: 10.1016/j.celrep.2016.11.021 (PMC5177602; doi:10.1016/j.celrep.2016.11.021)
Supplement: Document S1. Supplemental Experimental Procedures, Figures S1–S7, Tables S1–S4 [file mmc1.pdf]

Cell Reports, Volume *17*

Supplemental Information

Robust Asymmetric Localization of Planar Polarity Proteins Is Associated with Organization into Signalosome-like Domains of Variable Stoichiometry

Helen Strutt, Jessica Gamage, and David Strutt

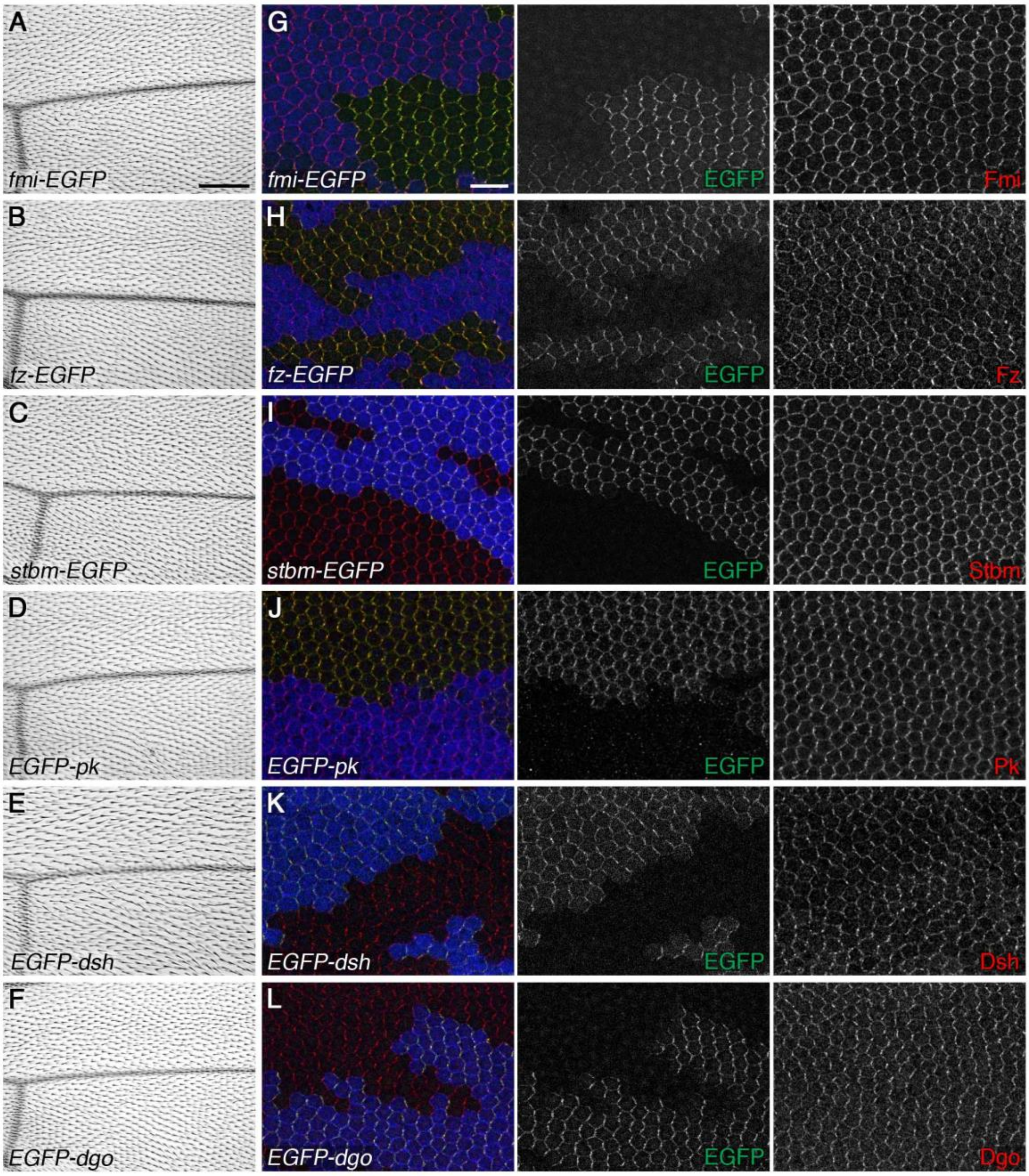

Figure S1. Related to Figure 1. Characterisation of flies expressing EGFP-tagged core proteins.

(A-F) Images of adult wings from flies homozygous for *fmi-EGFP* (A), *fz-EGFP* (B), *P[acman]-stbm-EGFP stbm*^{6} (C), *EGFP-pk* (D), *dsh*^{V26}*; P[acman]-EGFP-dsh* (E) or *P[acman]-EGFP dgo dgo*^{380} (F). Note that the trichomes of EGFP-Dsh are slightly longer than normal, which causes mild mounting artefacts, however polarity is normal. Scale bar is 50 µm.

(G-L) Twin clone experiments, to compare levels of endogenous protein in puncta to levels of tagged protein. (G) *Ubx-FLP; FRT42 fmi-EGFP/FRT42 lacZ*, Fmi staining in red. (H) *Ubx-FLP; fz-EGFP FRT80/arm-lacZ FRT80*, Fz staining in red. (I) *Ubx-FLP; P[acman]-stbm-EGFP arm-lacZ FRT40 stbm*^{6}*/P[acman]-stbm FRT40 stbm*^{6}, Stbm staining in red. (J) *Ubx-FLP; FRT42 EGFP-pk/FRT42 arm-lacZ*, Pk staining in red. (K) *dsh*^{V26}*, Ubx-FLP/dsh*^{V26}*; P[acman]-EGFP-dsh arm-lacZ FRT40/P[acman]-dsh FRT40*, Dsh staining in red. (L) *Ubx-FLP; P[acman]-EGFP-dgo arm-lacZ FRT40 dgo*^{380}*/P[acman]dgo FRT40 dgo*^{380}, Dgo staining in red. Wild-type tissue marked by loss of EGFP (green) and presence of ß-gal staining (blue, G, H, J), or by loss of both EGFP (green) and ß-gal staining (blue, I, K, L). Slightly less Fmi-EGFP localises to junctions than endogenous Fmi (G), whilst more Fz-EGFP than endogenous Fz localises to junctions (H). Slightly less EGFP-Dsh than endogenous Dsh localises to junctions (K), and also less is seen in the cytoplasm. See Table S1 for quantitations. Scale bar is 10 µm.

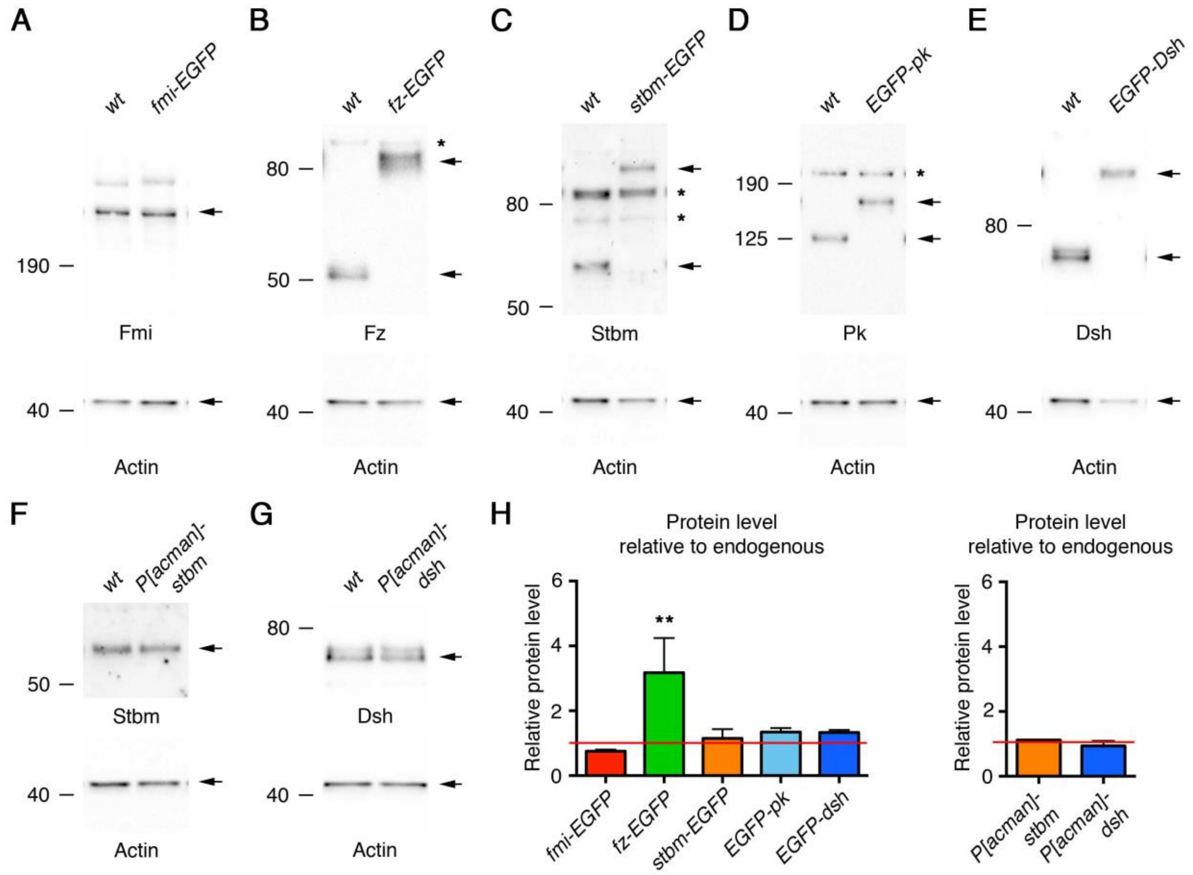

Figure S2. Related to Figure 1. Cellular levels of EGFP-tagged core proteins in pupal wings.

(A-G) Western blots comparing levels of core proteins in pupal wings from wild-type or flies homozygous for *fmi-EGFP* (A), *fz-EGFP* (B), *P[acman]-stbm-EGFP stbm*^{6} (C), *EGFP-pk* (D), *dsh*^{V26}*; P[acman]-EGFP-dsh* (E), *P[acman]-stbm stbm*^{6} (F) or *dsh*^{V26}*; P[acman]-dsh* (G). Arrows indicate specific bands of the expected size, and asterisks mark cross-reacting bands that are still present in samples from null mutants. Addition of EGFP causes an increase in protein size on the gels, except for Fmi-EGFP, where the protein is cleaved into two fragments and an N-terminal antibody is used for detection (Usui et al., 1999, upper band is unprocessed Fmi in panel A).

(H) Quantitation of western blots, from three biological replicates. Protein levels in flies carrying tagged transgenes are shown relative to the amount of endogenous protein in wild-type wings. Error bars are sem, $p<0.01$** (unpaired t-test). Note that *P[acman]-stbm stbm* and *dsh; P[acman]-dsh* wings show identical levels of expression of Stbm and Dsh as seen in wild-type wings, validating their use as a control in twin clone experiments (Fig. S1).

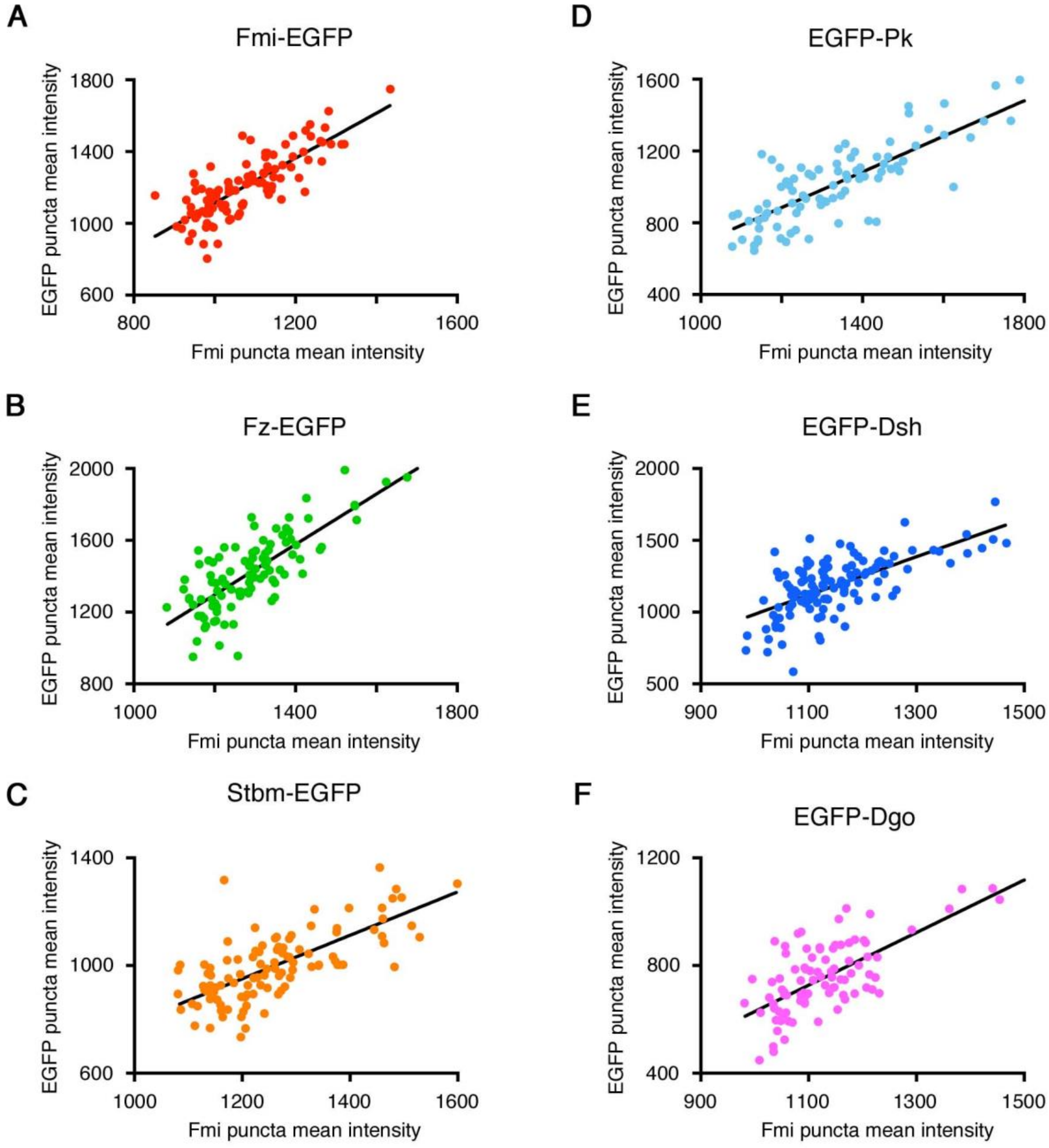

Figure S3. Related to Figure 1. Puncta have a uniform composition.

Pupal wings expressing *fmi-EGFP* (A), *fz-EGFP* (B), *stbm-EGFP* (C), *EGFP-pk* (D), *EGFP-dsh* (E) or *EGFP-dgo* (F) were immunostained for GFP and Fmi. Graphs show data from a single typical image, with intensity of GFP staining in individual puncta plotted against Fmi staining intensity. Units are arbitrary, and different laser intensities were used for each image. For all core proteins, the points fit to a straight line, suggesting that puncta have similar relative levels of each core protein. For *fmi-EGFP* stainings, the GFP and Fmi antibodies recognise the same molecule and R-squared (goodness of fit) is 0.64, suggesting that this is the best fit that can be achieved after taking into account antibody staining noise. For the other stainings shown here, R-squared is 0.61 (*fz-EGFP*), 0.51 (*stbm-EGFP*), 0.66 (*EGFP-pk*), 0.44 (*EGFP-dsh*) and 0.47 (*EGFP-dgo*).

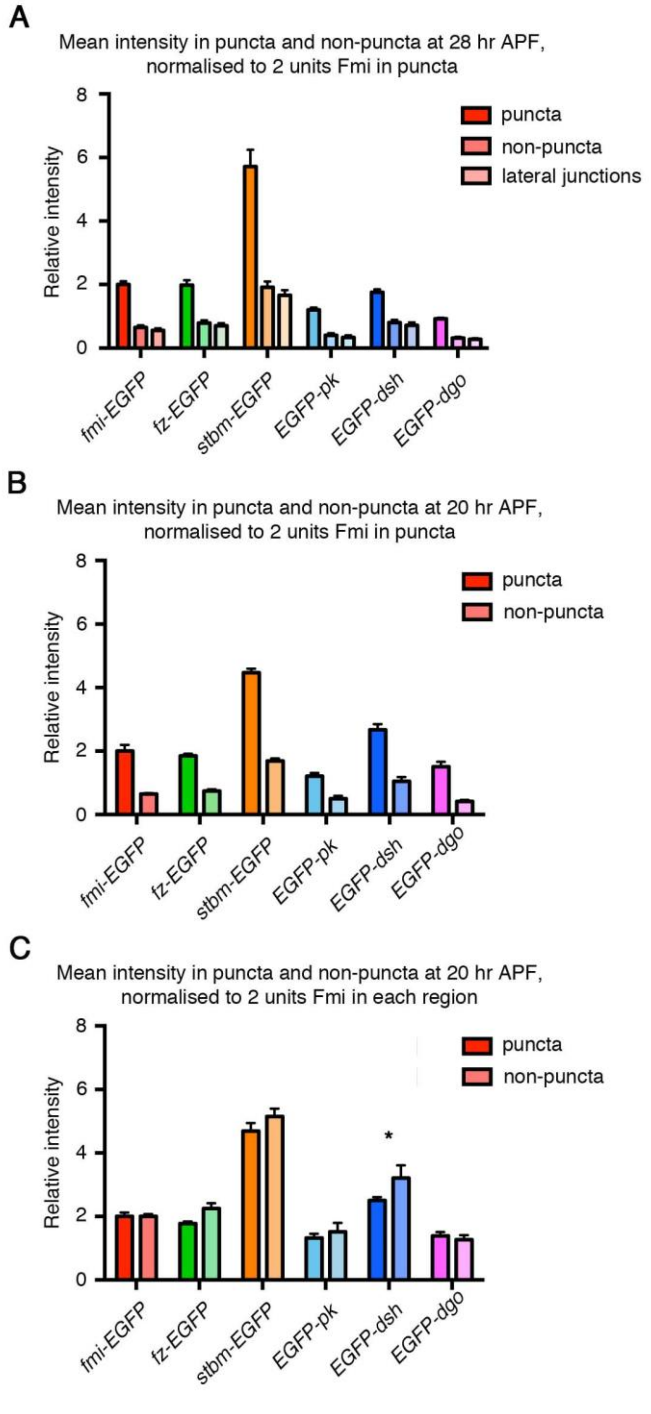

Figure S4. Related to Figure 2. Levels of core proteins in puncta, non-puncta and lateral junctions.

(A) Mean intensity of EGFP fluorescence in puncta, non-puncta and lateral junctions, at 28 hr APF (A) or 20 hr APF (B, C). Data are normalised to 2 units of Fmi-EGFP in puncta (A, B) or to 2 units of Fmi-EGFP in each region (C). (A, B) Puncta are approximately three times as bright as non-puncta at both stages of development. (C) Relative levels of Dsh are significantly increased in non-puncta at 20hr APF. Error bars are sem, $p<0.05$* (2 way ANOVA used to compare puncta and non-puncta). Sample sizes as in Figs. 1E and 2C.

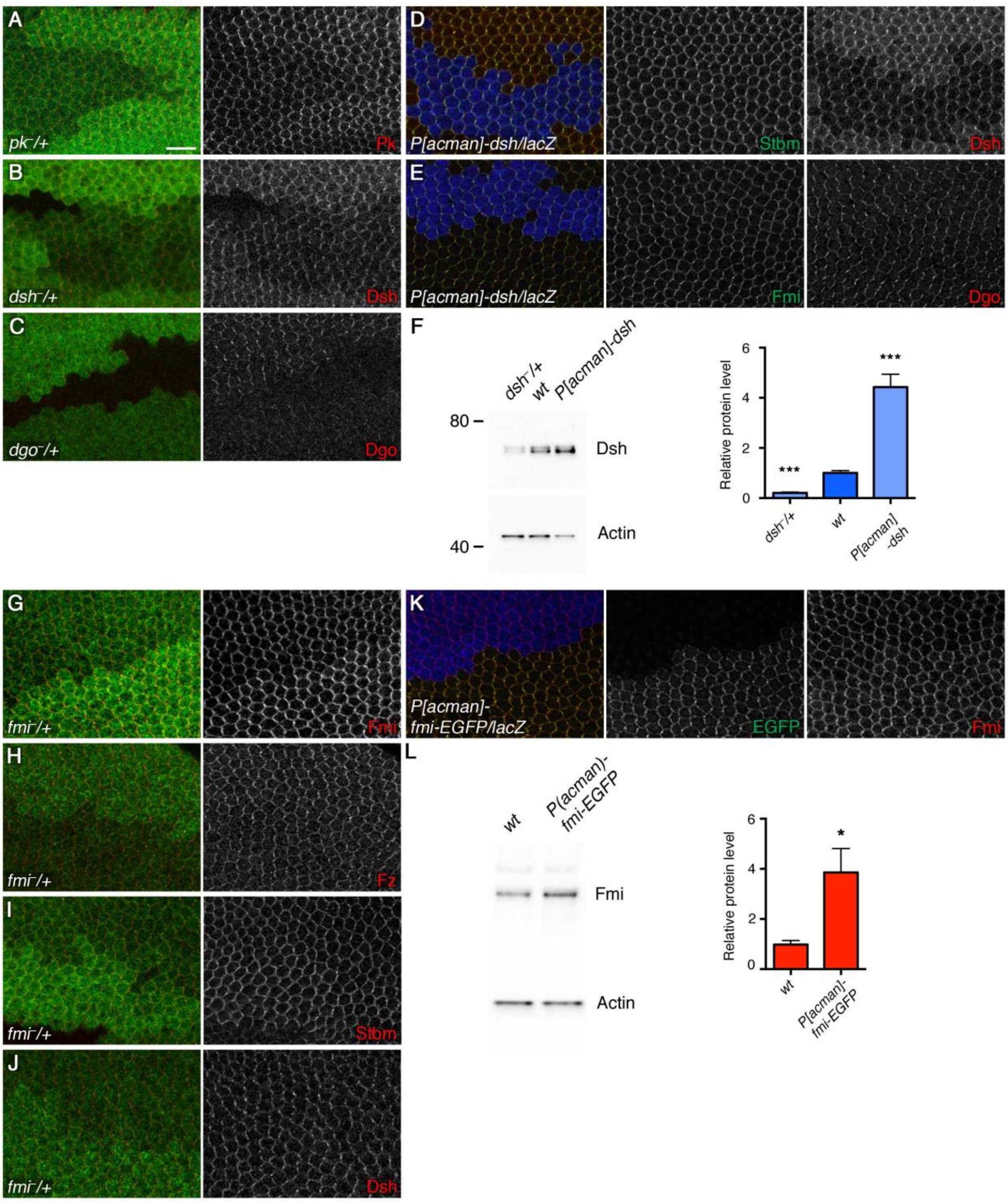

Figure S5. Related to Figures 3 and 4. The effects of decreasing or increasing gene dosage of *prickle*, *dishevelled*, *diego* or *flamingo*.

(A-C) Pupal wings heterozygous for *pk-sple*^{13} (A), *dsh*^{V26} (B) and *dgo*^{380} (C), in which loss of function clones were induced, stained for Pk (red, A), Dsh (red, B) or Dgo (red, C) and ß-gal clonal marker (green). Bright green staining marks twinspot tissue with 2 gene dosages, whilst pale green staining marks heterozygous tissue with 1 gene dosage and absence of green indicates homozygous mutant tissue. In heterozygous tissue, levels of Pk, Dsh and Dgo at junctions are lowered relative to homozygous tissue, indicating that levels of protein in the cell are limiting for incorporation into junctions. Scale bar is 10 µm.

(D, E) Pupal wings with clones of *P[acman]-dsh* (*Ubx-FLP; P[acman]-dsh FRT40/arm-lacZ FRT40*). Tissue staining for the ß-gal clonal marker in blue carries 2 doses of endogenous *dsh* only, whilst tissue lacking the ß-gal clonal marker carries 2 doses of endogenous *dsh* and 2 doses of *P[acman]-dsh*. (D) Wings stained for Stbm

(green) and Dsh (red). (E) Wings stained for Fmi (green) and Dgo (red). Note excess Dsh localising to the cytoplasm, also no increase in Fmi, Stbm or Dgo staining at junctions is seen when 4 doses of *dsh* are present.

(F) Western blots comparing levels of Dsh in pupal wings from dsh^{V26}/+ heterozygous flies, wild-type flies or flies homozygous for *P[acman]-dsh* (carrying two doses of endogenous *dsh* plus 2 doses of *dsh* in the P[acman] construct). Quantitations from three biological replicates show that dsh^{V26}/+ wings have 5-fold less Dsh compared to wild-type ($p<0.05$*), whilst *P[acman]-dsh* wings have 4.4-fold more Dsh ($p<0.001$***, ANOVA), possible due to saturation of degradation pathways.

(G-J) Pupal wings heterozygous for fmi^{E59}, in which loss of function clones were induced, stained for ß-gal clonal marker (green) and Fmi (G, red), Fz (H, red), Stbm (I, red) or Dsh (J, red). Bright green staining marks twinspot tissue with 2 gene dosages, whilst pale green staining marks heterozygous tissue with 1 gene dosage and absence of green indicates homozygous mutant tissue. In tissue heterozygous for *fmi*, levels of Fmi and Fz at junctions are lowered, relative to homozygous tissue (G, H). A slight decrease in levels of Stbm is also detectable in tissue heterozygous for *fmi* (I), but no decrease in Dsh is apparent (J).

(K) Pupal wing with clone of *P[acman]-fmi-EGFP* in a wild-type background (*Ubx-FLP; P[acman]-fmi-EGFP FRT40/arm-lacZ FRT40*). Tissue staining for the ß-gal clonal marker (blue) carries 2 doses of endogenous *fmi* only, whilst tissue lacking the ß-gal clonal marker but expressing EGFP (green) carries 2 doses of endogenous *fmi* and 2 doses of *P[acman]-fmi-EGFP*. Wings also stained for Fmi (red). There is only a marginal increase in levels of Fmi at junctions when 4 doses of *fmi* are present.

(L) Western blot comparing levels of Fmi in pupal wings from wild-type flies or flies homozygous for *P[acman]-fmi-EGFP* (carrying two doses of endogenous *fmi* plus 2 doses of *fmi-EGFP* in the P[acman] construct). Quantitations from three biological replicates show a 3.9-fold increase in cellular levels of Fmi in *P[acman]-fmi-EGFP* wings ($p<0.05$*, unpaired t-test).

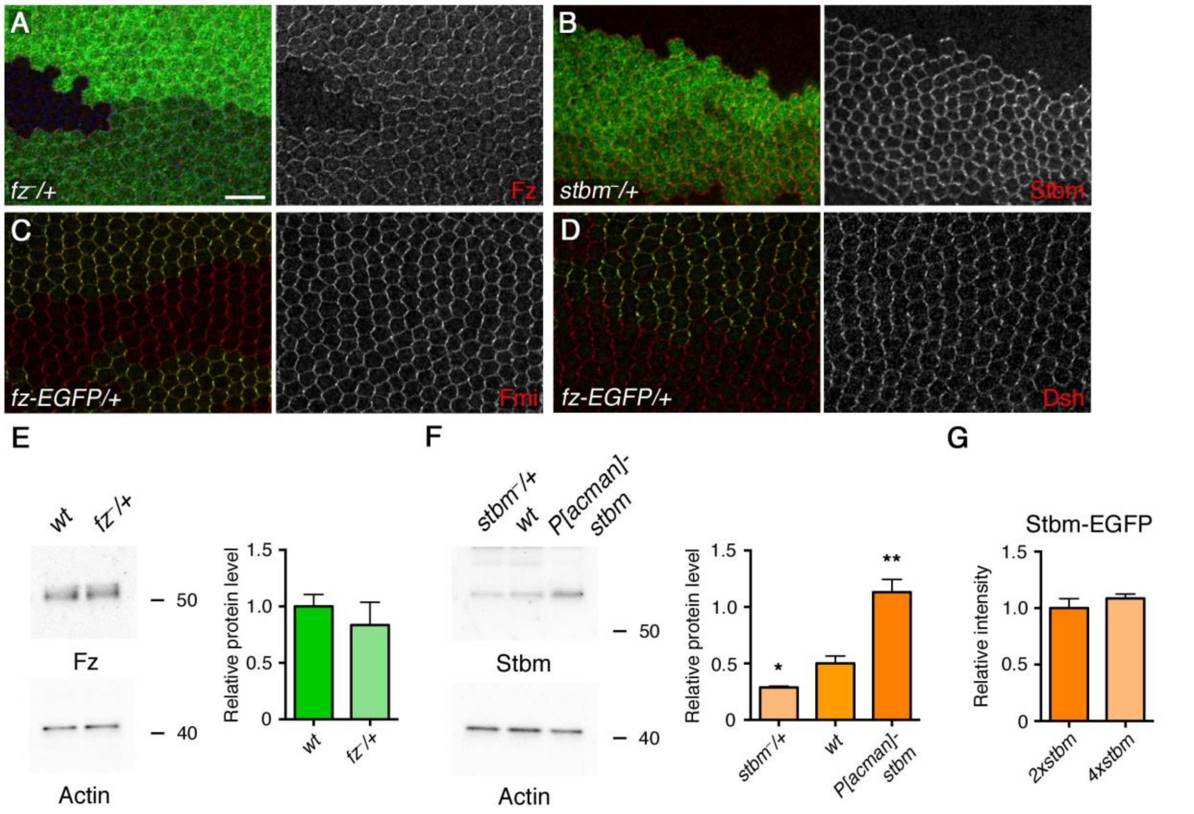

Figure S6. Related to Figure 5. Decreasing *frizzled* and *strabismus* gene dosage.

(A, B) Pupal wings heterozygous for *fz*^{P21} (A) or *stbm*^{6} (B), in which loss of function clones were induced, stained for Fz (red, A) or Stbm (red, B) and ß-gal clonal marker (green). Bright green staining marks twinspot tissue with 2 gene dosages, whilst pale green staining marks heterozygous tissue with 1 gene dosage and absence of green indicates homozygous mutant tissue. Halving gene dosage for either *fz* or *stbm* does not cause a reduction in levels of protein in puncta, suggesting that the amount of protein within the cell is not limiting. Note that the amount of Fz in the cytoplasm decreases slightly in *fz* heterozygous tissue. The swirling in Fz localisation is probably due to non-autonomous effects of the neighbouring *fz* mutant tissue. Scale bar is 10 µm.

(C, D) Pupal wings carrying clones of *fz-EGFP*, as in Fig S1H. Tissue expressing Fz-EGFP is marked by green junctional staining, whilst tissue lacking green staining expresses endogenous *fz*. Wings stained for Fmi (C) or Dsh (D) in red. More Fz-EGFP localises to junctions than endogenous Fz (see Fig S1H), but Fmi and Dsh levels do not change.

(E, F) Western blots and quantitations, comparing levels of Fz in pupal wings from *fz*^{P21}/+ heterozygous flies to wild-type flies (E) or levels of Stbm in pupal wings from *stbm*^{6}/+ heterozygous flies to wild-type flies or flies homozygous for *P[acman]-stbm* (F). Quantitations are from three biological replicates, p<0.01**, p<0.05* (unpaired t-test (E) or ANOVA (F)).

(G) Relative mean intensity of puncta in live pupal wings, comparing flies carrying 1 dose endogenous gene and 1 dose tagged gene with those carrying 2 doses endogenous gene and 2 doses tagged gene. Puncta intensity does not increase when *stbm* dosage is doubled. Error bars are sem, data compared by unpaired t-test, n = 15 (*P[acman-stbm-EGFP/stbm*^{6}) , n = 14 (*P[acman]-stbm-EGFP*).

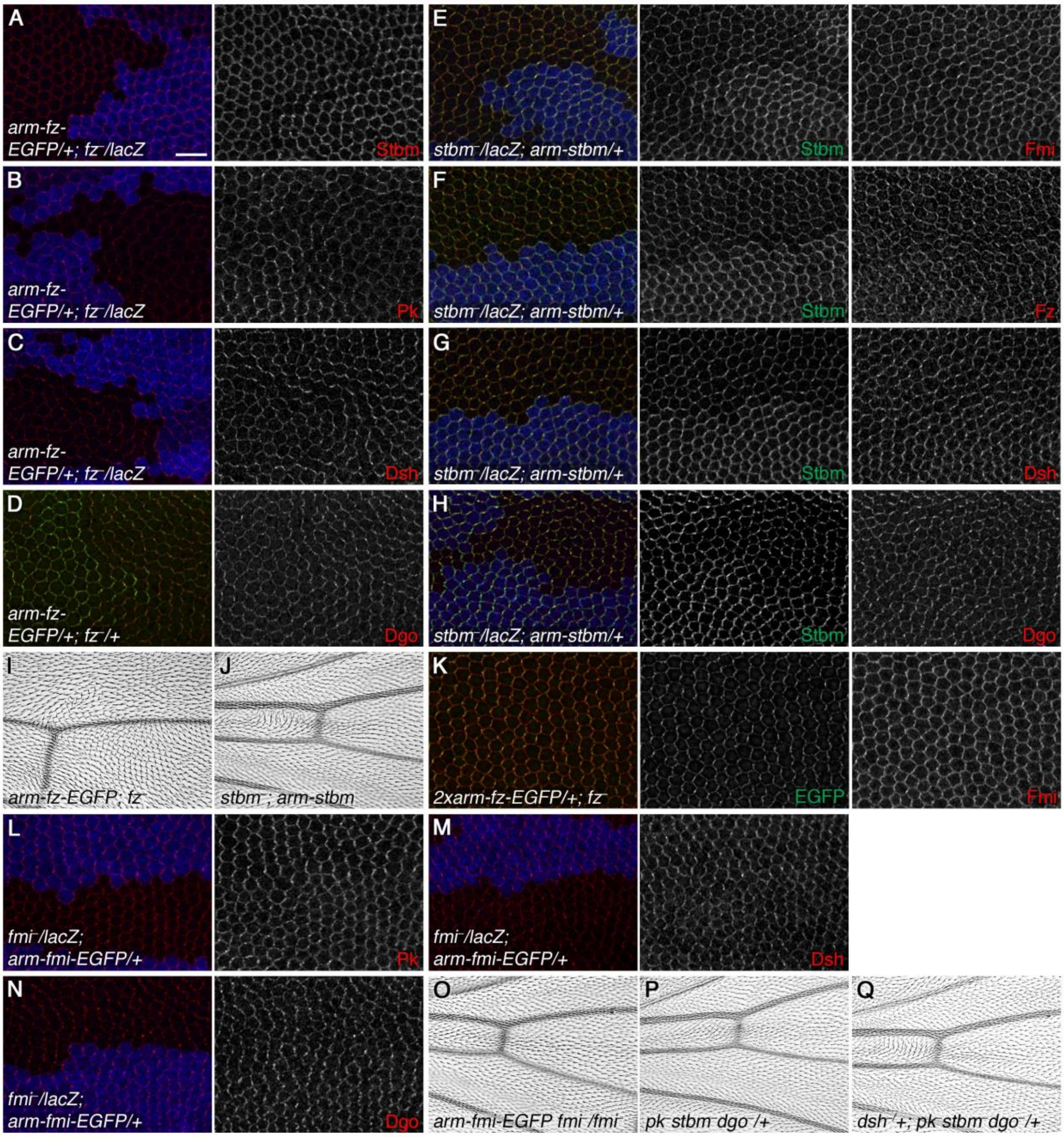

Figure S7. Related to Figures 5 and 6. Wings expressing *arm-frizzled-EGFP, arm-strabismus and arm-flamingo-EGFP*.

(A-D) Pupal wings carrying one copy of *arm-fz-EGFP*, with fz^{P21} clones marked by loss of ß-gal staining (blue, A-C), or by upregulation of EGFP fluorescence (green, D). Wings are labelled for Stbm (A, red), Pk (B, red), Dsh (C, red) or Dgo (D, red). Asymmetric localisation of core proteins is lost in regions with *fz* clones. Levels of Stbm, Pk and Dsh at junctions decrease slightly, whilst levels of Dgo do not decrease. Scale bar is 10 µm.

(E-H) Pupal wings carrying one copy of *arm-stbm*, with $stbm^{6}$ clones marked by loss of ß-gal staining (blue). Wings stained for Stbm (green), and for Fmi (E, red), Fz (F, red), Dsh (G, red) or Dgo (H, red). Junctional levels of Fmi, Fz, Dsh and Dgo do not appear to alter in regions with *stbm* clones. Asymmetry is reduced.

(I, J) Adult wings of *arm-fz-EGFP/+;* fz^{P21}, imaged on the ventral surface around the posterior cross vein (I) and $stbm^{6}$*; arm-stbm/+* imaged proximally on the dorsal wing surface (J). Trichome orientation defects are only seen in proximal regions of $stbm^{6}$*; arm-stbm* wings; this region appears most sensitive to reduction in Stbm levels.

(K) Pupal wings carrying 2 copies of *arm-fz-EGFP* in a fz^{P21} background, showing EGFP fluorescence (green) and stained for Fmi (red). The asymmetry ratio and standard deviation in the cell-by-cell polarity angle were not significantly different to those of wild-type wings (n = 8), in contrast to wings carrying a single copy of *arm-fz-EGFP* (see Fig 5E).

(L-N) Pupal wings carrying one copy of *arm-fmi-EGFP*, with *fmi*^{E59} clones marked by loss of ß-gal staining (blue). Wings labelled for Pk (L, red), Dsh (M, red) or Dgo (N, red). Junctional levels of Pk and Dgo only decrease very slightly in regions with *fmi* clones, whilst Dsh levels appear unchanged.

(O-Q) Adult wings of *arm-fmi-EGFP fmi*^{E59}*/fmi*^{E45} (O), *pk-sple*^{13}, *stbm*^{6}, *dgo*^{380}/+ (P) or dsh^{V26}/+; *pk-sple*^{13} *stbm*^{6} *dgo*^{380}/+ (Q), all imaged proximally on the dorsal wing surface. No trichome orientation defects are seen in *arm-fmi-EGFP fmi*^{E59}*/fmi*^{E45} wings, and only mild defects are seen in proximal regions of *pk-sple*^{13}, *stbm*^{6}, *dgo*^{380}/+ and dsh^{V26}/+; *pk-sple*^{13} *stbm*^{6} *dgo*^{380}/+ wings.

	Fmi-EGFP	Fz-EGFP	Stbm-EGFP	EGFP-Pk	EGFP-Dsh	EGFP-Dgo
Mean puncta intensity compared to untagged protein	0.88<br>sem 0.02<br>***	1.58<br>sem 0.07<br>***	0.99<br>sem 0.03<br>ns	1.16<br>sem 0.02<br>***	0.84<br>sem 0.03<br>***	1.01<br>sem 0.03<br>ns
Mean puncta intensity in X-EGFP/+ compared to X-EGFP/X-EGFP	0.51<br>sem 0.05<br>ns	0.47<br>sem 0.04<br>ns	0.42<br>sem 0.02<br>**	0.49<br>sem 0.02<br>ns	0.51<br>sem 0.04<br>ns	0.60<br>sem 0.04<br>*

Table S1. Related to Figure 1. Quantitation of protein levels in puncta.

Top: Mean intensity of antibody staining in puncta in pupal wings, comparing tissue that is homozygous for tagged genes, to twin clone tissue that is homozygous for endogenous or untagged genes. Genotypes as in Fig S1G-L. Background subtracted was from null clones stained in parallel. The intensities of Fmi-EGFP, EGFP-Pk and EGFP-Dsh puncta are significantly different to those of control puncta, but the difference is less than 20%. $p<0.001$*** (paired t-test), ns = not significant. n = 16 (Fmi staining of *fmi-EGFP* clones), n = 18 (Fz staining of *fz-EGFP* clones), n = 24 (Stbm staining of *stbm-EGFP* clones), n = 29 (Pk staining of *EGFP-pk* clones), n = 16 (Dsh staining of *EGFP-dsh* clones), n = 13 (Dgo staining of *EGFP-dgo* clones).
Bottom: Relative mean intensity of puncta, comparing live images of wings carrying 1 copy of EGFP-tagged protein and 1 copy of endogenous protein, to wings carrying 2 copies of indicated EGFP-tagged core protein. Note that if EGFP/+ puncta were more than half as bright as the EGFP/EGFP puncta, this might suggest that the EGFP-tagged protein out-competes the wild-type one for inclusion into puncta. Conversely, if the EGFP/+ puncta were less than half as bright as the EGFP/EGFP puncta this might suggest the opposite. Asterisks indicate significant differences from the expected result that intensity of puncta in wings expressing 1 copy of EGFP-tagged protein should be 50% that of puncta in wings expressing 2 copies of EGFP-tagged protein, $p<0.05$*, $p<0.01$** (1 sample t-test), ns = not significant. For homozygous EGFP-tagged proteins, sample sizes are as in Fig.1E. n = 12 (*fmi-EGFP/+*), n = 10 (*fz-EGFP/+*), n = 11 (*P[acman]-stbm-EGFP* $stbm^{6}$/+), n = 11 (*EGFP-pk/+*), n = 8 (dsh^{V26}/+; *P[acman]-EGFP-dsh/+*), n = 13 (*P[acman]-EGFP-dgo* dgo^{380}/+).

	**Asymmetry ratio**		**SD of polarity angle (degrees)**	
	wt	X-EGFP	wt	X-EGFP
**Fmi-EGFP**	1.39<br>sem 0.04	1.51<br>sem 0.04<br>*	20.87<br>sem 1.25	20.76<br>sem 1.34<br>ns
**Fz-EGFP**	1.45<br>sem 0.03	1.38<br>sem 0.03<br>*	15.13<br>sem 1.55	18.14<br>sem 1.20<br>ns
**Stbm-EGFP**	1.52<br>sem 0.04	1.39<br>sem 0.03<br>**	15.12<br>sem 1.07	18.52<br>sem 1.08<br>*
**EGFP-Pk**	1.42<br>sem 0.02	1.50<br>sem 0.03<br>**	18.57<br>sem 0.56	16.74<br>sem 0.94<br>ns
**EGFP-Dsh**	1.60<br>sem 0.05	1.62<br>sem 0.06<br>ns	21.36<br>sem 1.33	19.72<br>sem 1.25<br>ns
**EGFP-Dgo**	1.47<br>sem 0.02	1.44<br>sem 0.04<br>ns	16.75<br>sem 0.77	18.78<br>sem 1.20<br>ns

Table S2. Related to Figure 1. Quantitation of asymmetry of EGFP-tagged core proteins.

Asymmetry ratio (left) and standard deviation of polarity angle (right), comparing Fmi staining in tissue that is homozygous for tagged genes, to twin clone tissue that is homozygous for endogenous or untagged genes (wt). Genotypes as in Fig S1G-L. $p<0.01$**, $p<0.05$* (paired t-test), ns = not significant. n = 10 (*fmi-EGFP* clones), n = 10 (*fz-EGFP* clones), n = 10 (*stbm-EGFP* clones), n = 12 (*EGFP-pk* clones), n = 8 (*EGFP-dsh* clones), n = 9 (*EGFP-dgo* clones).

		Fmi-EGFP	Fz-EGFP	Stbm-EGFP	EGFP-Pk	EGFP-Dsh	EGFP-Dgo	Asymmetry
***fmi*$^-$/+**	puncta	**0.59**<br>+/- 0.10	0.64<br>+/- 0.07	0.94<br>+/- 0.15	1.06<br>+/- 0.14	0.91<br>+/- 0.08	1.09<br>+/- 0.15	~wt
	non-puncta	**0.49**<br>+/- 0.09	0.65<br>+/- 0.07	0.87<br>+/- 0.13	1.08<br>+/- 0.12	0.92<br>+/- 0.08	1.03<br>+/- 0.18	
***pk-sple*$^-$/+**	puncta	1.02<br>+/- 0.12	0.97<br>+/- 0.06	1.24<br>+/- 0.25	**0.53**<br>+/- 0.10	0.96<br>+/- 0.07	1.06<br>+/- 0.13	~wt
	non-puncta	1.08<br>+/- 0.13	1.04<br>+/- 0.13	1.30<br>+/- 0.28	**0.49**<br>+/- 0.10	1.02<br>+/- 0.07	1.08<br>+/- 0.14	
***dsh*$^-$/**	puncta	0.81<br>+/- 0.04	0.91<br>+/- 0.10	1.02<br>+/- 0.19	0.94<br>+/- 0.06	**0.51**<br>+/- 0.04	1.03<br>+/- 0.10	~wt
	non-puncta	0.85<br>+/- 0.05	1.00<br>+/- 0.10	1.01<br>+/- 0.20	1.00<br>+/- 0.07	**0.44**<br>+/- 0.03	1.01<br>+/- 0.10	
***dgo*$^-$/+**	puncta	0.81<br>+/- 0.10	0.87<br>+/- 0.09	0.93<br>+/- 0.15	1.09<br>+/- 0.14	0.84<br>+/- 0.06	**0.41**<br>+/- 0.04	~wt
	non-puncta	0.83<br>+/- 0.10	0.87<br>+/- 0.10	0.90<br>+/- 0.13	1.11<br>+/- 0.14	0.82<br>+/- 0.05	**0.39**<br>+/- 0.06	
**4 x *fmi***	puncta	**1.19**<br>+/- 0.10	-	-	-	-	-	-
	non-puncta	**1.21**<br>+/- 0.10	-	-	-	-	-	
**4 x *stbm***	puncta	-	-	**1.09**<br>+/- 0.08	-	-	-	-
	non-puncta	-	-	**1.18**<br>+/- 0.11	-	-	-	
**4 x *dsh***	puncta	1.07<br>+/- 0.09	1.05<br>+/- 0.09	-	0.94<br>+/- 0.08	**1.43**<br>+/- 0.16	-	~wt
	non-puncta	1.08<br>+/- 0.10	0.98<br>+/- 0.08	-	0.90<br>+/- 0.06	**1.59**<br>+/- 0.16	-	
**4 x *dgo***	puncta	1.12<br>+/- 0.08	0.91<br>+/- 0.06	-	0.95<br>+/- 0.08	-	**3.03**<br>+/- 0.80	~wt

Table S3. Related to Figures 3 and 4. Summary of stoichiometry data.

Quantitation of puncta and non-puncta intensity in live images of pupal wings from flies expressing EGFP tagged transgenes, relative to controls. Errors are 95% confidence intervals. Bold indicates where the dosage of the tagged gene itself is altered.

	Fmi	Fz	Stbm	Pk	Dsh	Dgo	Asymmetry
arm-fmi-EGFP	**0.59** +/- 0.02	0.85 +/- 0.13	0.69 +/- 0.03	0.94 +/- 0.06	0.98 +/- 0.09	0.91 +/- 0.03	small decrease
arm-fz-EGFP	1.08 +/- 0.11	**0.90** +/- 0.03	0.89 +/- 0.03	0.87 +/- 0.07	0.92 +/- 0.04	0.97 +/- 0.12	strong decrease
arm-stbm	0.97 +/- 0.09	0.99 +/- 0.10	**0.67** +/- 0.02	1.24 +/- 0.10	0.93 +/- 0.42	0.89 +/- 0.06	moderate decrease

Table S4. Related to Figures 5 and 6. Quantitation of immunostaining data for *arm-flamingo-EGFP, arm-frizzled-EGFP* and *arm-strabismus*.

Quantitation of mean membrane intensity of immunostained pupal wings carrying one copy of *arm-fmi-EGFP*, with fmi^{E59} clones (top), one copy of *arm-fz-EGFP*, with fz^{P21} clones (middle), or one copy of *arm-stbm*, with $stbm^{6}$ clones (bottom). Quantitations compare tissue containing mutant clones (only expressing the *arm* transgene) to twin spot tissue without clones (carrying two doses of endogenous gene and one copy of the transgene). Bold indicates where the dosage of the tagged gene itself is altered. Errors are 95% confidence intervals. It was not possible to accurately measure any contribution of antibody staining background. Therefore no background subtraction was used, and the difference in antibody staining between the two regions is likely to be an underestimate. Also note that the antibody staining may not be linear, and the degree of linearity might be different for different antibodies. Fmi antibody staining is relatively lower in tissue expressing the *arm-fmi-EGFP transgene* (0.59 +/- 0.02 compared to wild type tissue) than in *fmi* heterozygous tissue (0.81 +/- 0.08 compared to wild type tissue). The asymmetry ratio decreases strongly for *arm-fz-EGFP* (1.26 in wild-type compared to 0.92 in *fz* mutant tissue), whilst the decrease is less for *arm-stbm* (1.35 in wild-type compared to 1.17 in *stbm* mutant tissue), and there is only a small decrease for *arm-fmi-EGFP* (1.21 for wild-type compared to 1.12 in *fmi* mutant tissue). The asymmetry ratio in the wild-type conditions varies due to differences in antibody staining background (see Supplemental Experimental Procedures).

Supplemental zip file. Related to Experimental Procedures. MATLAB scripts.

MATLAB scripts for measuring mean puncta intensity, mean intensity on proximo-distal versus lateral membranes, and polarity.

Supplemental Experimental Procedures

Molecular biology

For homologous recombination, the sequence for EGFP was inserted into the pRK2 targeting vector (Huang et al., 2008), such that EGFP is upstream of the *LoxP-white-LoxP* cassette. Homology arms of at least 3 kb of genomic DNA for *fz* or *pk* were inserted on either side, such that EGFP is in frame with the open reading frame.

The final exon of *fmi* is spliced into different reading frames for the two isoforms (Chae et al., 1999; Usui et al., 1999). The 3579 amino acid isoform (stan-PA, originally identified by Chae et al., 1999) was shown to be the most abundant in wing discs (Wasserscheid et al., 2007). For homologous recombination, the left homology arm was designed such that the final 10 amino acid exon of this isoform was placed directly downstream of the third (common) exon, and in frame with EGFP, within pRK2. The polyA sequence from SV40 was placed between EGFP and the *LoxP-white-LoxP* cassette, and the right homology arm was from just downstream of exon 3. The splice site needed to make the second isoform (stan-PB) was destroyed by changing the codon usage.

P[acman] constructs (BACPAC resources) were recombineered using standard methods. For N-terminal fusions plasmid PL452-N-EGFP (Addgene) was used as a source vector, whilst for C-terminal fusions a modified version of PL452-C-EGFP (Addgene) was generated, in which the *LoxP*-flanked kanamycin selection cassette site was placed downstream of EGFP rather than upstream. Gene-specific primers were used to amplify EGFP and the selection cassette, in frame with the open reading frame, and the resulting fragment was then recombineered into the relevant P[acman] construct. The kanamycin cassette was then excised, leaving a single LoxP site between *dsh* or *dgo* and the EGFP tag, or downstream of the EGFP tag for *stbm*. The design for *P[acman]-fmi-EGFP* was similar to that of the homologous recombination construct, with the final coding exon of stan-PA in frame with EGFP, followed by the polyA sequence from SV40.

Fly stocks and genetics

Fly stocks are described in FlyBase. FlyBase IDs are *FlyBase: FBgn0024836* (*fmi/starry night*), *FlyBase: FBgn0001085* (*fz*), *FlyBase: FBgn0015838* (*stbm/Vang Gogh*), *FlyBase: FBgn0003090* (*pk*), *FlyBase: FBgn0000499* (*dsh*) and *FlyBase: FBgn0086898* (*dgo*). fmi^{E59}, fz^{P21}, $stbm^{6}$, $pk\text{-}sple^{13}$, dsh^{V26} and dgo^{380} are null alleles. *P[acman]* constructs were integrated into the genome via ΦC31-mediated recombination into the *attP40* landing site. *P[acman]-stbm-EGFP* and *P[acman]-EGFP-dgo* were then recombined with $stbm^{6}$ or dgo^{380}, respectively, whilst *P[acman]-EGFP-dsh* was crossed into a dsh^{V26} mutant background. To avoid dosage compensation effects, females of *P[acman]-EGFP-dsh* were used for imaging.

For homologous recombination, the targeting vectors were introduced into the genome by P-element mediated transgenesis, to produce donor strains. Homologous recombination was carried out as described by Huang et al (2008). Targeted lines on the correct chromosome were verified by PCR of EGFP, and the *white* marker gene was excised by Cre-Lox recombination, leaving a single *LoxP* site downstream of EGFP.

Transgenes used were *armP-fz-EGFP* (Strutt, 2001) and *armP-fmi-EGFP* (Strutt et al., 2011). *armP-stbm* is full-length Stbm coding sequence under control of the *arm* promoter. Mitotic clones were induced using the FLP/FRT system and *Ubx-FLP*, except for when heterozygous tissue was needed, in which case *hs-FLP* was used.

Transgenics were generated by Genetivision and Bestgene.

Full genotypes for images are:

Figure 1

(E) *fmi-EGFP – fz-EGFP – P[acman]-stbm-EGFP* $stbm^{6}$ *– EGFP-pk –* dsh^{V26}*; P[acman]-EGFP-dsh – P[acman]-EGFP-dgo* dgo^{380}

(G) *Ubx-FLP; fz-EGFP FRT80/FRT80*

(H) *Ubx-FLP; P[acman]-stbm-EGFP FRT40* $stbm^{6}$*/P[acman]-stbm FRT40* $stbm^{6}$

(I) *Ubx-FLP; FRT42 fmi-EGFP/FRT42*

Figure 2

(A,C,D) As Fig 1(E)

(B) *fmi-EGFP/+ – fz-EGFP/+ – P[acman]-stbm-EGFP* $stbm^{6}$*/+ – EGFP-pk/+ –* dsh^{V26}*/+; P[acman]-EGFP-dsh/+ – P[acman]-EGFP-dgo* dgo^{380}*/+*

Figure 3

(A) *EGFP-pk – EGFP-pk/pk-sple*^{13}

(B) *dsh*^{V26}*; P[acman]-EGFP-dsh – dsh*^{V26}*; P[acman}-EGFP-dsh/+*

(C) *P[acman]-EGFP-dgo dgo*^{380} *– P[acman]-EGFP-dgo dgo*^{380}*/dgo*^{380}

(D,O) *fmi-EGFP/+ – fmi-EGFP/pk-sple*^{13} *– dsh*^{V26}*/+; fmi-EGFP/+ – fmi-EGFP/dgo*^{380}

(E) *fz-EGFP/+ – pk-sple*^{13}*/+; fz-EGFP/+ – dsh*^{V26}*/+; fz-EGFP/+ – dgo*^{380}*/+; fz-EGFP/+*

(F) *P[acman]-stbm-EGFP stbm*^{6}*/+ – P[acman]-stbm-EGFP stbm*^{6}*/pk-sple*^{13} *– dsh*^{V26}*/+; P[acman]-stbm-EGFP stbm*^{6}*/+ – P[acman]-stbm-EGFP stbm*^{6}*/dgo*^{380}

(G) *EGFP-pk/+ – dsh*^{V26}*/+; EGFP-pk/+ – EGFP-pk/dgo*^{380}

(H) *dsh*^{V26}*/+; P[acman]-EGFP-dsh/+ – dsh*^{V26}*/+ ; P[acman]-EGFP-dsh/pk-sple*^{13} *– dsh*^{V26}*/+ ; P[acman]-EGFP-dsh/dgo*^{380}

(I) *P[acman]-EGFP-dgo dgo*^{380}*/+ – P[acman]-EGFP-dgo dgo*^{380}*/pk-sple*^{13} *– dsh*^{V26}*/+; P[acman]-EGFP-dgo dgo*^{380}*/+*

(J) *dsh*^{V26}*/+; P[acman]-EGFP-dsh/+ – P[acman]-EGFP-dsh*

(K) *P[acman]-EGFP-dgo dgo*^{380}*/+ – P[acman]-EGFP-dgo*

(L,P) *fmi-EGFP/+ – P[acman]-dsh fmi-EGFP/P[acman]-dsh – P[acman]-dgo fmi-EGFP/P[acman]-dgo*

(M) *fz-EGFP/+ – P[acman]-dsh; fz-EGFP/+ – P[acman]-dgo; fz-EGFP/+*

(N) *EGFP-pk/+ – P[acman]-dsh EGFP-pk/P[acman]-dsh – P[acman]-dgo EGFP-pk/P[acman]-dgo*

Figure 4

(A,H) *fmi-EGFP – fmi-EGFP/fmi*^{E59}

(B,I) *fz-EGFP/+ – fmi*^{E59}*/+; fz-EGFP/+*

(C,G,J) *P[acman]-stbm-EGFP stbm*^{6}*/+ – P[acman]-stbm-EGFP stbm*^{6}*/fmi*^{E59}

(D,K) *EGFP-pk/+ – EGFP-pk/fmi*^{E59}

(E,L) *dsh*^{V26}*/+; P[acman]-EGFP-dsh/+ – dsh*^{V26}*/+; P[acman]-EGFP-dsh/fmi*^{E59}

(F,M) *P[acman]-EGFP-dgo dgo*^{380}*/+ – P[acman]-EGFP-dgo dgo*^{380}*/fmi*^{E59}

Figure 5

(A,B,E) *arm-fz-EGFP/+; fz*^{P21} *FRT80/arm-lacZ FRT80*

(C,D,F) *FRT42 stbm*^{6}*/FRT42 arm-lacZ; arm-stbm/+*

Figure 6

(A-D) *FRT42 fmi*^{E59}*/FRT42 arm-lacZ; arm-fmi-EGFP/+*

(E,F) *fmi-EGFP/+ – fmi-EGFP/pk-sple*^{13} *stbm*^{6} *dgo*^{380} *– dsh*^{V26}*/+; fmi-EGFP/pk-sple*^{13} *stbm*^{6} *dgo*^{380}

Immunostaining and antibodies

Pupal wings were dissected at 28 hr after puparium formation (APF) at 25°C as previously described (Strutt, 2001). Primary antibodies for immunostaining were mouse monoclonal anti-Fmi 74 (DSHB, Usui et al., 1999), affinity-purified rabbit anti-Fz (Bastock and Strutt, 2007), rabbit anti-Stbm (Warrington et al., 2013), rat anti-Stbm (Strutt and Strutt, 2008), affinity-purified rat anti-Pk (Strutt et al., 2013), rat anti-Dsh (Strutt et al., 2006), mouse monoclonal anti-βgal (Promega), rabbit anti-βgal (Cappel) and rabbit anti-GFP (Abcam, affinity-purified). Anti-Dgo is a rat antibody directed against amino acids 671-927, and affinity purified with a fusion protein against amino acids 671-780.

Westerns were probed with mouse monoclonal anti-Fmi 74 (DSHB, Usui et al., 1999), affinity-purified rabbit anti-Fz (Bastock and Strutt, 2007), rabbit anti-Stbm (Rawls and Wolff, 2003), affinity-purified rat anti-Pk (Strutt et al., 2013), affinity-purified rabbit anti-Dsh (Strutt et al., 2006) and Actin AC-40 mouse monoclonal (Sigma). We do not have an anti-Dgo antibody suitable for western blotting.

Imaging

Both live and fixed pupal wings were imaged on a Nikon A1R GaAsP confocal microscope using a 60x NA1.4 apochromatic lens, giving a pixel size of 70 nm, and the pinhole was set to 1.2 AU. For fixed samples, 9 Z-slices separated by 150 nm were imaged, and then the 3 brightest slices around puncta were selected and averaged for each channel in ImageJ. For live imaging, a single slice was taken, and the laser power at the lens was monitored to ensure that samples were imaged at constant laser power. Imaging of EGFP solution demonstrated that detection varied linearly with concentration over a wide range of intensities.

For FRAP analysis, images were 256 x 256 pixels, with a pixel size of 100 nm, and a pinhole of 1.2 AU. ROIs of about 2 μm^2 were selected for puncta and non-puncta. Three pre-bleach images were taken, and ROIs were then bleached using a 488 nm Argon laser at 100% with 8 passes (1 sec total time). Immediately following bleaching, 5 images were taken at 5 sec intervals, followed by 10 images at 10 sec intervals, 10 images at 15 sec intervals and 8 images at 30 sec intervals.

Puncta detection and quantitation

For puncta detection on live images, out of focus regions were cropped out and a membrane mask was generated using Packing Analyzer (Aigouy et al., 2010). The mask from Packing Analyzer was dilated and eroded to smooth its edges, before dilating to a final radius of 8 pixels (560 nm). Puncta detection and quantitation was carried out using a MATLAB script (Mathworks). To improve puncta detection, the intensity histogram of the image was stretched so that the top 0.1% and bottom 0.1% of data were saturated. Noise was removed by applying a Gaussian filter followed by a median filter (Supplemental Experimental Procedures Fig 1).

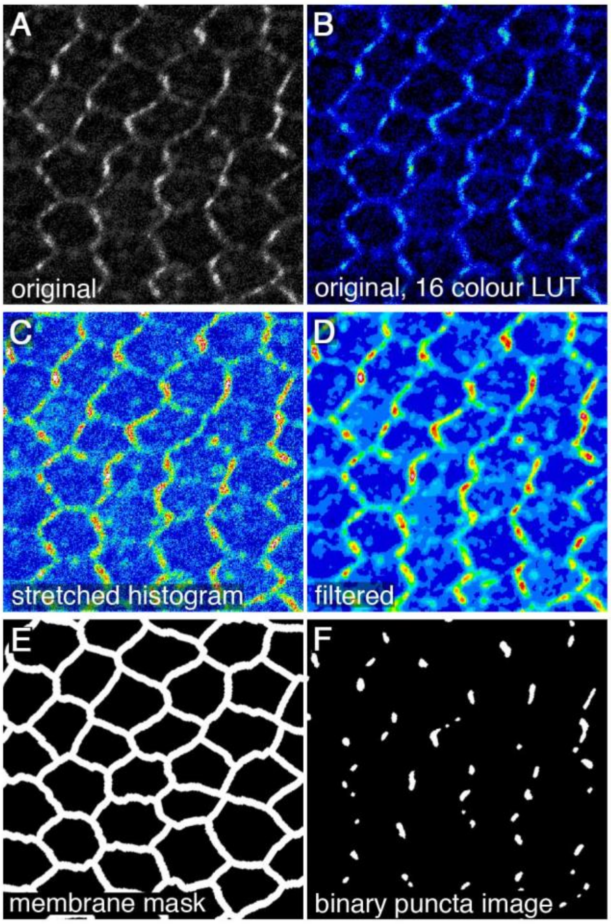

Supplemental Experimental Procedures Fig 1. Detecting puncta.

(A, B) An original live image from a pupal wing expressing *fz-EGFP*, in greyscale (A) or with a 16 colour lookup table (B). (C, D) The same image after stretching the histogram so that the top 0.1% and bottom 0.1% of data are saturated (C), and after applying a Gaussian filter followed by a median filter to reduce noise (D). (E) The membrane mask after being dilated and eroded to smooth its edges, then re-dilated to a radius of 4 pixels. (F) A binary image of detected puncta.

Puncta were detected in the stretched/filtered image using the bwconncomp function of MATLAB, and objects smaller than 9 pixels were then excluded. A simple threshold value for object recognition could not be used, as different genotypes have different overall intensities, and images are different distances from the coverslip (see below). Therefore we made the assumption that puncta in different genotypes would have the same average size, and used MATLAB to find a threshold value for each image whereby puncta occupied 10% of the total area of the junctions. By eye, this appeared to select all the large puncta. The regionprops function was then applied to the original, unstretched, unfiltered image, to measure mean puncta intensity, number and area. The mean intensity of membranes and cytoplasm was also determined. When gene dosage was altered, puncta intensity varied similarly to overall membrane intensity, suggesting that puncta are not significantly larger or smaller, and validating the use of a fixed membrane proportion to define puncta. Increasing or decreasing the area defined as puncta did not significantly alter the relative puncta intensities.

Background due to autofluorescence was determined by imaging wings containing clones of *EGFP-pk*. This value was very low, typically half the value of the laser off background, and was subtracted. After correcting for wing-coverslip distance (see below), the mean puncta intensity was averaged for each wing (1-3 images were taken per wing, depending on the flatness). At least 8 wings were imaged for most genotypes: an initial dataset was used to do a power calculation using G*Power, which suggested that this would be sufficient to distinguish between differences of intensities of 10:7, with a power of 0.8. Mean puncta intensity was then averaged across wings. Unpaired t-tests were used for pair-wise comparisons, and for multiple comparisons, ANOVA with Tukey's test was used to compare all genotypes or with Dunnett's test to compare to a control genotype. For comparing multiple genotypes at different stages or in puncta and non-puncta, 2-way ANOVA with Holm-Šídák correction was used.

For quantitating puncta in clones in fixed images, wild-type and mutant regions were separated, and puncta detection applied to each region separately. Due to antibody staining background and possible non-linearity of staining, measured puncta intensity in wild-type and mutant tissue is unlikely to scale linearly with real protein amounts; however it can be determined whether puncta intensities in mutant tissue are significantly higher or lower than in control tissue. For twin clone experiments comparing tagged to untagged protein (Fig S1 and Table S1), wings containing mutant clones were stained in parallel, and the intensity within the clone was subtracted as background on the twin clone images, leading to more accurate values. Puncta intensity was compared between control and mutant regions in the same wing using paired t-tests.

For measuring puncta asymmetry on the borders of *fmi-EGFP*, *fz-EGFP* and *stbm-EGFP* twin clones, puncta were detected over the whole image on the basis of Fmi staining. Puncta on proximal and distal cell ends were selected manually in ImageJ, and mean intensity and puncta area were measured for Fmi, Fz or Stbm. Proximal and distal puncta intensity were averaged per wing, and compared in the same wing using paired t-tests. For clones of *fz-EGFP* and *stbm-EGFP*, mean puncta area was similar for proximal and distal puncta.

Correcting for wing-coverslip distance

Pupal wings sit inside an impermeable membrane, and during live imaging the distance of the wing from the coverslip varies between wings and between different regions of the same wing. By plotting puncta intensity of different wings of the same genotype against distance from the coverslip, puncta intensity was shown to decrease exponentially with distance from the coverslip according to the light attenuation equation $I = I_0 * \exp(-\alpha * X)$, where I is the light at X distance from the coverslip, I_0 is intensity at the coverslip and α is the attenuation coefficient (Supplemental Experimental Procedures Fig 2). To determine the attenuation coefficient for 28 hr APF pupal wing fluid, an initial dataset of about 20 images from each of 25 genotypes was collected. Prism was used to fit curves for each genotype, assuming that the attenuation coefficient was the same for all genotypes, and the best-fit value was extracted (α=0.0186). This value was then used in subsequent experiments to extrapolate puncta intensity to I_0. The attenuation coefficient was calculated separately for 20 hr APF pupal wings, where the dorsal and ventral wing epithelia are not yet apposed, and the apical wing surface is generally closer to the pupal wing membrane (α=0.0317).

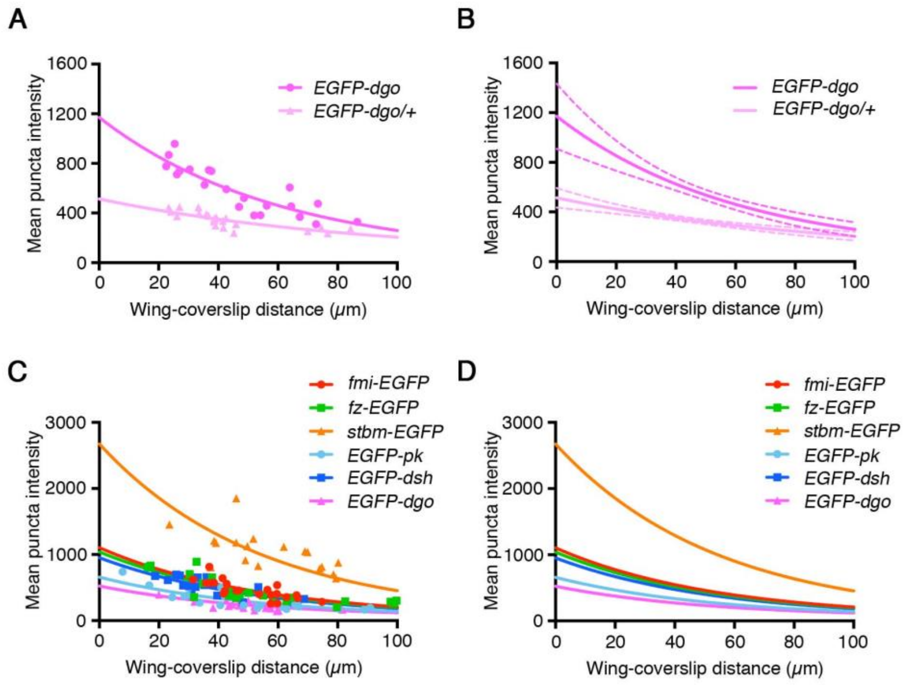

Supplemental Experimental Procedures Fig 2. Correcting for wing-coverslip distance.

(A, B) Mean puncta intensity in live images of pupal wings from *P[acman]-EGFP-dgo dgo*^{380} and *P[acman]-EGFP-dgo dgo*^{380}/+, plotted against distance between the wing and the coverslip. (A) Individual points fit to one-phase exponential decay curves. (B) Exponential decay curves with 95% confidence intervals (dashed lines).

(C, D) Mean puncta intensity in the live images of pupal wings used for stoichiometry in Fig. 1E, plotted against wing-coverslip distance. The exponential decay curve was fitted using an experimentally-determined attenuation coefficient (see Materials and Methods), shown with the individual data points (C) or without (D).

Polarity measurement

To determine polarity using MATLAB, image intensity histograms were stretched and membrane masks were dilated and eroded as for puncta detection. An inverse cytoplasmic mask was generated and each cell identified as an object using bwconncomp. For each cell, the centroid was identified and the perimeter extracted. For each point on the perimeter of a cell a circular mask of 3 pixels was placed and the average pixel intensities and the angle from the centroid was determined. Cell outlines were approximated to a circle, and data placed into 360 bins, depending on their angle. The intensity data in groups of 90 bins were compared, to identify the angles at which the maximum asymmetry was observed (e.g. intensity in bins 0-90 plus 280-270, compared to bins 90-180 plus 270-360). This gave a mean polarity and angle for each cell; this vector polarity was then averaged for all cells in the image, to give a mean vector polarity (asymmetry ratio on plots). The standard deviation in the cell-by-cell polarity angle was used as a measure of the coordination in polarity between cells.

For live images laser off background and autofluorescent background were subtracted prior to polarity measurement. Averaging between wings and statistical tests were similar to those for puncta detection.

For fixed wings, laser off background was subtracted, but an unknown amount of antibody staining background was present in each image, so the resulting degree of asymmetry tended to be lower than for live images. Polarity values were compared between control and mutant regions in the same wing using paired t-tests.

Measuring intensity on lateral junctions

To measure intensity on proximal-distal and lateral junctions, a variant on the MATLAB script for polarity measurement was used. After identifying each cell, the centroid was determined. The cytoplasm was dilated by 8 pixels and the original cytoplasm mask subtracted to give a membrane mask for each cell. The intensity of each pixel within this mask on the original image was determined, together with its angle from the centroid. The 90° bins giving maximum asymmetry for each cell were then determined as for polarity measurement, and these intensities were assigned as proximal-distal junctions, whilst the intensities in orthogonal bins were assigned as lateral junctions.

Supplemental References

Aigouy, B., Farhadifar, R., Staple, D.B., Sagner, A., Röper, J.-C., Julicher, F., and Eaton, S. (2010). Cell flow reorients the axis of planar polarity in the wing epithelium of *Drosophila*. Cell *142*, 773-786.

Bastock, R., and Strutt, D. (2007). The planar polarity pathway promotes coordinated cell migration during *Drosophila* oogenesis. Development *134*, 3055-3064.

Chae, J., Kim, M.J., Goo, J.H., Collier, S., Gubb, D., Charlton, J., Adler, P.N., and Park, W.J. (1999). The *Drosophila* tissue polarity gene *starry night* encodes a member of the protocadherin family. Development *126*, 5421-5429.

Huang, J., Zhou, W., Watson, A.M., Jan, Y.N., and Hong, Y. (2008). Efficient ends-out gene targeting in *Drosophila*. Genetics *180*, 703-707.

Rawls, A.S., and Wolff, T. (2003). Strabismus requires Flamingo and Prickle function to regulate tissue polarity in the *Drosophila* eye. Development *130*, 1877-1887.

Strutt, D.I. (2001). Asymmetric localisation of Frizzled and the establishment of cell polarity in the *Drosophila* wing. Mol Cell *7*, 367-375.

Strutt, H., Price, M.A., and Strutt, D. (2006). Planar polarity is positively regulated by casein kinase Iε in *Drosophila*. Curr Biol *16*, 1329-1336.

Strutt, H., and Strutt, D. (2008). Differential stability of Flamingo protein complexes underlies the establishment of planar polarity. Curr Biol *18*, 1555-1564.

Strutt, H., Thomas-MacArthur, V., and Strutt, D. (2013). Strabismus promotes recruitment and degradation of farnesylated Prickle in *Drosophila melanogaster* planar polarity specification. PloS Genetics *9*, e1003654.

Strutt, H., Warrington, S.J., and Strutt, D. (2011). Dynamics of core planar polarity protein turnover and stable assembly into discrete membrane subdomains. Dev Cell *20*, 511-525.

Usui, T., Shima, Y., Shimada, Y., Hirano, S., Burgess, R.W., Schwarz, T.L., Takeichi, M., and Uemura, T. (1999). Flamingo, a seven-pass transmembrane cadherin, regulates planar cell polarity under the control of Frizzled. Cell *98*, 585-595.

Warrington, S.J., Strutt, H., and Strutt, D. (2013). The Frizzled-dependent planar polarity pathway locally promotes E-cadherin turnover via recruitment of RhoGEF2. Development *140,* 1045-1054.

Wasserscheid, I., Thomas, U., and Knust, E. (2007). Isoform-specific interaction of Flamingo/Starry Night with excess Bazooka affects planar cell polarity in the *Drosophila* wing. Dev Dynamics *236*, 1064-1071.
