# Supplementary material for: Robust Asymmetric Localization of Planar Polarity Proteins Is Associated with Organization into Signalosome-like Domains of Variable Stoichiometry
Source: Cell Rep. 2016 Dec 6;17(10):2660–71. doi: 10.1016/j.celrep.2016.11.021 (PMC5177602; doi:10.1016/j.celrep.2016.11.021)
Supplement: Document S2. Article plus Supplemental Information [file mmc3.pdf]

Cell Reports

Article

Robust Asymmetric Localization of Planar Polarity Proteins Is Associated with Organization into Signalosome-like Domains of Variable Stoichiometry

Graphical Abstract

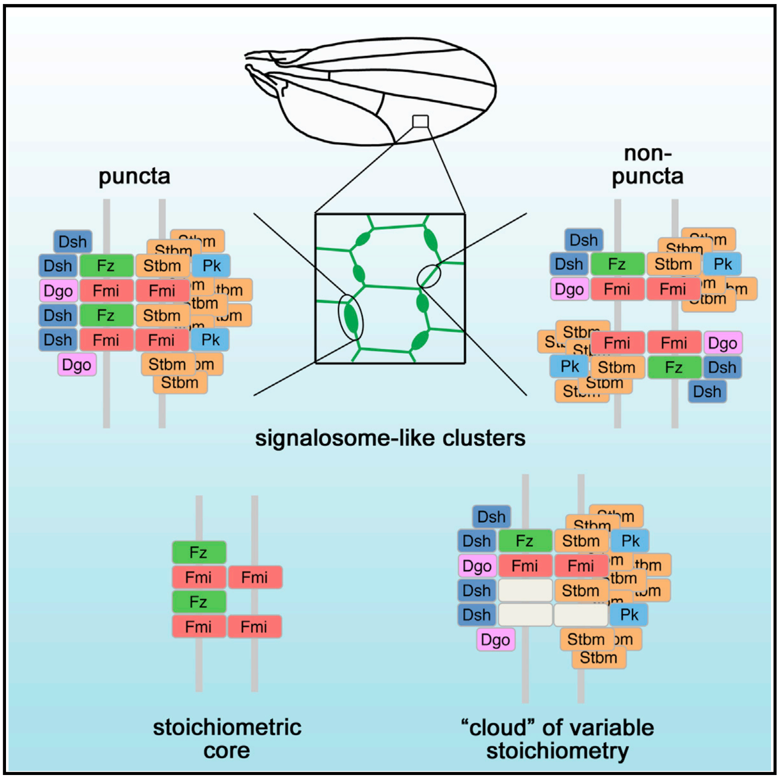

Highlights

- Planar polarity proteins form signalosome-like complexes of variable stoichiometry
- Flamingo and Frizzled form a stoichiometric nucleus within the complex
- Asymmetric localization of core proteins is robust to changes in complex composition
- Core protein stability in complexes is not dependent on complex stoichiometry

Authors

Helen Strutt, Jessica Gamage, David Strutt

Correspondence

d.strutt@sheffield.ac.uk

In Brief

Strutt et al. show that the composition of the core planar polarity complex is highly plastic, with stable association of complex components occurring over a wide range of stoichiometries and with cell polarization being robust to changes in complex composition, suggesting a signalosome-like organization.

CrossMark

Strutt et al., 2016, Cell Reports *17*, 2660–2671
December 6, 2016 © 2016 The Author(s).
http://dx.doi.org/10.1016/j.celrep.2016.11.021

CellPress

OPEN ACCESS
CellPress

Cell Reports
Article

Robust Asymmetric Localization of Planar Polarity Proteins Is Associated with Organization into Signalosome-like Domains of Variable Stoichiometry

Helen Strutt,[1,2] Jessica Gamage,[1,2] and David Strutt[1,3,*]
[1]Bateson Centre, Department of Biomedical Science, University of Sheffield, Western Bank, Sheffield S10 2TN, UK
[2]Co-first author
[3]Lead Contact
*Correspondence: d.strutt@sheffield.ac.uk
http://dx.doi.org/10.1016/j.celrep.2016.11.021

SUMMARY

In developing epithelia, the core planar polarity proteins physically interact with each other and localize asymmetrically at opposite cell ends, forming intercellular complexes that link the polarity of neighboring cells. Using quantitative imaging to examine the composition of the core protein complex in vivo, we find that complex composition is unexpectedly plastic. The transmembrane proteins Frizzled and Flamingo form a stoichiometric nucleus in the complex, while the relative levels of the other four core proteins can vary independently. Exploring the functional consequences of this, we show that robust cell polarization is achieved over a range of complex stoichiometries but is dependent on maintaining appropriate levels of the components Frizzled and Strabismus. We propose that the core proteins assemble into signalosome-like structures, where stable association is not dependent on one-to-one interactions with binding partners, and signaling functions can act over a wide range of complex compositions.

INTRODUCTION

During animal development, many epithelia are polarized in the plane of the tissue. One of the best-characterized systems that controls this planar polarity involves the core planar polarity proteins (known hereafter as the core proteins). These localize asymmetrically within cells at the level of the adherens junctions and control the production of polarized structures and polarized cell behavior (Goodrich and Strutt, 2011; Wallingford, 2012).

The core proteins have been well studied in the *Drosophila* pupal wing epithelium. Here, they localize asymmetrically on proximal and distal cell edges and regulate the orientation of an actin-rich trichome, which emerges from the distal end of each cell. The seven-pass transmembrane protein Frizzled (Fz) localizes distally, with the cytoplasmic proteins Dishevelled (Dsh) and Diego (Dgo); Strabismus (Stbm; also known as Van Gogh), a four-pass transmembrane protein, localizes proximally with the cytoplasmic protein Prickle (Pk); and the atypical cadherin Flamingo (Fmi; also known as Starry Night) localizes both proximally and distally, where it can mediate homophilic adhesion between neighboring cells (Figure 1A). Thus, the core proteins form an intercellular complex, bridging neighboring cells and allowing them to coordinate their polarity (Strutt and Strutt, 2009; Devenport, 2014).

Fmi and Fz form the essential nucleus of this complex. In the absence of Fmi activity, Fz, Dsh, and Dgo are lost from junctions (Axelrod, 2001; Feiguin et al., 2001; Shimada et al., 2001; Strutt, 2001) and Stbm and Pk levels are reduced (Bastock et al., 2003; Tree et al., 2002). In turn, if Fz is absent, Fmi localizes poorly to junctions and is predominantly found in the apical plasma membrane (Usui et al., 1999; Strutt and Strutt, 2008). Furthermore, the Fmi-Fz interaction is a key symmetry breaking step: Fmi localizes better to junctions between cells when Fz is only present in one cell than it does when Fz is present in both cells (Strutt and Strutt, 2008; see also Struhl et al., 2012). The activity of the other four core proteins is required to redistribute these Fmi:Fmi-Fz intercellular complexes so that they are localized at one end of the cell, in the same orientation, with the overall direction of polarity being dependent on upstream tissue-specific cues (Goodrich and Strutt, 2011; Devenport, 2014).

The molecular mechanisms that sort the core proteins to opposite cell ends are not understood. However, the core proteins can self-organize: clones of cells lacking Fz activity recruit core proteins to clone boundaries (Usui et al., 1999; Strutt, 2001), resulting in a reversal of polarity on one side of the clone, which can be propagated over several rows of cells (Vinson and Adler, 1987). Moreover, computational models have shown that positive and negative feedback interactions between the core proteins may be sufficient to amplify a slight bias in localization or activity of one of the proteins (e.g., Amonlirdviman et al., 2005; Le Garrec et al., 2006; Burak and Shraiman, 2009; Schamberg et al., 2010).

Cellular asymmetry of the core proteins correlates with their concentration into membrane subdomains that we term puncta (Figure 1B): asymmetry within puncta is greater than in other junctional regions (Strutt et al., 2011; Cho et al., 2015).

This is an open access article under the CC BY license (http://creativecommons.org/licenses/by/4.0/).

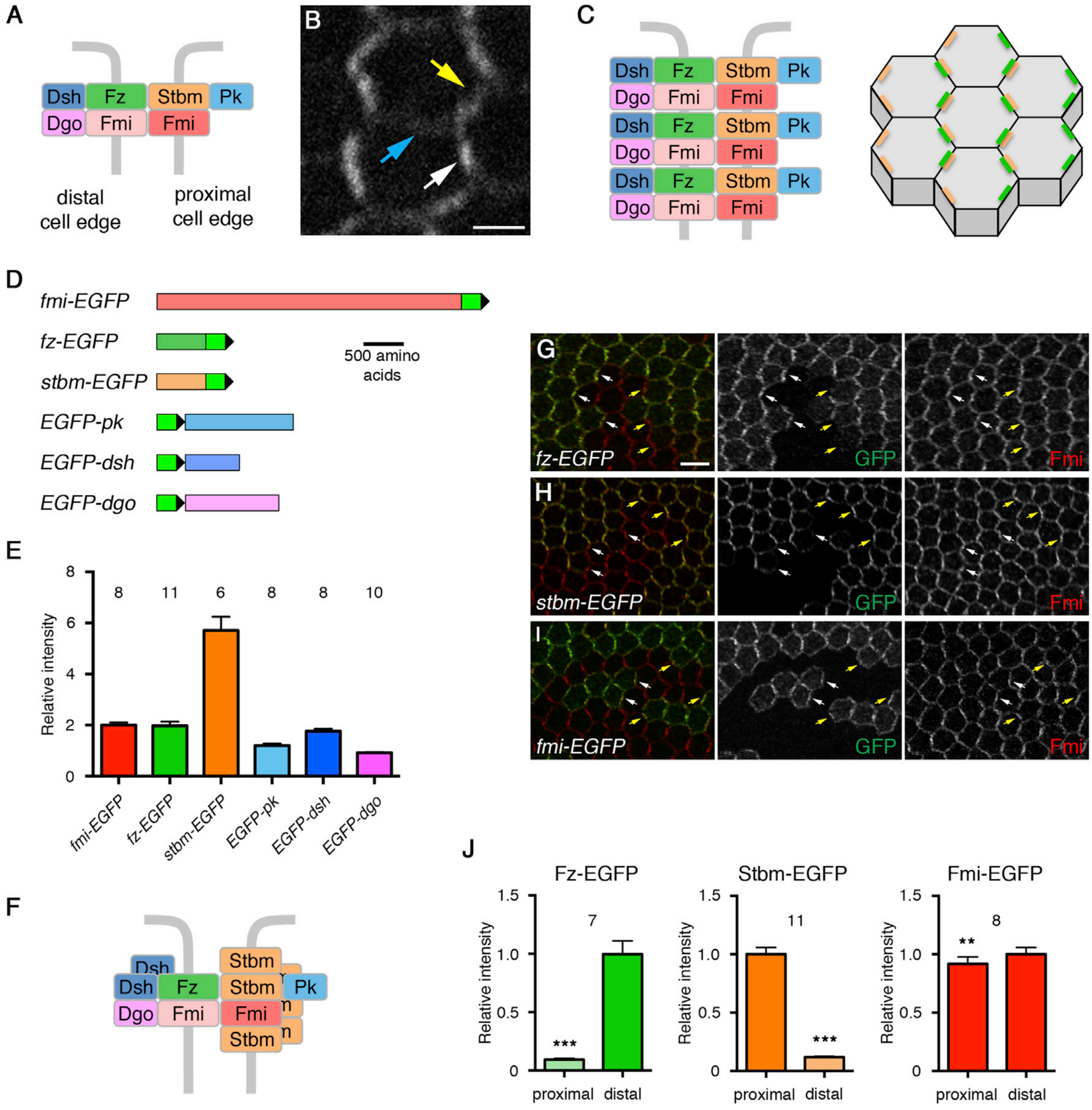

Figure 1. Core Planar Polarity Complex Stoichiometry

(A) Diagram of the core proteins localizing to proximal and distal cell ends, based on known localizations and protein-protein interactions.

(B) Live image of a *fmi-EGFP* pupal wing. Core proteins localize predominantly at the level of the apical adherens junctions. Arrows point to a punctum (white) and a non-punctum region (yellow) in the proximal-distal junctions and to a lateral junction (blue) where no puncta are seen. Proximal is to the left and distal is to the right in this and all later images. Scale bar, 2 μm.

(C) Core protein complexes in the same orientation undergo local clustering in membrane subdomains (puncta, left), which leads to overall cellular asymmetry (right, where green represents distal Fz-containing complexes in puncta and orange represents proximal Stbm-containing complexes in puncta).

(D) Diagram of the tagged core proteins. Bright green is EGFP, and the black triangle is the position of the residual *LoxP* site (not to scale). Fmi, Fz, Pk: EGFP tag inserted into the endogenous genomic locus by in vivo homologous recombination. Stbm, Dsh, Dgo: EGFP tag inserted into a P[acman] rescue construct.

(E) Mean intensity of the EGFP-tagged core proteins in puncta at 28 hr after puparium formation (APF), normalized to 2 units of Fmi-EGFP. Flies were homozygous for the tagged gene. ANOVA analysis shows that Fmi/Fz/Dsh and Pk/Dgo are not significantly different to each other. On this and all subsequent graphs, the number of wings is indicated above the respective column.

(F) Diagram of the core complex in puncta, based on stoichiometry data. Note half the amount of Fz is shown here, compared to the stoichiometry graph, to compensate for there being more Fz-EGFP than endogenous Fz in puncta.

(G–I) High-resolution images of *fz-EGFP* (G), *stbm-EGFP* (H), and *fmi-EGFP* (I) twin clones with untagged protein, revealing asymmetric cellular localizations on clone boundaries. Arrows point to puncta on distal (white) or proximal (yellow) cell boundaries. Scale bar, 5 μm.

(J) Mean intensity of puncta on proximal and distal cell edges. Fmi-EGFP is slightly enriched distally, where Fz is higher; also, Fmi-EGFP puncta are significantly smaller (84%, $p = 0.02$, paired t test) on proximal cell edges than on distal cell edges. ***$p < 0.001$; **$p < 0.01$ (paired t test comparing proximal and distal puncta in the same wing).

Error bars are SEM.

Furthermore, core proteins within puncta are highly stable, with lower rates of turnover than elsewhere in the junctions (Strutt et al., 2011; see also Butler and Wallingford, 2015; Chien et al., 2015). Thus, we hypothesize that the feedback interactions that sort the core proteins onto proximal and distal membranes primarily act locally, resulting in the production of spatially distinct, polarized membrane subdomains (Figure 1C).

There are many unanswered questions about the nature of the core complex and how its components become sorted into puncta. First, the molecular interactions between the complex components are incompletely characterized, and the overall composition of the complex is not known. Stbm, Pk, Dsh, and Dgo can each interact directly with each of the others (Tree et al., 2002; Jenny et al., 2003, 2005; Das et al., 2004), and the mouse Fz homolog Fzd5 can directly bind the Dsh homolog Dvl1 (Tauriello et al., 2012). Fmi and Fz, and mouse homologs of Fmi and Stbm (Celsr1 and Vangl2), have also been reported to co-immunoprecipitate (Chen et al., 2008; Devenport and Fuchs, 2008), and Stbm, Pk, and Dsh/Dvl can homodimerize (Jenny et al., 2003; Kishida et al., 1999). How these interactions translate into a functioning complex in vivo, and how they might promote sorting, remains unclear. Current hypotheses are that Pk and Stbm inhibit recruitment of Dsh to junctions by Fz (Amonlirdviman et al., 2005), that Dgo and Pk compete for binding to Dsh (Jenny et al., 2005), and that Pk mediates negative feedback interactions by excluding Stbm-Fmi complexes from junctions (Cho et al., 2015).

Second, it is not understood why the core proteins within puncta are more stable than those not in puncta. One possibility is that the composition of the complex might change as the core proteins become sorted into puncta. For example, there is evidence that the cytoplasmic proteins (Pk, Dsh, and Dgo) promote clustering (Feiguin et al., 2001; Tree et al., 2002; Bastock et al., 2003), so their levels might increase as core proteins enter puncta. Alternatively, if these cytoplasmic proteins mediate negative feedback interactions, their levels might decrease as asymmetry increases, as they are no longer needed.

Finally, it is not known why asymmetry is robust to changes in core protein levels, as such changes might be expected to disrupt feedback interactions. Loss of Dsh ubiquitination leads to the accumulation of Dsh and the other core proteins at junctions (Strutt et al., 2013a); nevertheless, only minor defects in core protein asymmetry are seen. However, the degree to which feedback is robust to changes in individual protein concentrations has not been systematically tested.

Here, we carried out a detailed study of core protein levels at cell junctions. We assumed that the proteins detected at junctions are all part of complexes, as genetic studies have shown that individual core proteins have little or no localization to junctions in the absence of their transmembrane partners (Axelrod, 2001; Feiguin et al., 2001; Shimada et al., 2001; Strutt, 2001; Tree et al., 2002; Bastock et al., 2003; Das et al., 2004; Strutt and Strutt, 2008). This allowed us to use relative concentrations at junctions to infer core complex composition. We examined protein levels both by immunolabeling of endogenous proteins and by live imaging of EGFP-tagged proteins. Immunolabeling shows qualitative differences in protein levels but is not fully quantitative, as detection may be non-linear, antibodies may saturate, and background staining may vary. For quantitative analyses, we measured the light intensity released from an EGFP tag by live imaging (Coffman and Wu, 2012). By comparing the fluorescence intensities of different tagged molecules, relative concentrations can be determined. This technique has been utilized in many contexts, from microorganisms to vertebrates (Chiu et al., 2002; Damle et al., 2006; McGill et al., 2009). Importantly, the amount of GFP fluorescence has been shown to increase linearly with the number of fluorescent molecules in vitro (Chiu et al., 2001) and in vivo (Wu and Pollard, 2005), suggesting that addition of the EGFP tag to different molecules does not affect EGFP fluorescence.

Using this methodology, we have determined the relative concentrations of each of the core proteins at junctions in pupal wings in vivo. By manipulating gene dosage, we then investigated how modulating complex composition affects asymmetry. This gives us unexpected insights into the relationships among core complex composition, sorting into asymmetric junctional puncta, and the acquisition of cellular asymmetry, and it leads us to suggest that the complex is organized into signalosome-like structures.

RESULTS

In Vivo Stoichiometry of the Core Planar Polarity Proteins in Puncta

To determine the relative stoichiometry of the core planar polarity proteins in vivo, we tagged each of them with EGFP (Figure 1D) and then imaged pupal wings expressing each tagged protein under the same conditions.

For this approach, the tagged proteins must all be expressed at endogenous levels. For Fmi, Fz, and Pk, the EGFP tag was inserted into the endogenous locus by in vivo homologous recombination. For Stbm, Dsh, and Dgo, the EGFP tag was inserted into a P[acman] rescue construct by recombineering and then integrated into the genome; the transgenic animals were then crossed into appropriate mutant backgrounds to maintain normal gene dosage.

The EGFP-tagged proteins localized asymmetrically at junctions in the pupal wing, and trichome polarity in the adult fly wing was normal, suggesting that the proteins can replace endogenous protein function (Figure S1). Western blotting was used to compare the overall cellular levels of core proteins in pupal wings expressing only the tagged protein to those expressing only endogenous protein (Figure S2). Finally, twin clone experiments were used to compare the amount of tagged and untagged protein in junctional puncta, as well as the degree of asymmetry (Figure S1; Tables S1 and S2). Five of the core proteins behaved similarly to the endogenous proteins in these assays. However, Fz-EGFP was expressed at higher levels than endogenous Fz as detected by western blotting, and more was seen in puncta, which we speculate may be because the EGFP tag alters Fz stability. Therefore, measurements of core protein stoichiometry will show artificially high levels of Fz-EGFP. Importantly, however, for all the core proteins, the intensity of EGFP fluorescence in flies expressing one copy of tagged protein and one copy of endogenous protein was approximately half that of flies expressing two copies of tagged protein (Table

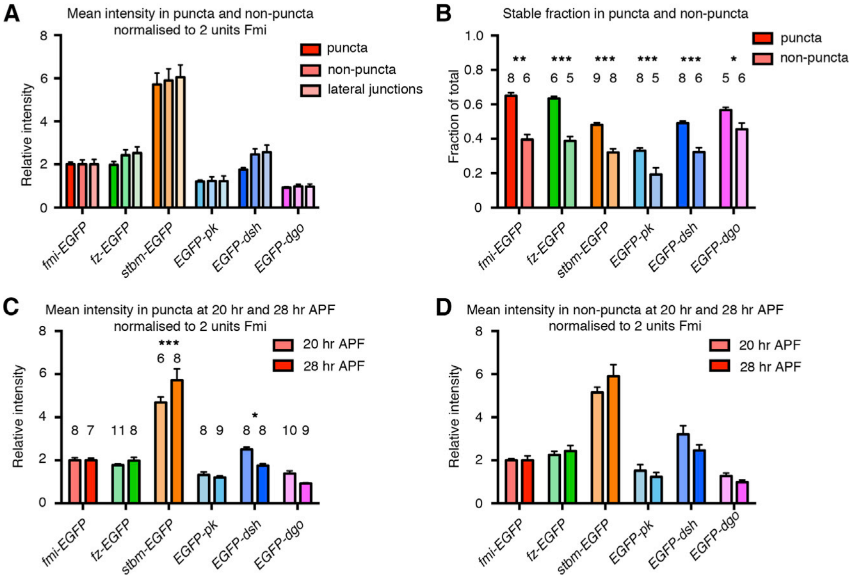

Figure 2. Stoichiometry and Stability in Puncta and Non-puncta Junctional Domains

(A) Mean intensity of EGFP fluorescence in puncta, non-puncta and lateral junctions at 28 hr APF, normalized to 2 units of Fmi-EGFP in each region. Slight increases in the relative levels of Fz-EGFP and EGFP-Dsh are seen in non-puncta and lateral junctions, but these are not statistically significant (comparing puncta and lateral junctions, $p = 0.17$ for Fz-EGFP and $p = 0.06$ for EGFP-Dsh, two-way ANOVA). Sample sizes as in Figure 1E.

(B) Stable proportions of each tagged protein, as determined by FRAP analysis at 28 hr APF, in puncta and non-puncta. ***$p < 0.001$; **$p < 0.01$; *$p < 0.05$ (curve plateaux compared using an extra sum-of-squares F test).

(C and D) Mean intensity of EGFP-tagged core proteins in puncta (C) and non-puncta (D) at 20 hr APF and 28 hr APF, normalized to 2 units of Fmi-EGFP. ***$p < 0.001$; *$p < 0.05$ (20 hr and 28 hr values compared by two-way ANOVA).

Error bars are SEM.

S1). This suggests that the tagged proteins compete similarly to the endogenous proteins for inclusion into puncta.

We first measured the stoichiometry of the core proteins within puncta, as core proteins in puncta are predominantly stable and highly asymmetrically organized, consistent with ordered arrays of aligned complexes (Figure 1C). As a control, we ascertained that all puncta have similar compositions; co-immunolabeling pupal wings for Fmi and each of the EGFP-tagged core proteins showed that Fmi intensity in different puncta increased linearly with EGFP intensity (Figure S3). Live imaging of pupal wings was then carried out on flies expressing each of the tagged core proteins, and the mean intensity of EGFP fluorescence in puncta was determined (see Experimental Procedures).

Comparing mean EGFP puncta intensity in each of the fly lines showed that for every two molecules of Fmi-EGFP within puncta, there are approximately two molecules of Fz-EGFP and EGFP-Dsh, six molecules of Stbm-EGFP, and one molecule of EGFP-Pk and EGFP-Dgo (Figure 1E). As more Fz-EGFP than endogenous Fz is seen in puncta in twin clone experiments (Figure S1; Table S1), we estimate that the amount of endogenous Fz in puncta would be closer to one molecule for every two Fmi molecules. Therefore, instead of puncta exhibiting one-to-one ratios of the different components (e.g., Figure 1A), we see a more complex organization, as summarized in Figure 1F.

We then investigated the degree of asymmetry of the core proteins within puncta by making twin clones and examining puncta intensity on clone boundaries, where tissue expressing tagged protein was adjacent to tissue expressing untagged protein. As previously observed (Strutt et al., 2011), Fz-EGFP is highly enriched in distal puncta compared to proximal puncta, while the reverse is seen for Stbm-EGFP. Fmi-EGFP levels are similar in proximal and distal puncta (Figures 1G–1J). This is consistent with the view that within puncta core proteins are assembled into complexes of the approximate composition shown in Figure 1F, aligned in a common orientation.

Core Protein Stoichiometry Is Similar in Puncta and Non-puncta Junctional Domains, Despite Differing Stable Protein Proportions

In junctional domains that lack large puncta, core protein complex distribution is less ordered, and complexes are less stable (Strutt et al., 2011; see below). We hypothesized that core complexes in these regions might not be fully assembled and the stoichiometry of the complex in these regions might therefore be different. To test this, we determined the intensity of fluorescence in junctions, excluding the puncta. As this may not fully exclude smaller puncta from the "non-puncta" regions, we also measured the fluorescence intensity on lateral junctional domains, where puncta are not observed (Figure 1B; Strutt et al., 2011). Puncta were roughly three times as bright as non-puncta and lateral cell junctions (Figure S4A), but no significant difference was observed in the relative stoichiometry of the core proteins in puncta, non-puncta, or lateral junctions (Figure 2A).

The similar relative core protein levels and corresponding complex composition in puncta and non-puncta were surprising, considering that Fz and Fmi show lower stability in non-puncta (Strutt et al., 2011). We therefore investigated whether all core proteins show reduced stability in non-puncta regions.

Stable proportions of the endogenously expressed tagged core proteins were determined using fluorescence recovery after photobleaching (FRAP). All six core proteins had a larger stable proportion in puncta than non-puncta, although the stable proportion varied for each protein (Figure 2B). Interestingly, Fmi and Fz had similar stable proportions in puncta and also smaller similar stable proportions in non-puncta, consistent with them forming a nucleus to the complex (Strutt and Strutt, 2008).

In summary, overall core protein stoichiometry is similar regardless of whether complexes are concentrated in stable ordered domains (puncta) or more sparsely distributed in less ordered non-puncta regions. However, the stable and unstable proportions of the core proteins vary between puncta regions

and non-puncta regions. This indicates an uncoupling between complex composition and protein stability, such that stability is not promoted by an altered complex composition but by the local concentration of complexes of the same orientation.

Stoichiometry Is Different at an Earlier Stage of Development

As core complex stoichiometry is similar in puncta and non-puncta, one possibility was that a single optimal composition is necessary for junctional localization of the core protein complex. If this were true, stoichiometry would also be the same in tissue in which core protein localization is overall less asymmetric. At earlier stages of wing development, cells are undergoing junctional remodelling; the core proteins show only weak cellular asymmetry and form only small puncta (Aigouy et al., 2010; Strutt et al., 2011).

Interestingly, complex stoichiometry was different in earlier-stage wings. The relative amount of Stbm in puncta was lower in younger wings, while the relative amount of Dsh was increased (Figure 2C). Similar trends were seen in non-puncta, but the differences were not statistically significant (Figures 2D, S4B, and S4C). We conclude that in fact complex stoichiometry is not fixed.

The Stoichiometry of the Cytoplasmic Proteins in Complexes Is Dependent on Expression Levels

We next investigated what happens to stoichiometry if gene dosage of one of the core proteins is altered. First, we examined the effects of halving the dosage of the three cytoplasmic core proteins (Pk, Dsh, or Dgo). Pupal wings expressing two copies of EGFP-tagged protein and no endogenous protein were compared to wings carrying one copy of tagged protein in a heterozygous null mutant background. In each case, the levels of EGFP-tagged protein at junctions was roughly halved in the heterozygous mutant (Figures 3A–3C; Table S3). This was true for both puncta or non-puncta, suggesting that the amount of the cytoplasmic proteins is limiting and that furthermore, under conditions of reduced protein, there is not preferential recruitment to puncta. This was confirmed by immunostaining endogenous proteins (Figures S5A–S5C). Interestingly, halving the junctional amount of any of the cytoplasmic proteins had little effect on the levels of any of the other core proteins, either in puncta or non-puncta (Figures 3D–3I; Table S3).

P[acman] constructs in wild-type instead of mutant backgrounds were then used to double gene dosage. Doubling Dsh or Dgo dosage caused the amount of protein in puncta to increase, consistent with idea that levels of these components are normally limiting (Figures 3J, 3K, and S5D–S5F). Again, this did not alter the levels of other core proteins (Figure 3L–3N; Table S3). This indicates that excess cytoplasmic protein can enter core protein complexes, resulting in variable stoichiometries relative to the transmembrane proteins that recruit them, and that their levels in puncta are limited by their concentration within the cell rather than by specific binding partners.

Computational modeling has suggested that the cytoplasmic proteins play key roles in promoting feedback interactions between the core proteins (e.g., Amonlirdviman et al., 2005; Le Garrec et al., 2006; Burak and Shraiman, 2009; Schamberg et al., 2010). However, modulating their levels in puncta had no effect on core protein asymmetry (Figures 3O and 3P), indicating that feedback and generation of asymmetry does not depend on an exact ratio or concentration of particular cytoplasmic core proteins at the cell junctions.

The Core Complex Is Assembled Around a Stoichiometric Frizzled-Flamingo Nucleus

We then examined what happens to the other core proteins if the dosage of Fmi is altered. As with the cytoplasmic core proteins, halving *fmi* dosage caused a reduction in protein levels, both in puncta and non-puncta (Figures 4A and S5G; Table S3). Notably, halving the amount of Fmi at junctions caused a corresponding decrease in levels of Fz-EGFP (Figures 4B and S5H; Table S3). This suggests that Fmi levels control how much Fz enters the complex, consistent with the idea that Fmi and Fz form a stoichiometric nucleus for the complex. In contrast, levels of the other complex components were negligibly affected (Figures 4C–4F, S5I, and S5J; Table S3), and cellular asymmetry was also unaffected (Figure 4G).

fmi gene dosage was also increased by expressing a *P[acman]-fmi-EGFP* rescue construct in a wild-type background. This caused an increase in cellular protein levels but only a very mild increase in levels of Fmi in puncta (Figures S5K and S5L; Table S3), suggesting that the amount of Fmi that can enter puncta is limited.

We hypothesized that the plasticity in puncta composition might be explained by differences in core protein stability. In particular, the stable amounts of the core proteins might maintain a constant stoichiometry relative to each other, but this might be masked by the presence of an additional unstable population that varies depending on cellular concentration.

We first considered whether decreasing Fmi levels in the cell and hence in puncta might primarily result in a loss of the unstable Fmi in puncta, and thus, there would be an increase in the proportion of stable Fmi. FRAP analysis showed that this was not the case: when Fmi levels were decreased by halving *fmi* dosage, its stable proportion was unaltered, and thus, both the stable and unstable amounts were decreased (Figure 4H). Similarly, the stable proportion of Fz was also unchanged in *fmi* heterozygotes, leading to smaller stable and unstable amounts (Figure 4I). Thus, the stable ratios of Fmi and Fz remain constant.

We next examined whether the stable proportions of Stbm, Pk, Dsh, and Dgo decrease when *fmi* dosage is lowered to maintain a constant ratio of stable protein. Surprisingly, the stable amounts of these proteins were unaltered (Figures 4J–4M), indicative of varying stable ratios relative to Fmi and Fz. Taken together, these results are consistent with the conclusion that Fmi and Fz maintain a stoichiometric, stable nucleus, while the levels and stability of the other core proteins can vary independently of this.

Maintaining Levels of Frizzled and Strabismus within the Complex Is Essential for Strong Asymmetry

Halving *fz* dosage had little effect on its levels in puncta (Figure S6A), and in western blots, no significant decrease in the amount of Fz in the cell was seen (Figure S6E). As some Fz is normally targeted to the lysosome (Strutt and Strutt, 2008),

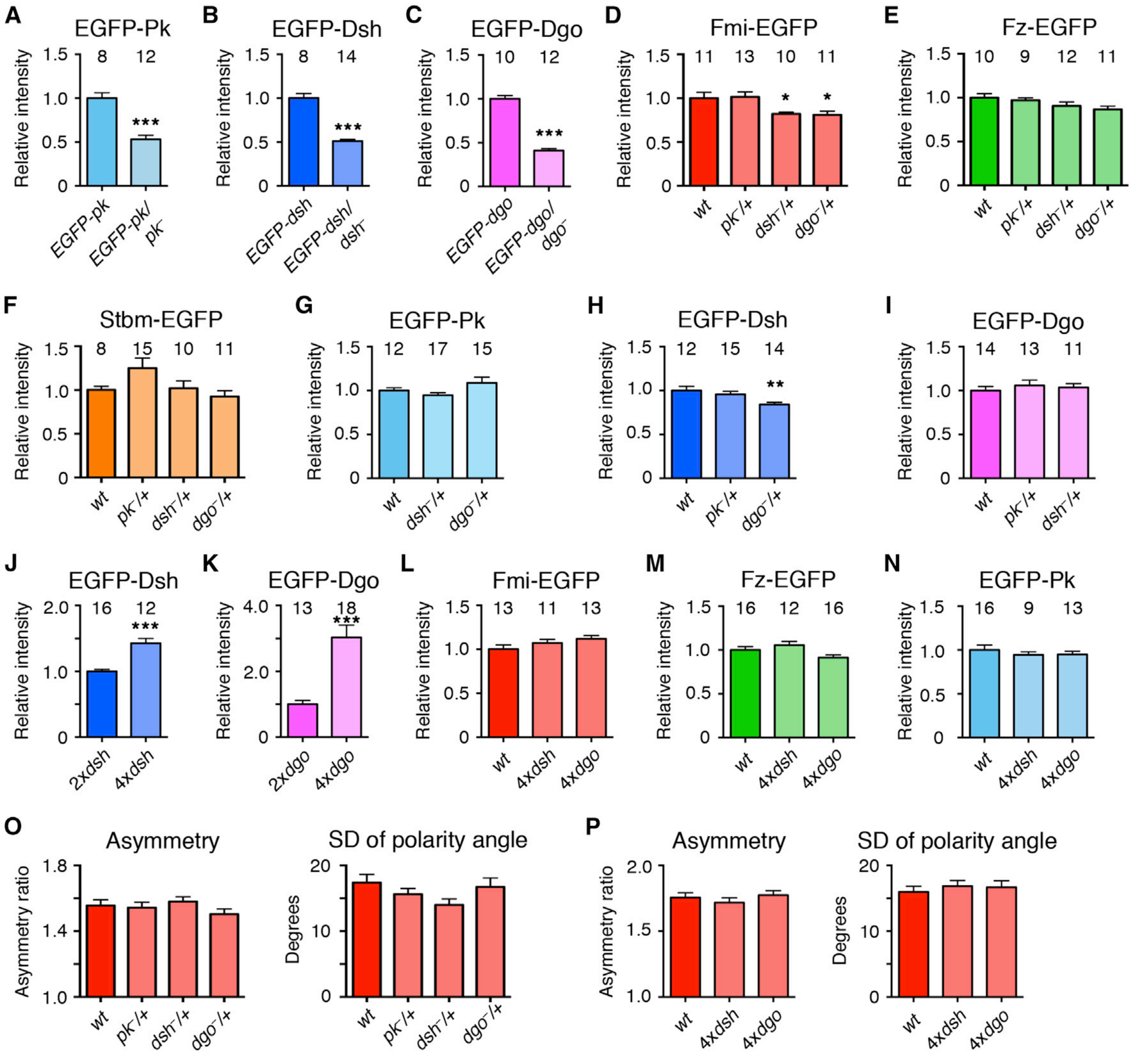

Figure 3. The Effects on Complex Stoichiometry of Altering Gene Dosage of Cytoplasmic Core Proteins

(A–C) Relative mean intensity of puncta in live images of wings homozygous for the indicated EGFP-tagged core gene or carrying one copy of the EGFP-tagged gene in a null mutant background. ***p < 0.001 (ANOVA comparison to wild-type).

(D–I) Relative mean intensity of puncta in live images of wings carrying EGFP-tagged core proteins, in a wild-type background, or in wings heterozygous for *pk-sple*^{13}, *dsh*^{V26}, or *dgo*^{380}. **p < 0.01; *p < 0.05 (ANOVA comparison to wild-type).

(J and K) Relative mean intensity of puncta in live pupal wings, comparing flies carrying one dose of endogenous gene and one dose of tagged gene with those carrying two doses of endogenous gene and two doses of tagged gene. Note that when *dgo* dosage is doubled, three times as much Dgo enters puncta, possibly due to cooperative effects. ***p < 0.001 (unpaired t test).

(L–N) Relative mean intensity of Fmi-EGFP (L), Fz-EGFP (M), and EGFP-Pk (N) puncta in live images of wild-type wings or wings homozygous for *P[acman]-dsh* or *P[acman]-dgo*. Data compared to wild-type by ANOVA.

(O and P) Fmi-EGFP asymmetry and SD of polarity angle in images from live pupae of wings heterozygous for *pk-sple*^{13}, *dsh*^{V26}, and *dgo*^{380} (O) or in flies homozygous for *P[acman]-dsh* or *P[acman]-dgo* (P) (see Experimental Procedures for details of polarity quantitation). There were no significant differences to wild-type (ANOVA). Sample sizes as in (D) and (L).

Error bars are SEM.

degradation of excess protein may be reduced when gene dosage is lowered in order to maintain levels at junctions. Interestingly, increased Fz levels in puncta (as a result of the higher cellular levels of Fz-EGFP compared to endogenous Fz; see Figure S1H) had no effect on the levels of other core proteins tested (Figures S6C and S6D). In particular, Fmi levels are not increased, suggesting that although there is a stoichiometric nucleus of one molecule of Fz to two molecules to Fmi, such that a minimum threshold level of Fmi is required to initiate Fz recruitment (Figure 4B), once this nucleus is present above a certain concentration, more Fz can become incorporated into complexes.

Halving *stbm* dosage also did not affect Stbm levels in puncta (Figure S6B), although in this case, cellular levels were

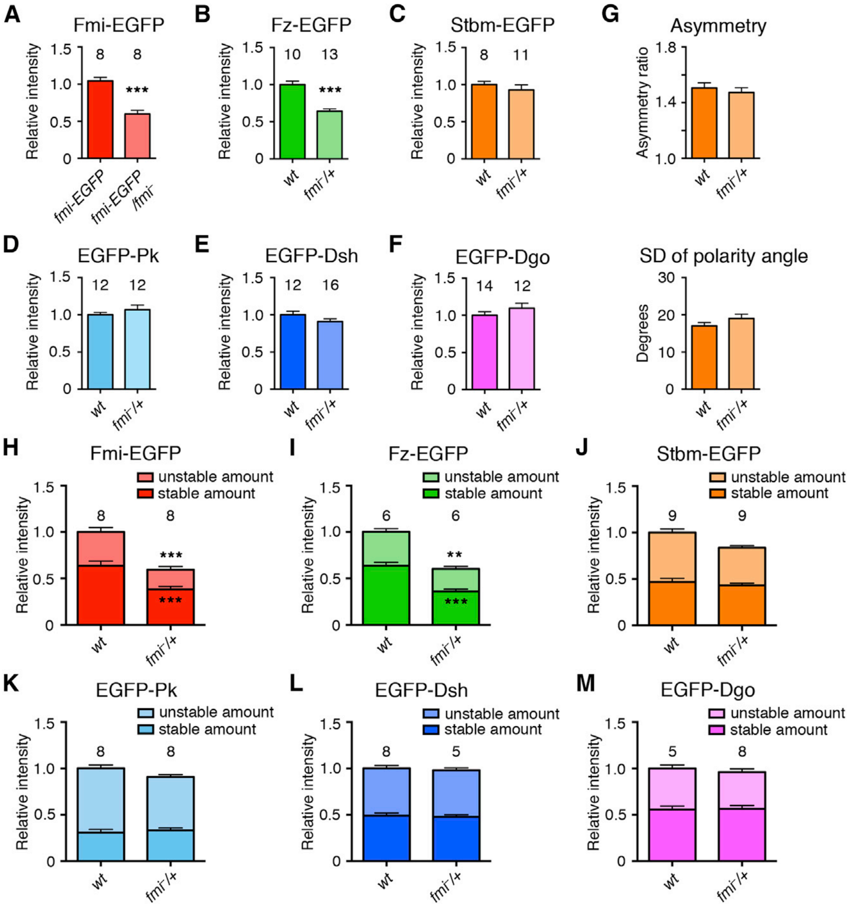

Figure 4. The Core Complex Contains a Stoichiometric Nucleus of Flamingo and Frizzled

(A–F) Relative mean intensity of puncta in wings carrying EGFP-tagged core proteins in a wild-type background or in wings heterozygous for *fmi*^{E59}. ***p < 0.001 (ANOVA).

(G) EGFP asymmetry and SD of polarity angle in live images of wings expressing *stbm-EGFP*, in a wild-type background, or in flies heterozygous for *fmi*^{E59} (as above). Similar results were seen imaging *fmi-EGFP*. No significant difference between wild-type and mutant was seen (ANOVA).

(H–M) Stable and unstable amounts of EGFP-tagged core proteins in puncta in a wild-type background or in wings heterozygous for *fmi*^{E59}. Stable proportions, as determined by FRAP, were multiplied by total puncta intensity. Unpaired t tests were used to test for significance of changes in the stable amount (lower asterisks) or the total amount of protein (upper asterisks). ***p < 0.001; **p < 0.01.

decreased (Figure S6F). Furthermore, doubling *stbm* gene dosage increased the amount in the cell (Figure S6F), but no more was seen in puncta (Figure S6G). This suggests that Stbm protein is in excess in the cell, but no more can enter junctional complexes.

In order to test if maintaining levels of Fz and Stbm in puncta is important for feedback and generation of asymmetry, we expressed both proteins at artificially low levels using single copies of transgenes under control of the *armadillo* (*arm*) promoter, and we then examined their levels in clones lacking endogenous protein. In both cases, the levels of protein in puncta were modestly decreased (Figures 5A and 5C; Table S4). This caused only minor changes in junctional levels of the other core proteins (Figures S7A–S7H; Table S4). However, reduced levels of Fz led to some Fmi localizing at the apical plasma membrane, as in *fz* mutant tissue (Figure 5B, Strutt and Strutt, 2008). A slight increase in Pk levels was seen when Stbm levels in puncta were lowered, consistent with Stbm negatively regulating Pk levels (Figure 5D, Strutt et al., 2013b).

Strikingly, when the amount of either Fz or Stbm in puncta was lowered, even by a modest degree, asymmetry was significantly reduced (Figures 5E and 5F) and trichome orientation defects were seen (Figures S7I and S7J). Therefore, while levels of the other core proteins in the complex can be altered without affecting asymmetry, reduced levels of Fz and Stbm relative to their binding partners cause strong defects in cell polarization.

Asymmetry Is Sensitive to Large Modulations of Core Complex Stoichiometry

Finally, we asked whether asymmetry can also be disrupted by more severe alterations in core complex composition. As halving *fmi* dosage did not affect asymmetry, we lowered *fmi* dosage further, by expressing a single copy of *fmi* under the *arm* promoter, in *fmi* mutant clones. A clear decrease in the levels of Fmi, Fz, and Stbm was observed by immunostaining (Figures 6A–6C; Table S4), but there was only a mild reduction in levels of Pk, Dsh, and Dgo (Figures S7L–S7N; Table S4). However, core protein asymmetry was reduced (Figure 6D), suggesting that a threshold amount of Fmi is necessary for robust polarization. However, this degree of reduced asymmetry was not sufficient to cause defects in trichome orientation (Figure S7O).

Second, we examined the effects of halving the dosage of multiple core genes simultaneously. Notably, while the levels of Fmi in puncta were only mildly altered when the dosage of a single core gene was halved (Figure 3D), Fmi levels were significantly decreased in triple or quadruple heterozygotes (Figure 6E). This was accompanied by a decrease in asymmetry (Figure 6F, mild defects in trichome orientation are seen; see Figures S7P and S7Q). Therefore, we conclude that the composition of the core complex can vary considerably without any deleterious effects on overall asymmetry, but the extent of this plasticity is limited to within-threshold levels of each protein.

DISCUSSION

There are two key challenges for the core pathway in coordinating cell polarity within developing tissues: first to be able to respond dynamically to polarizing cues, and second to establish

OPEN ACCESS
CellPress

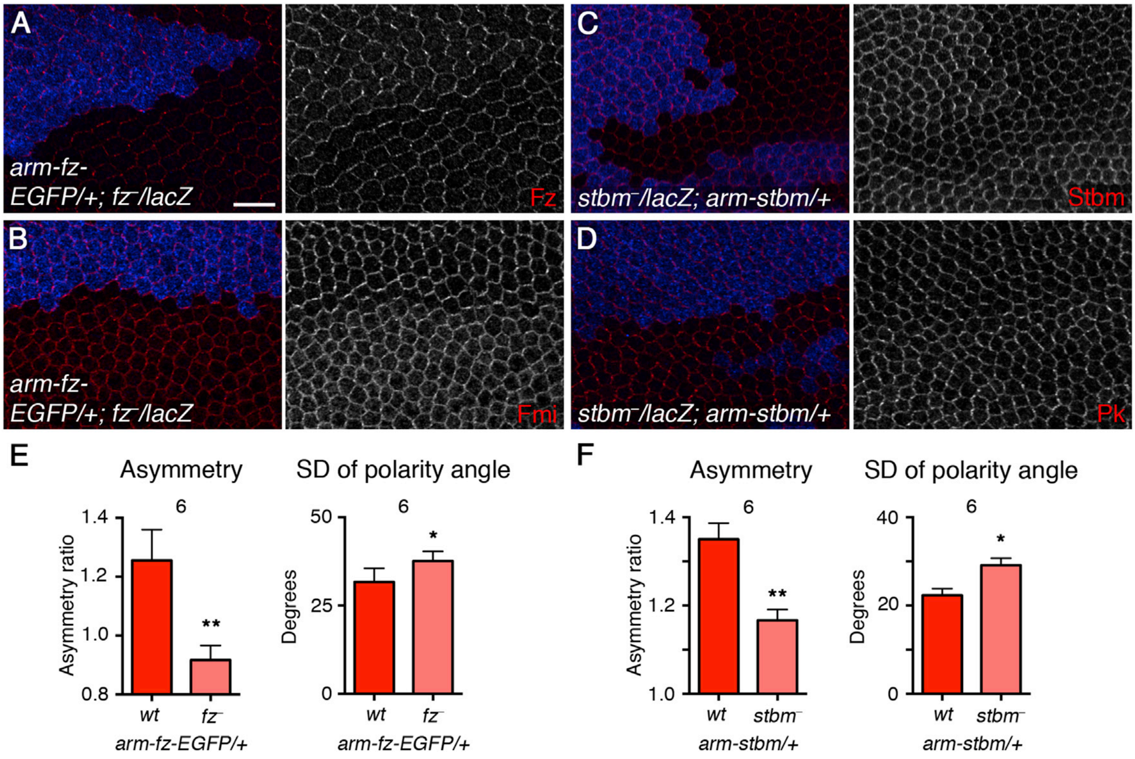

Figure 5. Decreasing Cellular Levels of Frizzled or Strabismus Causes Defects in Asymmetry

(A and B) Pupal wings carrying one copy of *arm-fz-EGFP*, with fz^{P21} clones marked by loss of β-gal staining (blue). Wings stained for Fz (A, red) or Fmi (B, red). Note that higher levels of the Fz-EGFP fusion used here have been shown to function normally (Strutt, 2001; Figure S7K). Scale bar, 10 μm.

(C and D) Pupal wings carrying one copy of *arm-stbm*, with $stbm^6$ clones marked by loss of β-gal staining (blue). Wings stained for Stbm (C, red) or Pk (D, red).

(E and F) Asymmetry and SD of polarity angle. (E) Pupal wings carrying one copy of *arm-fz-EGFP*, in fz^{P21} clones or in twinspot tissue (wild-type background). (F) Pupal wings carrying one copy of *arm-stbm*, in $stbm^6$ clones or in twinspot tissue (wild-type background). Wings were immunostained for Fmi, but similar results were obtained by Fz or Stbm immunostaining. Note that the overall variation in the polarity angle is high in wild-type tissue next to fz^{P21} clones (averaging 30°), because of the strong boundary effects, leading to non-autonomous defects on the orientation of core protein localization outside of the clone. **$p < 0.0$; *$p < 0.05$ (paired t test used to compare polarity in wild-type tissue and mutant clones in the same wing).

Error bars are SEM.

a sufficiently stable polarized state. These two requirements act in tension and suggest that pathway organization requires features in common both with rapidly responding signaling pathways and with long-lasting structural components of cells. To investigate how the core pathway achieves this balance, we have investigated the in vivo organization of the core planar polarity complex and how this translates into sorting of core proteins into stable membrane subdomains and cellular asymmetry.

Using quantitative imaging of the core complex in pupal wings, we first determined the in vivo stoichiometry of the complex and further showed that this is not fixed, with levels of the cytoplasmic components and Stbm able to vary relative to levels of Fz and Fmi. In contrast, levels of Fz and Fmi are interdependent, consistent with the notion that these proteins form a stoichiometric nucleus within the complex. Importantly, the stable proportions of core proteins at junctions are maintained over a range of different complex stoichiometries. Furthermore, complex composition is the same in more stable locally polarized domains and less stable unpolarized domains; thus, the size of the stable proportions is correlated with local order of polarity, not complex composition. Finally, asymmetry is robust to changes in complex composition, but the amount of Fz and Stbm within the complex must be maintained relative to their binding partners for normal asymmetry.

A caveat to our approach is that our ability to measure local complex stoichiometry is limited to the resolution of confocal microscopy (~200 nm), whereas protein complexes would be expected to be at least an order of magnitude smaller. Nevertheless, the lack of variation in our measurements between different puncta and in junctions overall suggests that there is little spatial variation in complex composition in cell junctions, and the figures we obtain can be regarded as plausible local averages of complex composition.

The cytoplasmic core proteins depend on the transmembrane proteins for their recruitment to junctions (Axelrod, 2001; Feiguin et al., 2001; Shimada et al., 2001; Tree et al., 2002; Bastock et al., 2003; Das et al., 2004). However, we find that relative levels of individual cytoplasmic proteins within the complex can increase or decrease independently of the other proteins. For example, Dsh and Dgo can be present at levels that are several-fold higher than the levels of their binding partners Fz and Fmi. We therefore suggest a "cloud model," whereby complex composition is

OPEN
ACCESS
CellPress

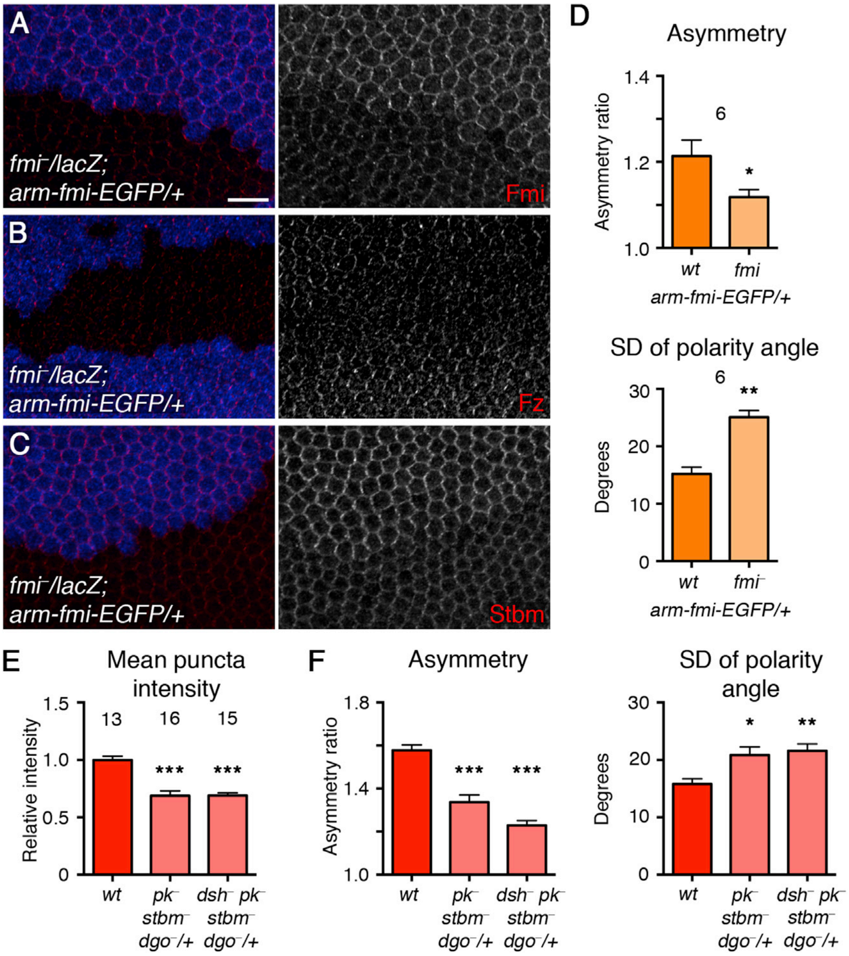

Figure 6. The Effects of Large Decreases in Flamingo Levels and Large Modulations in Core Complex Stoichiometry

(A–C) Pupal wings carrying one copy of *arm-fmi-EGFP*, with fmi^{E59} clones marked by loss of β-gal staining (blue). Wings labeled for Fmi (A), Fz (B), and Stbm (C) in red. Scale bar, 10 μm.

(D) Asymmetry and SD of polarity angle for wings carrying one copy of *arm-fmi-EGFP*, in fmi^{E59} clones or in twinspot tissue (wild-type background). Wings were immunostained for Stbm, but similar results were obtained by immunostaining for other core proteins. **$p < 0.01$; *$p < 0.5$ (paired t test used to compare polarity in wild-type tissue and mutant clones in the same wing).

(E) Relative mean intensity of puncta in *fmi-EGFP/+* live pupal wings in a wild-type background and in flies triply heterozygous for $pk\text{-}sple^{13}$, $stbm^{6}$, and dgo^{380} or quadruply heterozygous for dsh^{V26}, $pk\text{-}sple^{13}$, $stbm^{6}$, and dgo^{380}. ***$p < 0.001$ relative to wild-type (ANOVA).

(F) Asymmetry and SD of polarity angle, measuring EGFP fluorescence in the same genotypes as in (E). ***$p < 0.001$; **$p < 0.01$; *$p < 0.05$ relative to wild-type (ANOVA).

Error bars are SEM.

determined by cellular concentration rather than by stoichiometric binding interactions. We propose that the core proteins need a minimum concentration of Fmi and Fz to nucleate at junctions. Above this threshold concentration, multiple binding interactions allow a cloud of Stbm, Pk, Dsh, and Dgo to associate at junctions (Figures 7B–7D). Binding sites have been mapped between Stbm and Pk, as well as between Dsh and Dgo (Jenny et al., 2003, 2005), and Stbm, Pk, and Dsh contain dimerization motifs (Jenny et al., 2003; Kishida et al., 1999). In addition to this, Fmi, Fz, Stbm, and Dsh contain putative PDZ (PSD-95, Discs Large, ZO-1) binding motifs of unknown function, which may interact with PDZ-containing scaffolding factors (Wolff and Rubin, 1998; Djiane et al., 2005; Wasserscheid et al., 2007; Johnston et al., 2013). Membrane interaction motifs, for example the DEP (Dishevelled, Egl10, and Pleckstrin) domain (Simons et al., 2009) and the Pk prenylation motif (Jenny et al., 2003; Lin and Gubb, 2009; Strutt et al., 2013b), may also promote association of the core proteins to junctions independently of protein-protein interaction sites. A further possibility is that once recruited to the complex, locally occurring post-translational modifications may alter protein mobility, allowing complex components to remain associated even in the absence of an ongoing direct interaction with a binding partner. For example, Dsh is phosphorylated only upon recruitment to junctions by Fz (Axelrod, 2001; Shimada et al., 2001).

Interestingly, the features we describe in our cloud model bear a striking resemblance to those seen for higher-order assemblies of signaling molecules known as signalosomes (Bienz, 2014; Wu and Fuxreiter, 2016). Such complexes are thought to be dynamic clusters of signaling molecules that typically assemble at ligand-bound receptors. Polymerization of signaling molecules into signalosomes increases their local concentration, often non-stoichiometrically, and cooperativity of assembly allows threshold responses to ligand stimulation. We therefore propose that core proteins assemble into signalosome-like structures, but importantly, unlike in the conventional view of signalosomes, core protein assembly is not a transient response to ligand binding but part of the process of robust establishment of stable cell polarity.

Another feature of signalosomes is that the polymerization of downstream signaling proteins is nucleated by a core that is often sub-stoichiometric (Wu and Fuxreiter, 2016)—a role that could be fulfilled by Fmi and Fz. We previously proposed that Fmi and Fz form a nucleus for the complex, which is key for symmetry breaking (Figure 7A; Strutt and Strutt, 2008). Consistent with this, we now show that Fz and Fmi levels at junctions have a stoichiometric relationship. Furthermore, FRAP analysis shows that similar proportions of Fmi and Fz are stable, both in puncta and non-puncta.

One of our original hypotheses was that the overall composition of the core complex might be different in highly polarized puncta, where protein stability is high, than in disordered

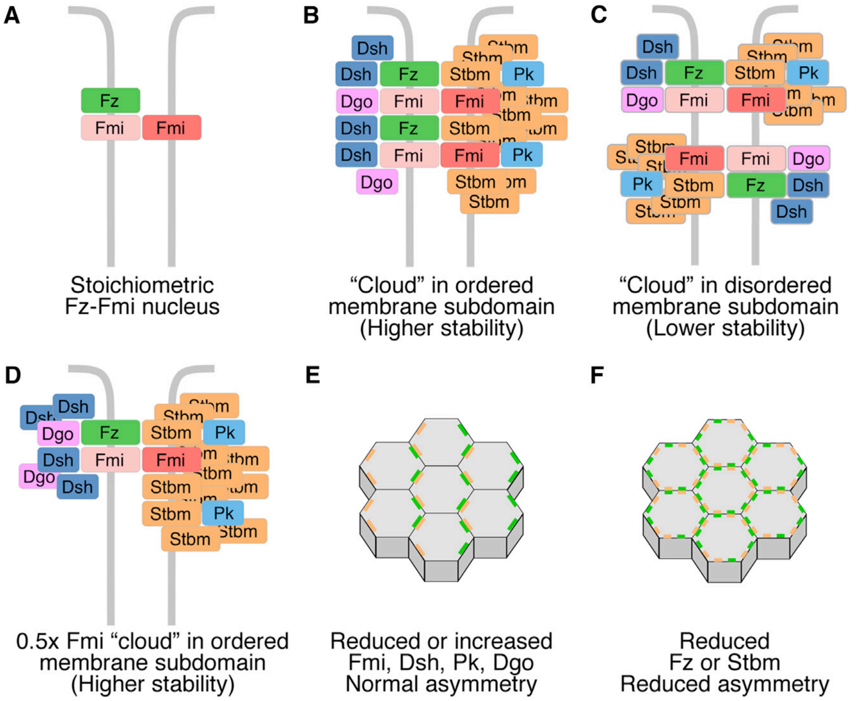

Figure 7. Cloud Model of Core Planar Polarity Complex Composition

(A) Fz levels are dependent on Fmi levels, consistent with Fmi and Fz forming a stoichiometric nucleus to the complex.

(B and C) Above a threshold concentration of Fmi and Fz at junctions, the core proteins form a signalosome-like cloud around the Fz-Fmi nucleus. Puncta contain ordered arrays of complexes of the same composition, which show higher stability, possibly due to cooperative interactions (B). Non-puncta have complexes of the same overall composition as puncta, but they are less ordered and less densely packed and core proteins are less stable (C).

(D) The amount of core proteins associating in this cloud at junctions is dependent on their cellular concentration, but not on stoichiometric interactions with the Fz-Fmi nucleus, resembling the organization seen in signalosomes. For instance, halving Fmi levels reduces the amount of Fmi and Fz at junctions but does not alter the amounts of the other core proteins or their stability.

(E) Altering levels of Fmi, Pk, Dsh, or Dgo at junctions alters the stoichiometry of the complex, but asymmetry is normal.

(F) Reducing levels of Fz and Stbm at junctions disrupts asymmetry.

non-puncta, where stability is lower. However, we find that the composition of the complex is very similar in puncta and non-puncta. Why then is core protein stability higher in puncta?

We suggest that there are two levels of organization of the core proteins. In both puncta and non-puncta, they nucleate around Fz-Fmi backbones into sub-microscopic signalosome-like structures of similar composition (Figures 7B and 7C). Then, under the influence of positive feedback interactions, some of these domains grow into locally ordered puncta of the same composition but higher stability. Clustering into puncta is most likely a result of cooperative interactions, whereby complexes of similar orientation associate with each other, dependent on their local concentration. Such *cis* interactions may then result in increased stability and lower turnover. An example of this is the immunological synapse, in which protein-protein interactions cause diffusional trapping and clustering of signaling molecules (Douglass and Vale, 2005). Similarly, in vitro experiments have suggested that multivalent complexes undergo phase transitions, and spontaneously cluster together once their local concentration exceeds a threshold value (Li et al., 2012). In addition, clustering of the core proteins into puncta may be associated with the formation of stable interactions with the cytoskeleton. For example, the stability of E-cadherin clusters at adherens junctions is dependent on the actin cytoskeleton (reviewed in Yap et al., 2015), and cortical actin activity also regulates the mobility of GPI-linked proteins in nanoclusters (Goswami et al., 2008).

Most feedback models describing planar polarity rely on mass action kinetics, whereby proteins interact and exert positive and negative feedback, depending on their relative concentration (e.g., Amonlirdviman et al., 2005; Le Garrec et al., 2006; Burak and Shraiman, 2009; Schamberg et al., 2010). Pk, Dsh, and Dgo have been suggested to be key factors mediating feedback (Amonlirdviman et al., 2005; Jenny et al., 2005; Cho et al., 2015), but we have found that their relative concentrations can vary considerably, without any apparent defects in protein asymmetry (Figure 7E). One possibility is that feedback interactions are slowed down when relative concentrations are changed, but no defect is apparent as the system reaches a steady state. In addition, multiple redundant feedback interactions may exist, and a perturbation in the rate of just one of these will have little overall effect on asymmetry. This would be consistent with our data showing that altering the dosage of one component does not affect asymmetry, but altering stoichiometry more severely (by altering the dosage of several components) is deleterious. Strikingly, however, if the levels of either Fz or Stbm relative to Fmi are lowered, then asymmetry is severely compromised. Therefore, it appears that the core complex cannot undergo normal feedback interactions if the Fz-Fmi:Fmi-Stbm backbone is out of balance (Figure 7F). Interestingly, the exquisite sensitivity of feedback to levels of Fz and Stbm appears to have caused the system to evolve so that their levels are buffered against changes: by altering rates of degradation in the case of Fz or by maintaining excess levels of protein in cellular pools for Stbm.

Overall, our data are consistent with a model in which feedback operates in the context of organization of the core proteins into higher-order signalosome-like structures. We propose that such an organization concentrates components at junctions where they can participate in feedback. This both increases the efficiency of feedback and buffers against fluctuations in protein levels, for example following cell division. Ultimately, this

promotes the biphasic partitioning of the core proteins to opposite cell ends.

EXPERIMENTAL PROCEDURES

Additional information regarding molecular biology, fly stocks, antibodies, imaging, and image analysis is available in Supplemental Experimental Procedures.

Western Blotting

For pupal wing westerns, 28 hr after puparium formation (APF) pupal wings were dissected directly into sample buffer. One pupal wing equivalent was used per lane. A Bio-Rad ChemiDoc XRS+ was used for imaging, and band intensities from three biological replicates were quantified using ImageJ. Data were compared used unpaired t tests or ANOVA for multiple comparisons.

Imaging and FRAP Analysis in Pupal Wings

For live imaging, pupae were prepared and imaged at 28 hr APF (unless otherwise stated) as previously described (Strutt et al., 2011). For FRAP, regions of interest (ROIs) of ~2 μm^2 were selected for puncta and non-puncta. After imaging, ROIs were manually reselected in ImageJ and quantitated. Control unbleached regions were also quantitated to control for acquisition bleaching. Data were corrected for acquisition bleaching and normalized against an average of the prebleached values and the first postbleach value. Data from ROIs in the same wing were averaged, and Prism (v6 GraphPad) was used to fit a one-phase exponential association curve for each wing. Data from several wings were then used to fit a final exponential association curve, and an extra sum-of-squares F test was performed to compare curve plateaux (Y_{max}) between puncta and non-puncta.

To combine the FRAP data with the puncta stoichiometry data, the normalized stoichiometry data were multiplied by the stable and unstable proportions. Standard errors for each experiment were combined, and plotted as the square root of the sum of each error squared. To compare relative stable and unstable amounts in puncta and non-puncta, each dataset was normalized to 2 units of Fmi-EGFP, and two-way ANOVA with Holm-Šídák correction was performed.

To compare stable and unstable amounts between EGFP-tagged protein in a wild-type and *fmi*^{E59} heterozygous background, prebleached values were averaged for each wing and multiplied by the stable proportion ($1 - Y_{max}$). Stable amounts were then averaged across wings and compared using unpaired t tests.

Puncta Detection and Quantitation

Membrane masks were generated using Packing Analyzer (Aigouy et al., 2010), and automated puncta detection was carried out using a MATLAB script (see Supplemental Experimental Procedures and Data S1 for MATLAB scripts). The mean intensity of puncta and non-puncta membranes was determined. For live imaging, background due to autofluorescence was subtracted, and mean puncta intensity was averaged across wings, and compared using unpaired t tests or ANOVA.

For comparing intensity of individual puncta labeled with two different antibodies in the same wing, a puncta mask was generated as above for one channel, and this mask was then used to measure puncta intensity in both channels.

For quantitating puncta in clones in fixed images, wild-type and mutant regions were separated, and puncta detection applied to each region separately. Puncta intensity was compared between control and mutant regions in the same wing using paired t tests.

For measuring puncta asymmetry on the borders of *fmi-EGFP*, *fz-EGFP*, and *stbm-EGFP* twin clones, puncta were detected over the whole image on the basis of Fmi staining. Puncta on proximal and distal cell ends were selected manually in ImageJ, and mean intensity and puncta area were measured for Fmi, Fz, or Stbm. Proximal and distal puncta intensity were averaged per wing and compared in the same wing using paired t tests.

Polarity Measurement

A MATLAB script was used to determine the angle of maximum asymmetry for each cell (see Supplemental Experimental Procedures and Data S1 for supplemental MATLAB scripts). The vector polarity was then averaged for all cells in the image to give a mean vector polarity (asymmetry ratio on plots). The SD in the cell-by-cell polarity angle was used as a measure of the coordination in polarity between cells. Averaging between wings and statistical tests were similar to those for puncta detection.

SUPPLEMENTAL INFORMATION

Supplemental Information includes Supplemental Experimental Procedures, seven figures, four tables, and one data file and can be found with this article online at http://dx.doi.org/10.1016/j.celrep.2016.11.021.

AUTHOR CONTRIBUTIONS

H.S., J.G., and D.S. designed experiments and wrote MATLAB scripts. H.S. and J.G. conducted experiments and analyzed the data. H.S. and D.S. wrote the paper.

ACKNOWLEDGMENTS

We thank Tanya Wolff for Stbm antibody, BACPAC Resources and the Drosophila Genomics Resource Center for plasmids, and Genetivision and Bestgene for generating transformants. We thank Natalia Bulgakova, Kai Erdmann, and Andy Furley for their comments on the manuscript and Nick Brown for helpful discussions and inspiration. This work was funded by a Wellcome senior fellowship (grant number 100986/Z/13/Z) awarded to D.S. and an MRC studentship (grant numbers G0900203-1/1 and G1000405-1/1). Imaging was performed in the Wolfson Light Microscopy Facility, using a confocal microscope provided by the Wellcome Trust (grant number 100986/Z/13/Z).

Received: July 12, 2016
Revised: September 21, 2016
Accepted: November 2, 2016
Published: December 6, 2016

REFERENCES

Aigouy, B., Farhadifar, R., Staple, D.B., Sagner, A., Röper, J.-C., Jülicher, F., and Eaton, S. (2010). Cell flow reorients the axis of planar polarity in the wing epithelium of *Drosophila*. Cell *142*, 773–786.

Amonlirdviman, K., Khare, N.A., Tree, D.R.P., Chen, W.-S., Axelrod, J.D., and Tomlin, C.J. (2005). Mathematical modeling of planar cell polarity to understand domineering nonautonomy. Science *307*, 423–426.

Axelrod, J.D. (2001). Unipolar membrane association of Dishevelled mediates Frizzled planar cell polarity signaling. Genes Dev. *15*, 1182–1187.

Bastock, R., Strutt, H., and Strutt, D. (2003). Strabismus is asymmetrically localised and binds to Prickle and Dishevelled during *Drosophila* planar polarity patterning. Development *130*, 3007–3014.

Bienz, M. (2014). Signalosome assembly by domains undergoing dynamic head-to-tail polymerization. Trends Biochem. Sci. *39*, 487–495.

Burak, Y., and Shraiman, B.I. (2009). Order and stochastic dynamics in *Drosophila* planar cell polarity. PLoS Comput. Biol. *5*, e1000628.

Butler, M.T., and Wallingford, J.B. (2015). Control of vertebrate core planar cell polarity protein localization and dynamics by Prickle 2. Development *142*, 3429–3439.

Chen, W.-S., Antic, D., Matis, M., Logan, C.Y., Povelones, M., Anderson, G.A., Nusse, R., and Axelrod, J.D. (2008). Asymmetric homotypic interactions of the atypical cadherin Flamingo mediate intercellular polarity signaling. Cell *133*, 1093–1105.

Chien, Y.H., Keller, R., Kintner, C., and Shook, D.R. (2015). Mechanical strain determines the axis of planar polarity in ciliated epithelia. Curr. Biol. *25*, 2774–2784.

Chiu, C.S., Kartalov, E., Unger, M., Quake, S., and Lester, H.A. (2001). Single-molecule measurements calibrate green fluorescent protein surface densities

on transparent beads for use with ‘knock-in’ animals and other expression systems. J. Neurosci. Methods *105*, 55–63.

Chiu, C.S., Jensen, K., Sokolova, I., Wang, D., Li, M., Deshpande, P., Davidson, N., Mody, I., Quick, M.W., Quake, S.R., and Lester, H.A. (2002). Number, density, and surface/cytoplasmic distribution of GABA transporters at presynaptic structures of knock-in mice carrying GABA transporter subtype 1-green fluorescent protein fusions. J. Neurosci. *22*, 10251–10266.

Cho, B., Pierre-Louis, G., Sagner, A., Eaton, S., and Axelrod, J.D. (2015). Clustering and negative feedback by endocytosis in planar cell polarity signaling is modulated by ubiquitinylation of Prickle. PLoS Genet. *11*, e1005259.

Coffman, V.C., and Wu, J.Q. (2012). Counting protein molecules using quantitative fluorescence microscopy. Trends Biochem. Sci. *37*, 499–506.

Damle, S., Hanser, B., Davidson, E.H., and Fraser, S.E. (2006). Confocal quantification of cis-regulatory reporter gene expression in living sea urchin. Dev. Biol. *299*, 543–550.

Das, G., Jenny, A., Klein, T.J., Eaton, S., and Mlodzik, M. (2004). Diego interacts with Prickle and Strabismus/Van Gogh to localize planar cell polarity complexes. Development *131*, 4467–4476.

Devenport, D. (2014). The cell biology of planar cell polarity. J. Cell Biol. *207*, 171–179.

Devenport, D., and Fuchs, E. (2008). Planar polarization in embryonic epidermis orchestrates global asymmetric morphogenesis of hair follicles. Nat. Cell Biol. *10*, 1257–1268.

Djiane, A., Yogev, S., and Mlodzik, M. (2005). The apical determinants aPKC and dPatj regulate Frizzled-dependent planar cell polarity in the *Drosophila* eye. Cell *121*, 621–631.

Douglass, A.D., and Vale, R.D. (2005). Single-molecule microscopy reveals plasma membrane microdomains created by protein-protein networks that exclude or trap signaling molecules in T cells. Cell *121*, 937–950.

Feiguin, F., Hannus, M., Mlodzik, M., and Eaton, S. (2001). The ankyrin repeat protein Diego mediates Frizzled-dependent planar polarization. Dev. Cell *1*, 93–101.

Goodrich, L.V., and Strutt, D. (2011). Principles of planar polarity in animal development. Development *138*, 1877–1892.

Goswami, D., Gowrishankar, K., Bilgrami, S., Ghosh, S., Raghupathy, R., Chadda, R., Vishwakarma, R., Rao, M., and Mayor, S. (2008). Nanoclusters of GPI-anchored proteins are formed by cortical actin-driven activity. Cell *135*, 1085–1097.

Jenny, A., Darken, R.S., Wilson, P.A., and Mlodzik, M. (2003). Prickle and Strabismus form a functional complex to generate a correct axis during planar cell polarity signaling. EMBO J. *22*, 4409–4420.

Jenny, A., Reynolds-Kenneally, J., Das, G., Burnett, M., and Mlodzik, M. (2005). Diego and Prickle regulate Frizzled planar cell polarity signalling by competing for Dishevelled binding. Nat. Cell Biol. *7*, 691–697.

Johnston, C.A., Manning, L., Lu, M.S., Golub, O., Doe, C.Q., and Prehoda, K.E. (2013). Formin-mediated actin polymerization cooperates with Mushroom body defect (Mud)-Dynein during Frizzled-Dishevelled spindle orientation. J. Cell Sci. *126*, 4436–4444.

Kishida, S., Yamamoto, H., Hino, S., Ikeda, S., Kishida, M., and Kikuchi, A. (1999). DIX domains of Dvl and Axin are necessary for protein interactions and their ability to regulate β-catenin stability. Mol. Cell. Biol. *19*, 4414–4422.

Le Garrec, J.F., Lopez, P., and Kerszberg, M. (2006). Establishment and maintenance of planar epithelial cell polarity by asymmetric cadherin bridges: a computer model. Dev. Dyn. *235*, 235–246.

Li, P., Banjade, S., Cheng, H.C., Kim, S., Chen, B., Guo, L., Llaguno, M., Hollingsworth, J.V., King, D.S., Banani, S.F., et al. (2012). Phase transitions in the assembly of multivalent signalling proteins. Nature *483*, 336–340.

Lin, Y.Y., and Gubb, D. (2009). Molecular dissection of *Drosophila* Prickle isoforms distinguishes their essential and overlapping roles in planar cell polarity. Dev. Biol. *325*, 386–399.

McGill, M.A., McKinley, R.F.A., and Harris, T.J.C. (2009). Independent cadherin-catenin and Bazooka clusters interact to assemble adherens junctions. J. Cell Biol. *185*, 787–796.

Schamberg, S., Houston, P., Monk, N.A., and Owen, M.R. (2010). Modelling and analysis of planar cell polarity. Bull. Math. Biol. *72*, 645–680.

Shimada, Y., Usui, T., Yanagawa, S., Takeichi, M., and Uemura, T. (2001). Asymmetric colocalization of Flamingo, a seven-pass transmembrane cadherin, and Dishevelled in planar cell polarization. Curr. Biol. *11*, 859–863.

Simons, M., Gault, W.J., Gotthardt, D., Rohatgi, R., Klein, T.J., Shao, Y., Lee, H.J., Wu, A.L., Fang, Y., Satlin, L.M., et al. (2009). Electrochemical cues regulate assembly of the Frizzled/Dishevelled complex at the plasma membrane during planar epithelial polarization. Nat. Cell Biol. *11*, 286–294.

Struhl, G., Casal, J., and Lawrence, P.A. (2012). Dissecting the molecular bridges that mediate the function of Frizzled in planar cell polarity. Development *139*, 3665–3674.

Strutt, D.I. (2001). Asymmetric localization of Frizzled and the establishment of cell polarity in the Drosophila wing. Mol. Cell *7*, 367–375.

Strutt, H., and Strutt, D. (2008). Differential stability of Flamingo protein complexes underlies the establishment of planar polarity. Curr. Biol. *18*, 1555–1564.

Strutt, H., and Strutt, D. (2009). Asymmetric localisation of planar polarity proteins: mechanisms and consequences. Semin. Cell Dev. Biol. *20*, 957–963.

Strutt, H., Warrington, S.J., and Strutt, D. (2011). Dynamics of core planar polarity protein turnover and stable assembly into discrete membrane subdomains. Dev. Cell *20*, 511–525.

Strutt, H., Searle, E., Thomas-Macarthur, V., Brookfield, R., and Strutt, D. (2013a). A Cul-3-BTB ubiquitylation pathway regulates junctional levels and asymmetry of core planar polarity proteins. Development *140*, 1693–1702.

Strutt, H., Thomas-MacArthur, V., and Strutt, D. (2013b). Strabismus promotes recruitment and degradation of farnesylated Prickle in *Drosophila melanogaster* planar polarity specification. PLoS Genet. *9*, e1003654.

Tauriello, D.V., Jordens, I., Kirchner, K., Slootstra, J.W., Kruitwagen, T., Bouwman, B.A., Noutsou, M., Rüdiger, S.G., Schwamborn, K., Schambony, A., and Maurice, M.M. (2012). Wnt/β-catenin signaling requires interaction of the Dishevelled DEP domain and C terminus with a discontinuous motif in Frizzled. Proc. Natl. Acad. Sci. USA *109*, E812–E820.

Tree, D.R.P., Shulman, J.M., Rousset, R., Scott, M.P., Gubb, D., and Axelrod, J.D. (2002). Prickle mediates feedback amplification to generate asymmetric planar cell polarity signaling. Cell *109*, 371–381.

Usui, T., Shima, Y., Shimada, Y., Hirano, S., Burgess, R.W., Schwarz, T.L., Takeichi, M., and Uemura, T. (1999). Flamingo, a seven-pass transmembrane cadherin, regulates planar cell polarity under the control of Frizzled. Cell *98*, 585–595.

Vinson, C.R., and Adler, P.N. (1987). Directional non-cell autonomy and the transmission of polarity information by the *frizzled* gene of *Drosophila*. Nature *329*, 549–551.

Wallingford, J.B. (2012). Planar cell polarity and the developmental control of cell behavior in vertebrate embryos. Annu. Rev. Cell Dev. Biol. *28*, 627–653.

Wasserscheid, I., Thomas, U., and Knust, E. (2007). Isoform-specific interaction of Flamingo/Starry Night with excess Bazooka affects planar cell polarity in the *Drosophila* wing. Dev. Dyn. *236*, 1064–1071.

Wolff, T., and Rubin, G.M. (1998). *Strabismus*, a novel gene that regulates tissue polarity and cell fate decisions in *Drosophila*. Development *125*, 1149–1159.

Wu, H., and Fuxreiter, M. (2016). The structure and dynamics of higher-order assemblies: amyloids, signalosomes, and granules. Cell *165*, 1055–1066.

Wu, J.Q., and Pollard, T.D. (2005). Counting cytokinesis proteins globally and locally in fission yeast. Science *310*, 310–314.

Yap, A.S., Gomez, G.A., and Parton, R.G. (2015). Adherens junctions revisualized: Organizing cadherins as nanoassemblies. Dev. Cell *35*, 12–20.

Cell Reports, Volume *17*

Supplemental Information

Robust Asymmetric Localization of Planar Polarity Proteins Is Associated with Organization into Signalosome-like Domains of Variable Stoichiometry

Helen Strutt, Jessica Gamage, and David Strutt

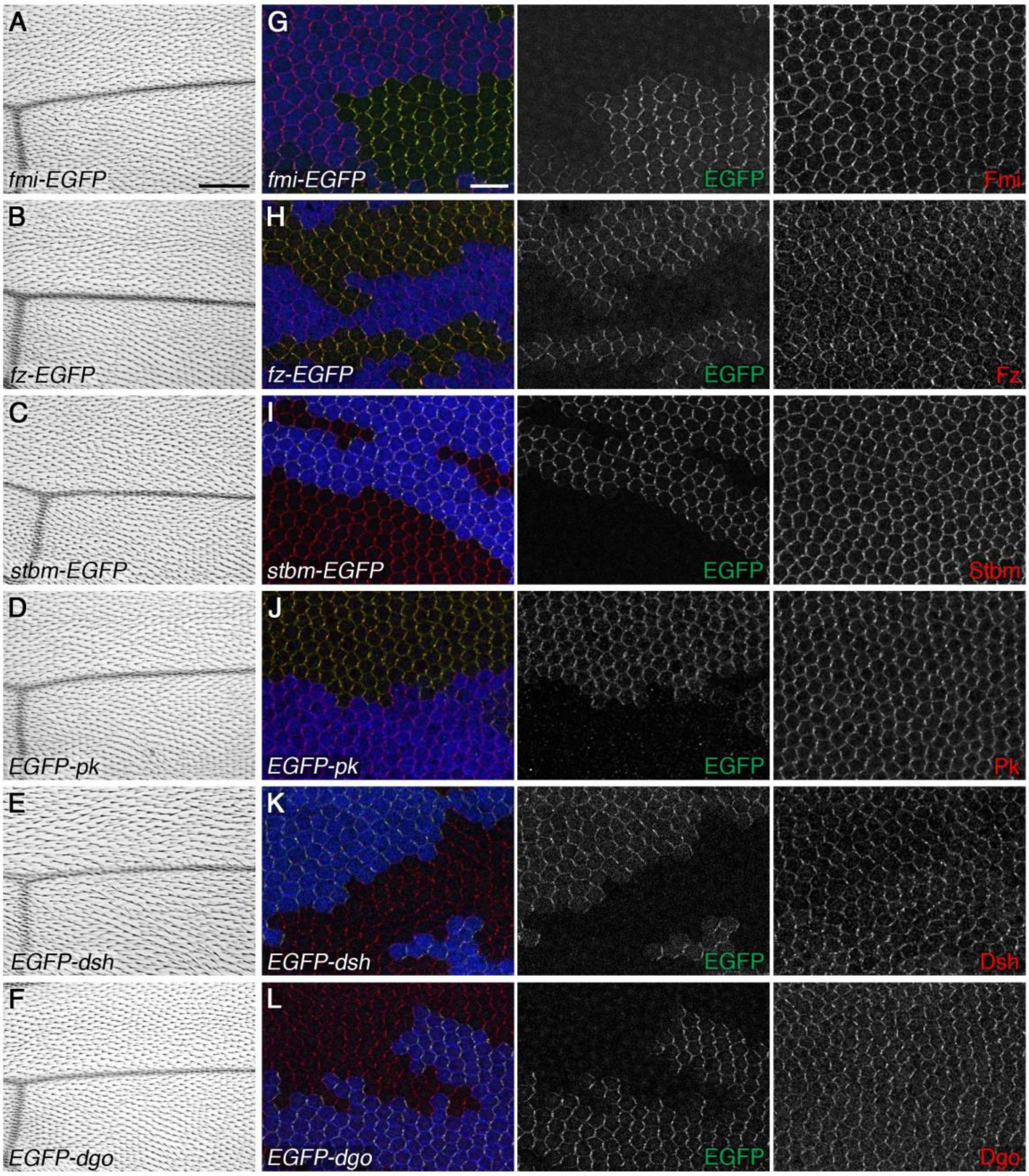

Figure S1. Related to Figure 1. Characterisation of flies expressing EGFP-tagged core proteins.

(A-F) Images of adult wings from flies homozygous for *fmi-EGFP* (A), *fz-EGFP* (B), *P[acman]-stbm-EGFP stbm*^{6} (C), *EGFP-pk* (D), *dsh*^{V26}*; P[acman]-EGFP-dsh* (E) or *P[acman]-EGFP dgo dgo*^{380} (F). Note that the trichomes of EGFP-Dsh are slightly longer than normal, which causes mild mounting artefacts, however polarity is normal. Scale bar is 50 µm.

(G-L) Twin clone experiments, to compare levels of endogenous protein in puncta to levels of tagged protein. (G) *Ubx-FLP; FRT42 fmi-EGFP/FRT42 lacZ*, Fmi staining in red. (H) *Ubx-FLP; fz-EGFP FRT80/arm-lacZ FRT80*, Fz staining in red. (I) *Ubx-FLP; P[acman]-stbm-EGFP arm-lacZ FRT40 stbm*^{6}*/P[acman]-stbm FRT40 stbm*^{6}, Stbm staining in red. (J) *Ubx-FLP; FRT42 EGFP-pk/FRT42 arm-lacZ*, Pk staining in red. (K) *dsh*^{V26}*, Ubx-FLP/dsh*^{V26}*; P[acman]-EGFP-dsh arm-lacZ FRT40/P[acman]-dsh FRT40*, Dsh staining in red. (L) *Ubx-FLP; P[acman]-EGFP-dgo arm-lacZ FRT40 dgo*^{380}*/P[acman]dgo FRT40 dgo*^{380}, Dgo staining in red. Wild-type tissue marked by loss of EGFP (green) and presence of ß-gal staining (blue, G, H, J), or by loss of both EGFP (green) and ß-gal staining (blue, I, K, L). Slightly less Fmi-EGFP localises to junctions than endogenous Fmi (G), whilst more Fz-EGFP than endogenous Fz localises to junctions (H). Slightly less EGFP-Dsh than endogenous Dsh localises to junctions (K), and also less is seen in the cytoplasm. See Table S1 for quantitations. Scale bar is 10 µm.

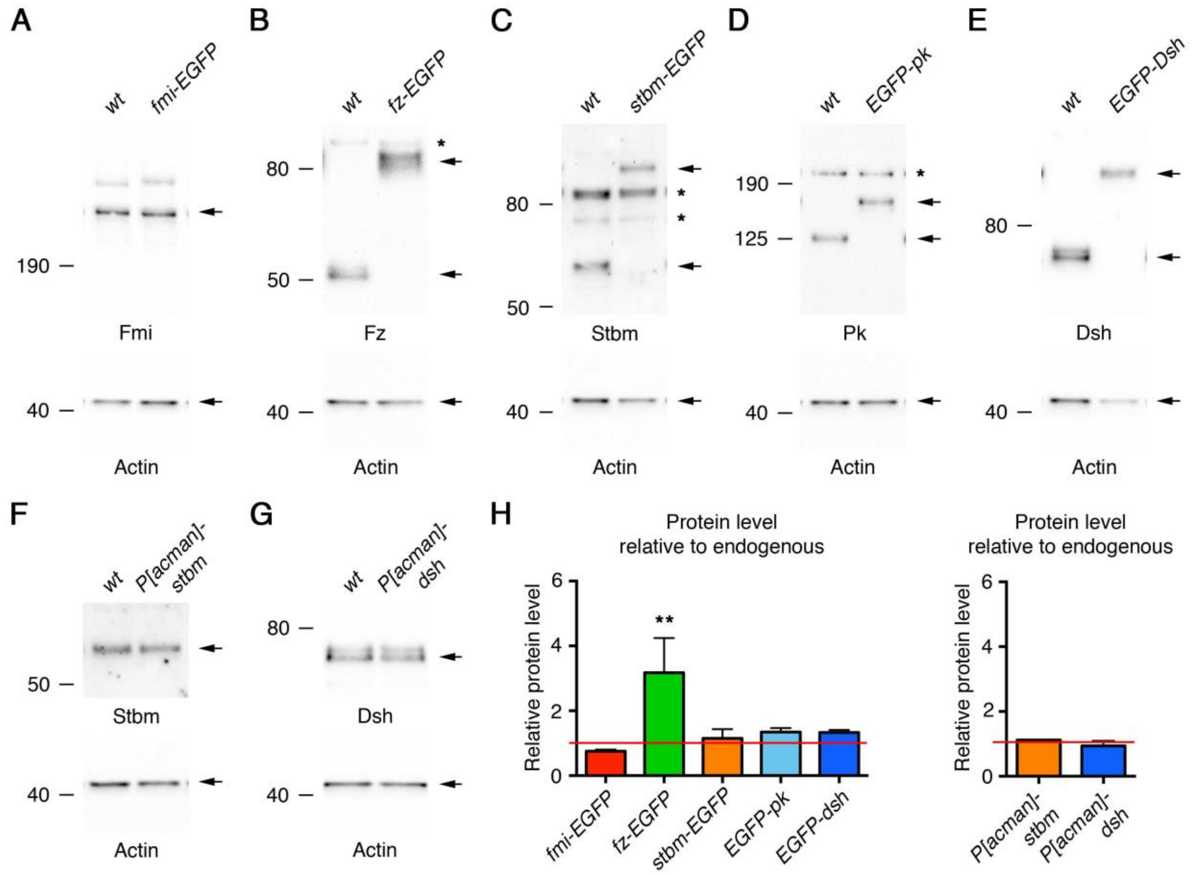

Figure S2. Related to Figure 1. Cellular levels of EGFP-tagged core proteins in pupal wings.

(A-G) Western blots comparing levels of core proteins in pupal wings from wild-type or flies homozygous for *fmi-EGFP* (A), *fz-EGFP* (B), *P[acman]-stbm-EGFP stbm*^{6} (C), *EGFP-pk* (D), *dsh*^{V26}*; P[acman]-EGFP-dsh* (E), *P[acman]-stbm stbm*^{6} (F) or *dsh*^{V26}*; P[acman]-dsh* (G). Arrows indicate specific bands of the expected size, and asterisks mark cross-reacting bands that are still present in samples from null mutants. Addition of EGFP causes an increase in protein size on the gels, except for Fmi-EGFP, where the protein is cleaved into two fragments and an N-terminal antibody is used for detection (Usui et al., 1999, upper band is unprocessed Fmi in panel A).

(H) Quantitation of western blots, from three biological replicates. Protein levels in flies carrying tagged transgenes are shown relative to the amount of endogenous protein in wild-type wings. Error bars are sem, p<0.01** (unpaired t-test). Note that *P[acman]-stbm stbm* and *dsh; P[acman]-dsh* wings show identical levels of expression of Stbm and Dsh as seen in wild-type wings, validating their use as a control in twin clone experiments (Fig. S1).

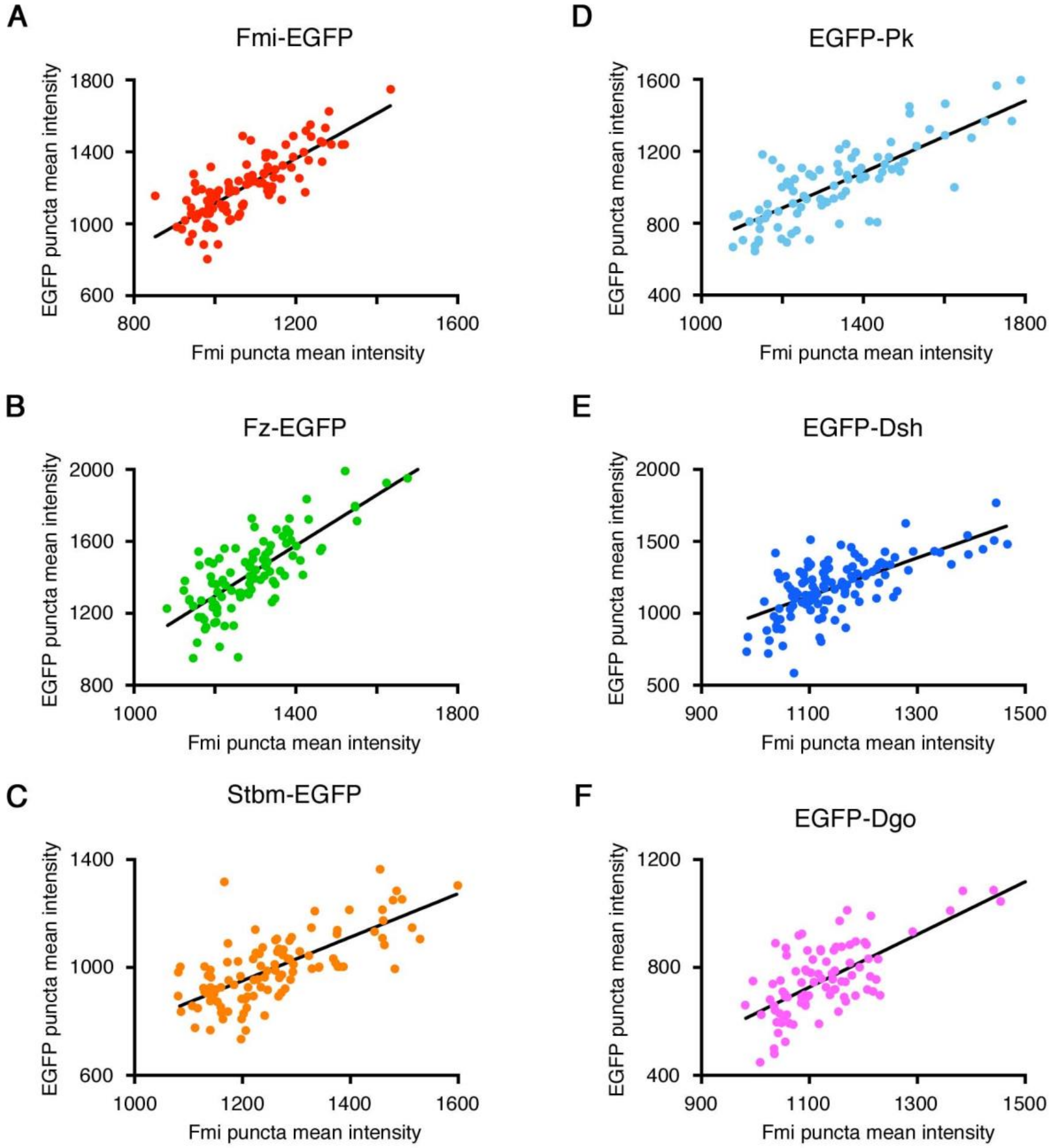

Figure S3. Related to Figure 1. Puncta have a uniform composition.

Pupal wings expressing *fmi-EGFP* (A), *fz-EGFP* (B), *stbm-EGFP* (C), *EGFP-pk* (D), *EGFP-dsh* (E) or *EGFP-dgo* (F) were immunostained for GFP and Fmi. Graphs show data from a single typical image, with intensity of GFP staining in individual puncta plotted against Fmi staining intensity. Units are arbitrary, and different laser intensities were used for each image. For all core proteins, the points fit to a straight line, suggesting that puncta have similar relative levels of each core protein. For *fmi-EGFP* stainings, the GFP and Fmi antibodies recognise the same molecule and R-squared (goodness of fit) is 0.64, suggesting that this is the best fit that can be achieved after taking into account antibody staining noise. For the other stainings shown here, R-squared is 0.61 (*fz-EGFP*), 0.51 (*stbm-EGFP*), 0.66 (*EGFP-pk*), 0.44 (*EGFP-dsh*) and 0.47 (*EGFP-dgo*).

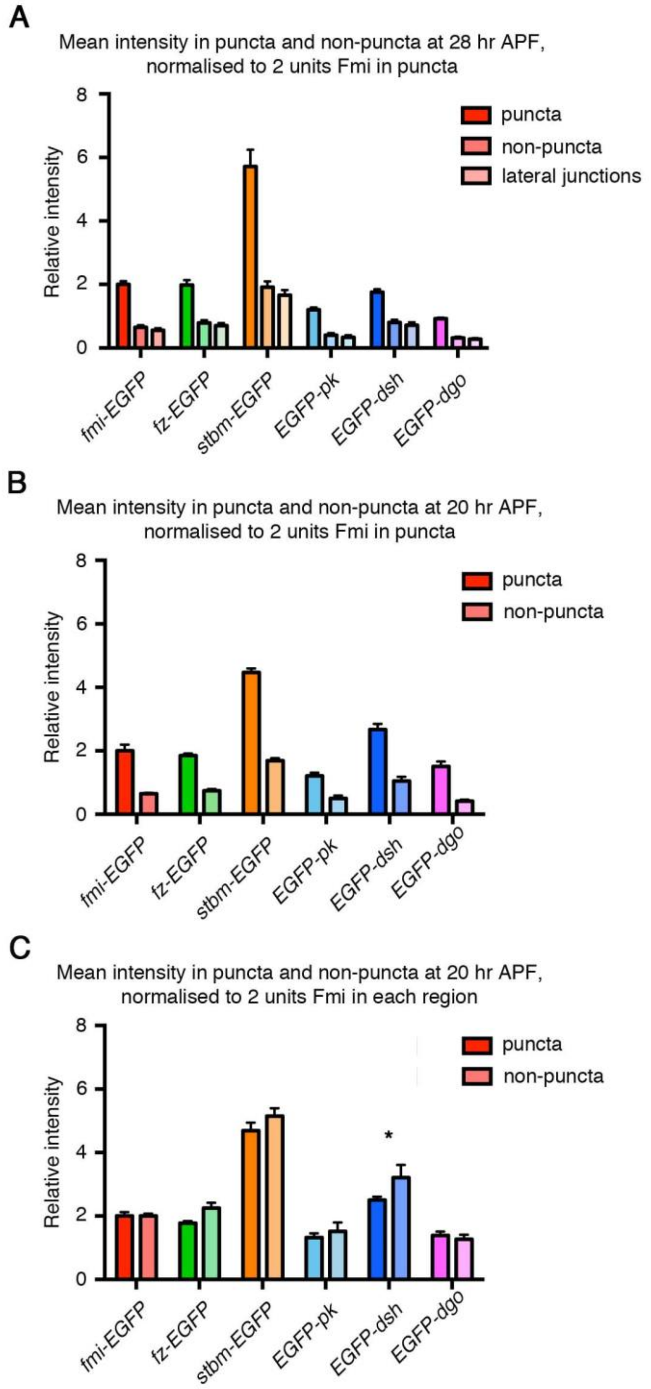

Figure S4. Related to Figure 2. Levels of core proteins in puncta, non-puncta and lateral junctions.

(A) Mean intensity of EGFP fluorescence in puncta, non-puncta and lateral junctions, at 28 hr APF (A) or 20 hr APF (B, C). Data are normalised to 2 units of Fmi-EGFP in puncta (A, B) or to 2 units of Fmi-EGFP in each region (C). (A, B) Puncta are approximately three times as bright as non-puncta at both stages of development. (C) Relative levels of Dsh are significantly increased in non-puncta at 20hr APF. Error bars are sem, $p<0.05$* (2 way ANOVA used to compare puncta and non-puncta). Sample sizes as in Figs. 1E and 2C.

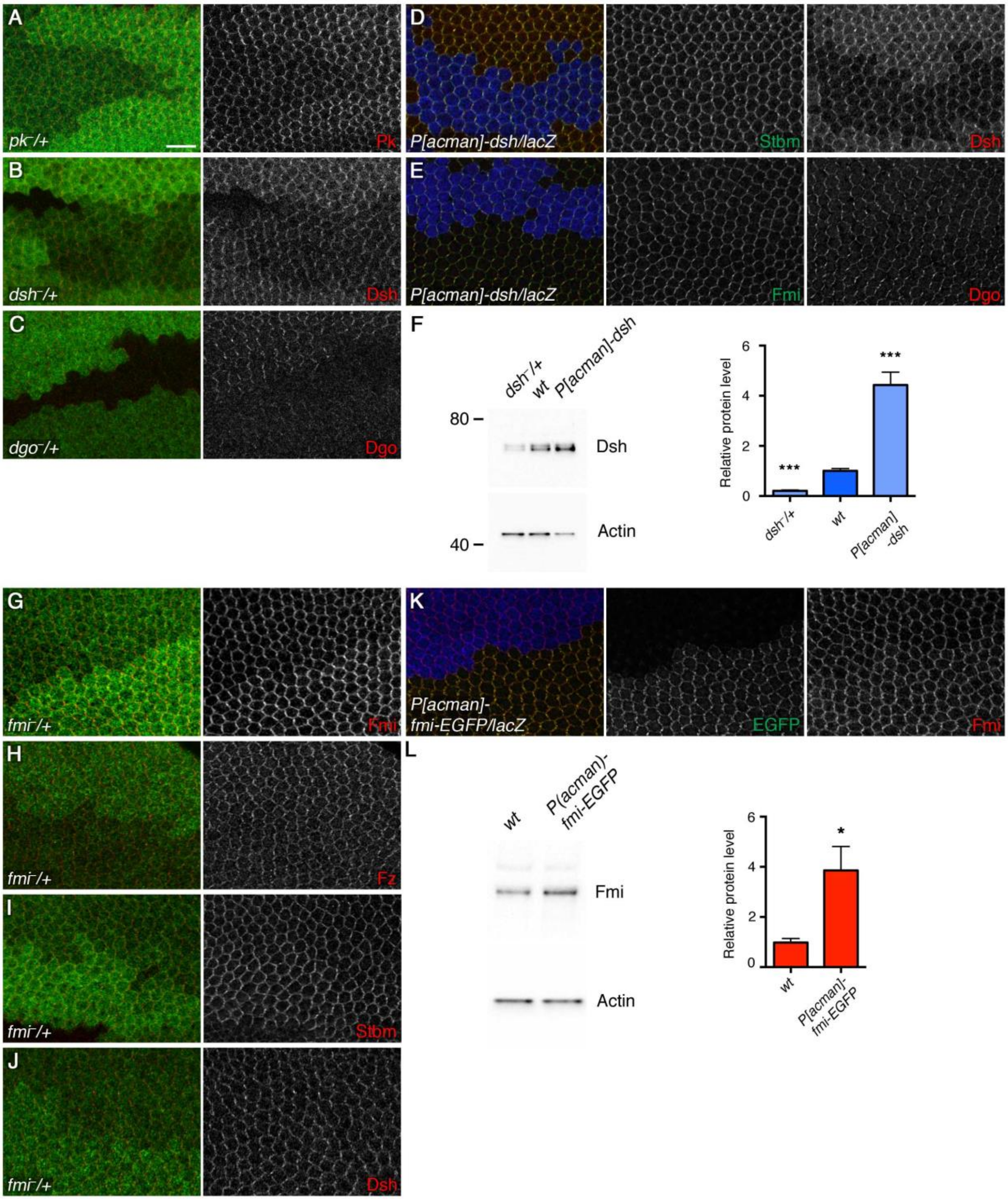

Figure S5. Related to Figures 3 and 4. The effects of decreasing or increasing gene dosage of *prickle*, *dishevelled*, *diego* or *flamingo*.

(A-C) Pupal wings heterozygous for *pk-sple*^{13} (A), *dsh*^{V26} (B) and *dgo*^{380} (C), in which loss of function clones were induced, stained for Pk (red, A), Dsh (red, B) or Dgo (red, C) and ß-gal clonal marker (green). Bright green staining marks twinspot tissue with 2 gene dosages, whilst pale green staining marks heterozygous tissue with 1 gene dosage and absence of green indicates homozygous mutant tissue. In heterozygous tissue, levels of Pk, Dsh and Dgo at junctions are lowered relative to homozygous tissue, indicating that levels of protein in the cell are limiting for incorporation into junctions. Scale bar is 10 µm.

(D, E) Pupal wings with clones of *P[acman]-dsh* (*Ubx-FLP; P[acman]-dsh FRT40/arm-lacZ FRT40*). Tissue staining for the ß-gal clonal marker in blue carries 2 doses of endogenous *dsh* only, whilst tissue lacking the ß-gal clonal marker carries 2 doses of endogenous *dsh* and 2 doses of *P[acman]-dsh*. (D) Wings stained for Stbm

(green) and Dsh (red). (E) Wings stained for Fmi (green) and Dgo (red). Note excess Dsh localising to the cytoplasm, also no increase in Fmi, Stbm or Dgo staining at junctions is seen when 4 doses of *dsh* are present.

(F) Western blots comparing levels of Dsh in pupal wings from dsh^{V26}/+ heterozygous flies, wild-type flies or flies homozygous for *P[acman]-dsh* (carrying two doses of endogenous *dsh* plus 2 doses of *dsh* in the P[acman] construct). Quantitations from three biological replicates show that dsh^{V26}/+ wings have 5-fold less Dsh compared to wild-type (p<0.05*), whilst *P[acman]-dsh* wings have 4.4-fold more Dsh (p<0.001***, ANOVA), possible due to saturation of degradation pathways.

(G-J) Pupal wings heterozygous for fmi^{E59}, in which loss of function clones were induced, stained for ß-gal clonal marker (green) and Fmi (G, red), Fz (H, red), Stbm (I, red) or Dsh (J, red). Bright green staining marks twinspot tissue with 2 gene dosages, whilst pale green staining marks heterozygous tissue with 1 gene dosage and absence of green indicates homozygous mutant tissue. In tissue heterozygous for *fmi*, levels of Fmi and Fz at junctions are lowered, relative to homozygous tissue (G, H). A slight decrease in levels of Stbm is also detectable in tissue heterozygous for *fmi* (I), but no decrease in Dsh is apparent (J).

(K) Pupal wing with clone of *P[acman]-fmi-EGFP* in a wild-type background (*Ubx-FLP; P[acman]-fmi-EGFP FRT40/arm-lacZ FRT40*). Tissue staining for the ß-gal clonal marker (blue) carries 2 doses of endogenous *fmi* only, whilst tissue lacking the ß-gal clonal marker but expressing EGFP (green) carries 2 doses of endogenous *fmi* and 2 doses of *P[acman]-fmi-EGFP*. Wings also stained for Fmi (red). There is only a marginal increase in levels of Fmi at junctions when 4 doses of *fmi* are present.

(L) Western blot comparing levels of Fmi in pupal wings from wild-type flies or flies homozygous for *P[acman]-fmi-EGFP* (carrying two doses of endogenous *fmi* plus 2 doses of *fmi-EGFP* in the P[acman] construct). Quantitations from three biological replicates show a 3.9-fold increase in cellular levels of Fmi in *P[acman]-fmi-EGFP* wings (p<0.05*, unpaired t-test).

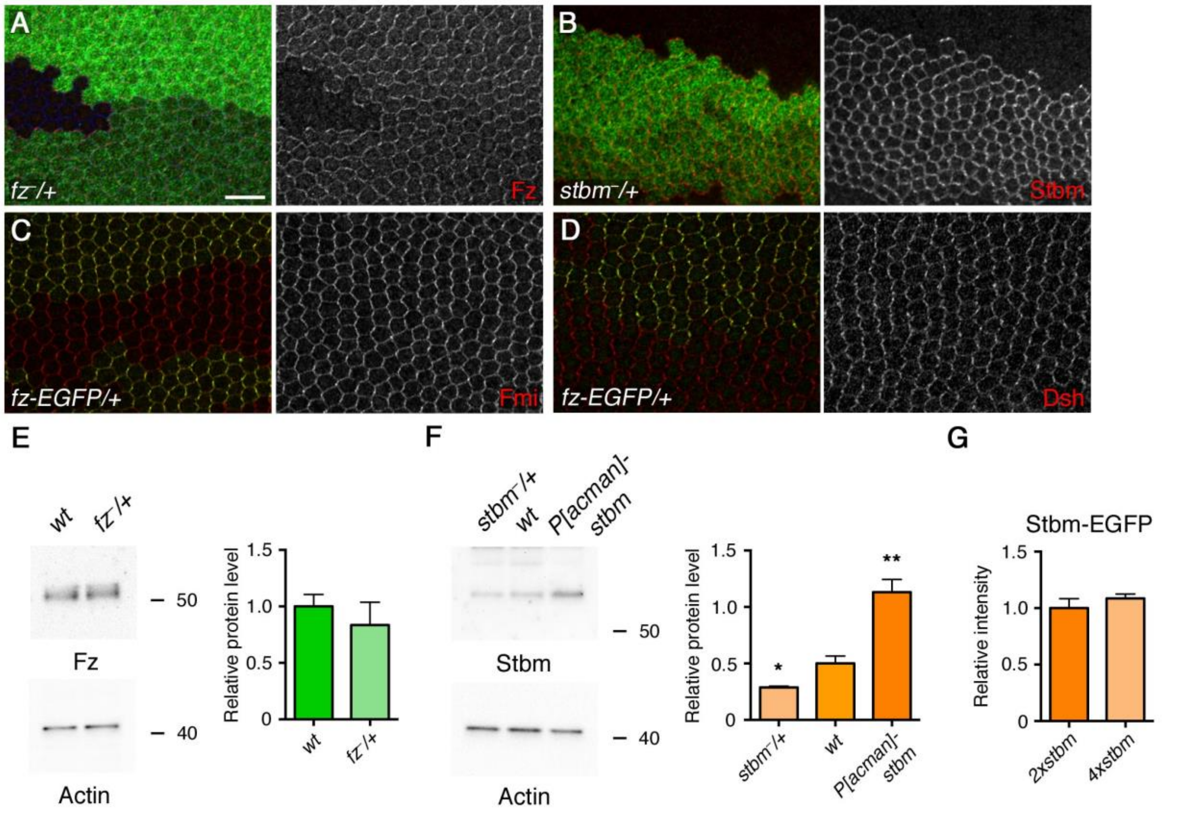

Figure S6. Related to Figure 5. Decreasing *frizzled* and *strabismus* gene dosage.

(A, B) Pupal wings heterozygous for fz^{P21} (A) or $stbm^{6}$ (B), in which loss of function clones were induced, stained for Fz (red, A) or Stbm (red, B) and ß-gal clonal marker (green). Bright green staining marks twinspot tissue with 2 gene dosages, whilst pale green staining marks heterozygous tissue with 1 gene dosage and absence of green indicates homozygous mutant tissue. Halving gene dosage for either *fz* or *stbm* does not cause a reduction in levels of protein in puncta, suggesting that the amount of protein within the cell is not limiting. Note that the amount of Fz in the cytoplasm decreases slightly in *fz* heterozygous tissue. The swirling in Fz localisation is probably due to non-autonomous effects of the neighbouring *fz* mutant tissue. Scale bar is 10 µm.

(C, D) Pupal wings carrying clones of *fz-EGFP*, as in Fig S1H. Tissue expressing Fz-EGFP is marked by green junctional staining, whilst tissue lacking green staining expresses endogenous *fz*. Wings stained for Fmi (C) or Dsh (D) in red. More Fz-EGFP localises to junctions than endogenous Fz (see Fig S1H), but Fmi and Dsh levels do not change.

(E, F) Western blots and quantitations, comparing levels of Fz in pupal wings from fz^{P21}/+ heterozygous flies to wild-type flies (E) or levels of Stbm in pupal wings from $stbm^{6}$/+ heterozygous flies to wild-type flies or flies homozygous for *P[acman]-stbm* (F). Quantitations are from three biological replicates, p<0.01**, p<0.05* (unpaired t-test (E) or ANOVA (F)).

(G) Relative mean intensity of puncta in live pupal wings, comparing flies carrying 1 dose endogenous gene and 1 dose tagged gene with those carrying 2 doses endogenous gene and 2 doses tagged gene. Puncta intensity does not increase when *stbm* dosage is doubled. Error bars are sem, data compared by unpaired t-test, n = 15 (*P[acman-stbm-EGFP/*$stbm^{6}$) , n = 14 (*P[acman]-stbm-EGFP*).

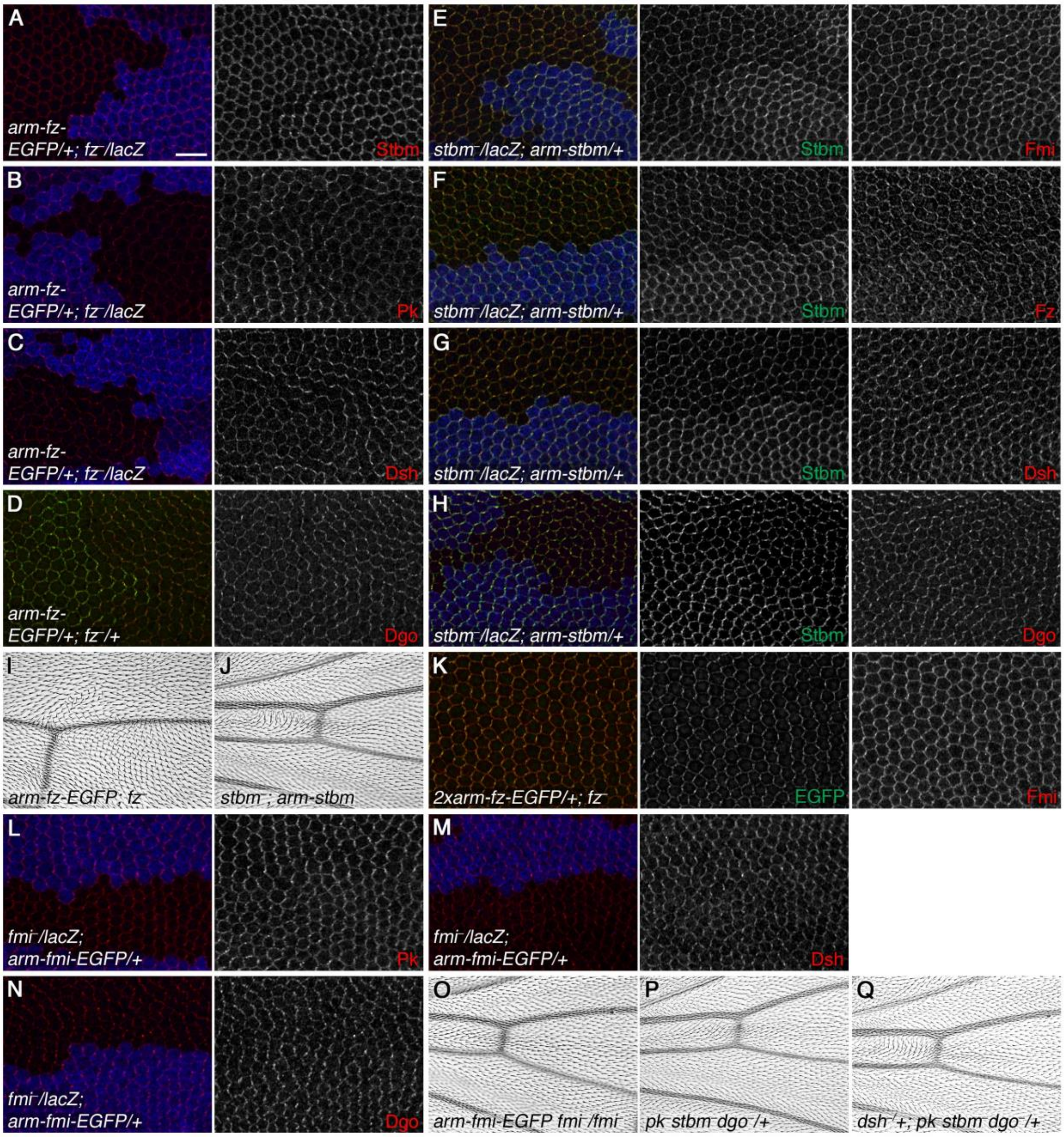

Figure S7. Related to Figures 5 and 6. Wings expressing *arm-frizzled-EGFP, arm-strabismus and arm-flamingo-EGFP*.

(A-D) Pupal wings carrying one copy of *arm-fz-EGFP*, with fz^{P21} clones marked by loss of ß-gal staining (blue, A-C), or by upregulation of EGFP fluorescence (green, D). Wings are labelled for Stbm (A, red), Pk (B, red), Dsh (C, red) or Dgo (D, red). Asymmetric localisation of core proteins is lost in regions with *fz* clones. Levels of Stbm, Pk and Dsh at junctions decrease slightly, whilst levels of Dgo do not decrease. Scale bar is 10 µm.

(E-H) Pupal wings carrying one copy of *arm-stbm*, with $stbm^6$ clones marked by loss of ß-gal staining (blue). Wings stained for Stbm (green), and for Fmi (E, red), Fz (F, red), Dsh (G, red) or Dgo (H, red). Junctional levels of Fmi, Fz, Dsh and Dgo do not appear to alter in regions with *stbm* clones. Asymmetry is reduced.

(I, J) Adult wings of *arm-fz-EGFP/+;* fz^{P21}, imaged on the ventral surface around the posterior cross vein (I) and $stbm^6$*; arm-stbm/+* imaged proximally on the dorsal wing surface (J). Trichome orientation defects are only seen in proximal regions of $stbm^6$*; arm-stbm* wings; this region appears most sensitive to reduction in Stbm levels.

(K) Pupal wings carrying 2 copies of *arm-fz-EGFP* in a fz^{P21} background, showing EGFP fluorescence (green) and stained for Fmi (red). The asymmetry ratio and standard deviation in the cell-by-cell polarity angle were not significantly different to those of wild-type wings (n = 8), in contrast to wings carrying a single copy of *arm-fz-EGFP* (see Fig 5E).

(L-N) Pupal wings carrying one copy of *arm-fmi-EGFP*, with fmi^{E59} clones marked by loss of ß-gal staining (blue). Wings labelled for Pk (L, red), Dsh (M, red) or Dgo (N, red). Junctional levels of Pk and Dgo only decrease very slightly in regions with *fmi* clones, whilst Dsh levels appear unchanged.

(O-Q) Adult wings of *arm-fmi-EGFP* fmi^{E59}/fmi^{E45} (O), $pk\text{-}sple^{13}$, $stbm^{6}$, $dgo^{380}/+$ (P) or $dsh^{V26}/+$; $pk\text{-}sple^{13}$ $stbm^{6}$ $dgo^{380}/+$ (Q), all imaged proximally on the dorsal wing surface. No trichome orientation defects are seen in *arm-fmi-EGFP* fmi^{E59}/fmi^{E45} wings, and only mild defects are seen in proximal regions of $pk\text{-}sple^{13}$, $stbm^{6}$, $dgo^{380}/+$ and $dsh^{V26}/+$; $pk\text{-}sple^{13}$ $stbm^{6}$ $dgo^{380}/+$ wings.

	Fmi-EGFP	Fz-EGFP	Stbm-EGFP	EGFP-Pk	EGFP-Dsh	EGFP-Dgo
Mean puncta intensity compared to untagged protein	0.88<br>sem 0.02<br>***	1.58<br>sem 0.07<br>***	0.99<br>sem 0.03<br>ns	1.16<br>sem 0.02<br>***	0.84<br>sem 0.03<br>***	1.01<br>sem 0.03<br>ns
Mean puncta intensity in X-EGFP/+ compared to X-EGFP/X-EGFP	0.51<br>sem 0.05<br>ns	0.47<br>sem 0.04<br>ns	0.42<br>sem 0.02<br>**	0.49<br>sem 0.02<br>ns	0.51<br>sem 0.04<br>ns	0.60<br>sem 0.04<br>*

Table S1. Related to Figure 1. Quantitation of protein levels in puncta.

Top: Mean intensity of antibody staining in puncta in pupal wings, comparing tissue that is homozygous for tagged genes, to twin clone tissue that is homozygous for endogenous or untagged genes. Genotypes as in Fig S1G-L. Background subtracted was from null clones stained in parallel. The intensities of Fmi-EGFP, EGFP-Pk and EGFP-Dsh puncta are significantly different to those of control puncta, but the difference is less than 20%. p<0.001*** (paired t-test), ns = not significant. n = 16 (Fmi staining of *fmi-EGFP* clones), n = 18 (Fz staining of *fz-EGFP* clones), n = 24 (Stbm staining of *stbm-EGFP* clones), n = 29 (Pk staining of *EGFP-pk* clones), n = 16 (Dsh staining of *EGFP-dsh* clones), n = 13 (Dgo staining of *EGFP-dgo* clones).
Bottom: Relative mean intensity of puncta, comparing live images of wings carrying 1 copy of EGFP-tagged protein and 1 copy of endogenous protein, to wings carrying 2 copies of indicated EGFP-tagged core protein. Note that if EGFP/+ puncta were more than half as bright as the EGFP/EGFP puncta, this might suggest that the EGFP-tagged protein out-competes the wild-type one for inclusion into puncta. Conversely, if the EGFP/+ puncta were less than half as bright as the EGFP/EGFP puncta this might suggest the opposite. Asterisks indicate significant differences from the expected result that intensity of puncta in wings expressing 1 copy of EGFP-tagged protein should be 50% that of puncta in wings expressing 2 copies of EGFP-tagged protein, p<0.05*, p<0.01** (1 sample t-test), ns = not significant. For homozygous EGFP-tagged proteins, sample sizes are as in Fig.1E. n = 12 (*fmi-EGFP/+*), n = 10 (*fz-EGFP/+*), n = 11 (*P[acman]-stbm-EGFP* $stbm^{6}$/+), n = 11 (*EGFP-pk/+*), n = 8 (dsh^{V26}/+; *P[acman]-EGFP-dsh/+*), n = 13 (*P[acman]-EGFP-dgo* dgo^{380}/+).

	**Asymmetry ratio**		**SD of polarity angle (degrees)**	
	wt	X-EGFP	wt	X-EGFP
**Fmi-EGFP**	1.39<br>sem 0.04	1.51<br>sem 0.04<br>*	20.87<br>sem 1.25	20.76<br>sem 1.34<br>ns
**Fz-EGFP**	1.45<br>sem 0.03	1.38<br>sem 0.03<br>*	15.13<br>sem 1.55	18.14<br>sem 1.20<br>ns
**Stbm-EGFP**	1.52<br>sem 0.04	1.39<br>sem 0.03<br>**	15.12<br>sem 1.07	18.52<br>sem 1.08<br>*
**EGFP-Pk**	1.42<br>sem 0.02	1.50<br>sem 0.03<br>**	18.57<br>sem 0.56	16.74<br>sem 0.94<br>ns
**EGFP-Dsh**	1.60<br>sem 0.05	1.62<br>sem 0.06<br>ns	21.36<br>sem 1.33	19.72<br>sem 1.25<br>ns
**EGFP-Dgo**	1.47<br>sem 0.02	1.44<br>sem 0.04<br>ns	16.75<br>sem 0.77	18.78<br>sem 1.20<br>ns

Table S2. Related to Figure 1. Quantitation of asymmetry of EGFP-tagged core proteins.

Asymmetry ratio (left) and standard deviation of polarity angle (right), comparing Fmi staining in tissue that is homozygous for tagged genes, to twin clone tissue that is homozygous for endogenous or untagged genes (wt). Genotypes as in Fig S1G-L. p<0.01**, p<0.05* (paired t-test), ns = not significant. n = 10 (*fmi-EGFP* clones), n = 10 (*fz-EGFP* clones), n = 10 (*stbm-EGFP* clones), n = 12 (*EGFP-pk* clones), n = 8 (*EGFP-dsh* clones), n = 9 (*EGFP-dgo* clones).

		Fmi-EGFP	Fz-EGFP	Stbm-EGFP	EGFP-Pk	EGFP-Dsh	EGFP-Dgo	Asymmetry
***fmi*$^-$/+**	puncta	**0.59**<br>+/- 0.10	0.64<br>+/- 0.07	0.94<br>+/- 0.15	1.06<br>+/- 0.14	0.91<br>+/- 0.08	1.09<br>+/- 0.15	~wt
	non-puncta	**0.49**<br>+/- 0.09	0.65<br>+/- 0.07	0.87<br>+/- 0.13	1.08<br>+/- 0.12	0.92<br>+/- 0.08	1.03<br>+/- 0.18	
***pk-sple*$^-$/+**	puncta	1.02<br>+/- 0.12	0.97<br>+/- 0.06	1.24<br>+/- 0.25	**0.53**<br>+/- 0.10	0.96<br>+/- 0.07	1.06<br>+/- 0.13	~wt
	non-puncta	1.08<br>+/- 0.13	1.04<br>+/- 0.13	1.30<br>+/- 0.28	**0.49**<br>+/- 0.10	1.02<br>+/- 0.07	1.08<br>+/- 0.14	
***dsh*$^-$/**	puncta	0.81<br>+/- 0.04	0.91<br>+/- 0.10	1.02<br>+/- 0.19	0.94<br>+/- 0.06	**0.51**<br>+/- 0.04	1.03<br>+/- 0.10	~wt
	non-puncta	0.85<br>+/- 0.05	1.00<br>+/- 0.10	1.01<br>+/- 0.20	1.00<br>+/- 0.07	**0.44**<br>+/- 0.03	1.01<br>+/- 0.10	
***dgo*$^-$/+**	puncta	0.81<br>+/- 0.10	0.87<br>+/- 0.09	0.93<br>+/- 0.15	1.09<br>+/- 0.14	0.84<br>+/- 0.06	**0.41**<br>+/- 0.04	~wt
	non-puncta	0.83<br>+/- 0.10	0.87<br>+/- 0.10	0.90<br>+/- 0.13	1.11<br>+/- 0.14	0.82<br>+/- 0.05	**0.39**<br>+/- 0.06	
**4 x *fmi***	puncta	**1.19**<br>+/- 0.10	**-**	**-**	**-**	**-**	**-**	-
	non-puncta	**1.21**<br>+/- 0.10	**-**	**-**	**-**	**-**	**-**	
**4 x *stbm***	puncta	**-**	**-**	**1.09**<br>**+/-** 0.08	**-**	**-**	**-**	-
	non-puncta	**-**	**-**	**1.18**<br>+/- 0.11	**-**	**-**	**-**	
**4 x *dsh***	puncta	1.07<br>+/- 0.09	1.05<br>+/- 0.09	**-**	0.94<br>+/- 0.08	**1.43**<br>+/- 0.16	**-**	~wt
	non-puncta	1.08<br>+/- 0.10	0.98<br>+/- 0.08	**-**	0.90<br>+/- 0.06	**1.59**<br>+/- 0.16	**-**	
**4 x *dgo***	puncta	1.12<br>+/- 0.08	0.91<br>+/- 0.06	**-**	0.95<br>+/- 0.08	**-**	**3.03**<br>+/- 0.80	~wt

Table S3. Related to Figures 3 and 4. Summary of stoichiometry data.

Quantitation of puncta and non-puncta intensity in live images of pupal wings from flies expressing EGFP tagged transgenes, relative to controls. Errors are 95% confidence intervals. Bold indicates where the dosage of the tagged gene itself is altered.

	Fmi	Fz	Stbm	Pk	Dsh	Dgo	Asymmetry
arm-fmi-EGFP	**0.59** +/- 0.02	0.85 +/- 0.13	0.69 +/- 0.03	0.94 +/- 0.06	0.98 +/- 0.09	0.91 +/- 0.03	small decrease
arm-fz-EGFP	1.08 +/- 0.11	**0.90** +/- 0.03	0.89 +/- 0.03	0.87 +/- 0.07	0.92 +/- 0.04	0.97 +/- 0.12	strong decrease
arm-stbm	0.97 +/- 0.09	0.99 +/- 0.10	**0.67** +/- 0.02	1.24 +/- 0.10	0.93 +/- 0.42	0.89 +/- 0.06	moderate decrease

Table S4. Related to Figures 5 and 6. Quantitation of immunostaining data for *arm-flamingo-EGFP, arm-frizzled-EGFP* and *arm-strabismus*.

Quantitation of mean membrane intensity of immunostained pupal wings carrying one copy of *arm-fmi-EGFP*, with fmi^{E59} clones (top), one copy of *arm-fz-EGFP*, with fz^{P21} clones (middle), or one copy of *arm-stbm*, with $stbm^{6}$ clones (bottom). Quantitations compare tissue containing mutant clones (only expressing the *arm* transgene) to twin spot tissue without clones (carrying two doses of endogenous gene and one copy of the transgene). Bold indicates where the dosage of the tagged gene itself is altered. Errors are 95% confidence intervals. It was not possible to accurately measure any contribution of antibody staining background. Therefore no background subtraction was used, and the difference in antibody staining between the two regions is likely to be an underestimate. Also note that the antibody staining may not be linear, and the degree of linearity might be different for different antibodies. Fmi antibody staining is relatively lower in tissue expressing the *arm-fmi-EGFP transgene* (0.59 +/- 0.02 compared to wild type tissue) than in *fmi* heterozygous tissue (0.81 +/- 0.08 compared to wild type tissue). The asymmetry ratio decreases strongly for *arm-fz-EGFP* (1.26 in wild-type compared to 0.92 in *fz* mutant tissue), whilst the decrease is less for *arm-stbm* (1.35 in wild-type compared to 1.17 in *stbm* mutant tissue), and there is only a small decrease for *arm-fmi-EGFP* (1.21 for wild-type compared to 1.12 in *fmi* mutant tissue). The asymmetry ratio in the wild-type conditions varies due to differences in antibody staining background (see Supplemental Experimental Procedures).

Supplemental zip file. Related to Experimental Procedures. MATLAB scripts.

MATLAB scripts for measuring mean puncta intensity, mean intensity on proximo-distal versus lateral membranes, and polarity.

Supplemental Experimental Procedures

Molecular biology

For homologous recombination, the sequence for EGFP was inserted into the pRK2 targeting vector (Huang et al., 2008), such that EGFP is upstream of the *LoxP-white-LoxP* cassette. Homology arms of at least 3 kb of genomic DNA for *fz* or *pk* were inserted on either side, such that EGFP is in frame with the open reading frame.

The final exon of *fmi* is spliced into different reading frames for the two isoforms (Chae et al., 1999; Usui et al., 1999). The 3579 amino acid isoform (stan-PA, originally identified by Chae et al., 1999) was shown to be the most abundant in wing discs (Wasserscheid et al., 2007). For homologous recombination, the left homology arm was designed such that the final 10 amino acid exon of this isoform was placed directly downstream of the third (common) exon, and in frame with EGFP, within pRK2. The polyA sequence from SV40 was placed between EGFP and the *LoxP-white-LoxP* cassette, and the right homology arm was from just downstream of exon 3. The splice site needed to make the second isoform (stan-PB) was destroyed by changing the codon usage.

P[acman] constructs (BACPAC resources) were recombineered using standard methods. For N-terminal fusions plasmid PL452-N-EGFP (Addgene) was used as a source vector, whilst for C-terminal fusions a modified version of PL452-C-EGFP (Addgene) was generated, in which the *LoxP*-flanked kanamycin selection cassette site was placed downstream of EGFP rather than upstream. Gene-specific primers were used to amplify EGFP and the selection cassette, in frame with the open reading frame, and the resulting fragment was then recombineered into the relevant P[acman] construct. The kanamycin cassette was then excised, leaving a single LoxP site between *dsh* or *dgo* and the EGFP tag, or downstream of the EGFP tag for *stbm*. The design for *P[acman]-fmi-EGFP* was similar to that of the homologous recombination construct, with the final coding exon of stan-PA in frame with EGFP, followed by the polyA sequence from SV40.

Fly stocks and genetics

Fly stocks are described in FlyBase. FlyBase IDs are *FlyBase: FBgn0024836* (*fmi/starry night*), *FlyBase: FBgn0001085* (*fz*), *FlyBase: FBgn0015838* (*stbm/Vang Gogh*), *FlyBase: FBgn0003090* (*pk*), *FlyBase: FBgn0000499* (*dsh*) and *FlyBase: FBgn0086898* (*dgo*). fmi^{E59}, fz^{P21}, $stbm^{6}$, $pk\text{-}sple^{13}$, dsh^{V26} and dgo^{380} are null alleles. *P[acman]* constructs were integrated into the genome via ΦC31-mediated recombination into the *attP40* landing site. *P[acman]-stbm-EGFP* and *P[acman]-EGFP-dgo* were then recombined with $stbm^{6}$ or dgo^{380}, respectively, whilst *P[acman]-EGFP-dsh* was crossed into a dsh^{V26} mutant background. To avoid dosage compensation effects, females of *P[acman]-EGFP-dsh* were used for imaging.

For homologous recombination, the targeting vectors were introduced into the genome by P-element mediated transgenesis, to produce donor strains. Homologous recombination was carried out as described by Huang et al (2008). Targeted lines on the correct chromosome were verified by PCR of EGFP, and the *white* marker gene was excised by Cre-Lox recombination, leaving a single *LoxP* site downstream of EGFP.

Transgenes used were *armP-fz-EGFP* (Strutt, 2001) and *armP-fmi-EGFP* (Strutt et al., 2011). *armP-stbm* is full-length Stbm coding sequence under control of the *arm* promoter. Mitotic clones were induced using the FLP/FRT system and *Ubx-FLP*, except for when heterozygous tissue was needed, in which case *hs-FLP* was used.

Transgenics were generated by Genetivision and Bestgene.

Full genotypes for images are:

Figure 1

(E) *fmi-EGFP – fz-EGFP – P[acman]-stbm-EGFP* $stbm^{6}$ *– EGFP-pk –* dsh^{V26}*; P[acman]-EGFP-dsh – P[acman]-EGFP-dgo* dgo^{380}

(G) *Ubx-FLP; fz-EGFP FRT80/FRT80*

(H) *Ubx-FLP; P[acman]-stbm-EGFP FRT40* $stbm^{6}$*/P[acman]-stbm FRT40* $stbm^{6}$

(I) *Ubx-FLP; FRT42 fmi-EGFP/FRT42*

Figure 2

(A,C,D) As Fig 1(E)

(B) *fmi-EGFP/+ – fz-EGFP/+ – P[acman]-stbm-EGFP* $stbm^{6}$*/+ – EGFP-pk/+ –* dsh^{V26}*/+; P[acman]-EGFP-dsh/+ – P[acman]-EGFP-dgo* dgo^{380}*/+*

Figure 3

(A) *EGFP-pk – EGFP-pk/pk-sple*^{13}

(B) *dsh*^{V26}*; P[acman]-EGFP-dsh – dsh*^{V26}*; P[acman}-EGFP-dsh/+*

(C) *P[acman]-EGFP-dgo dgo*^{380} *– P[acman]-EGFP-dgo dgo*^{380}*/dgo*^{380}

(D,O) *fmi-EGFP/+ – fmi-EGFP/pk-sple*^{13} *– dsh*^{V26}*/+; fmi-EGFP/+ – fmi-EGFP/dgo*^{380}

(E) *fz-EGFP/+ – pk-sple*^{13}*/+; fz-EGFP/+ – dsh*^{V26}*/+; fz-EGFP/+ – dgo*^{380}*/+; fz-EGFP/+*

(F) *P[acman]-stbm-EGFP stbm*^{6}*/+ – P[acman]-stbm-EGFP stbm*^{6}*/pk-sple*^{13} *– dsh*^{V26}*/+; P[acman]-stbm-EGFP stbm*^{6}*/+ – P[acman]-stbm-EGFP stbm*^{6}*/dgo*^{380}

(G) *EGFP-pk/+ – dsh*^{V26}*/+; EGFP-pk/+ – EGFP-pk/dgo*^{380}

(H) *dsh*^{V26}*/+; P[acman]-EGFP-dsh/+ – dsh*^{V26}*/+ ; P[acman]-EGFP-dsh/pk-sple*^{13} *– dsh*^{V26}*/+ ; P[acman]-EGFP-dsh/dgo*^{380}

(I) *P[acman]-EGFP-dgo dgo*^{380}*/+ – P[acman]-EGFP-dgo dgo*^{380}*/pk-sple*^{13} *– dsh*^{V26}*/+; P[acman]-EGFP-dgo dgo*^{380}*/+*

(J) *dsh*^{V26}*/+; P[acman]-EGFP-dsh/+ – P[acman]-EGFP-dsh*

(K) *P[acman]-EGFP-dgo dgo*^{380}*/+ – P[acman]-EGFP-dgo*

(L,P) *fmi-EGFP/+ – P[acman]-dsh fmi-EGFP/P[acman]-dsh – P[acman]-dgo fmi-EGFP/P[acman]-dgo*

(M) *fz-EGFP/+ – P[acman]-dsh; fz-EGFP/+ – P[acman]-dgo; fz-EGFP/+*

(N) *EGFP-pk/+ – P[acman]-dsh EGFP-pk/P[acman]-dsh – P[acman]-dgo EGFP-pk/P[acman]-dgo*

Figure 4

(A,H) *fmi-EGFP – fmi-EGFP/fmi*^{E59}

(B,I) *fz-EGFP/+ – fmi*^{E59}*/+; fz-EGFP/+*

(C,G,J) *P[acman]-stbm-EGFP stbm*^{6}*/+ – P[acman]-stbm-EGFP stbm*^{6}*/fmi*^{E59}

(D,K) *EGFP-pk/+ – EGFP-pk/fmi*^{E59}

(E,L) *dsh*^{V26}*/+; P[acman]-EGFP-dsh/+ – dsh*^{V26}*/+; P[acman]-EGFP-dsh/fmi*^{E59}

(F,M) *P[acman]-EGFP-dgo dgo*^{380}*/+ – P[acman]-EGFP-dgo dgo*^{380}*/fmi*^{E59}

Figure 5

(A,B,E) *arm-fz-EGFP/+; fz*^{P21} *FRT80/arm-lacZ FRT80*

(C,D,F) *FRT42 stbm*^{6}*/FRT42 arm-lacZ; arm-stbm/+*

Figure 6

(A-D) *FRT42 fmi*^{E59}*/FRT42 arm-lacZ; arm-fmi-EGFP/+*

(E,F) *fmi-EGFP/+ – fmi-EGFP/pk-sple*^{13} *stbm*^{6} *dgo*^{380} *– dsh*^{V26}*/+; fmi-EGFP/pk-sple*^{13} *stbm*^{6} *dgo*^{380}

Immunostaining and antibodies

Pupal wings were dissected at 28 hr after puparium formation (APF) at 25°C as previously described (Strutt, 2001). Primary antibodies for immunostaining were mouse monoclonal anti-Fmi 74 (DSHB, Usui et al., 1999), affinity-purified rabbit anti-Fz (Bastock and Strutt, 2007), rabbit anti-Stbm (Warrington et al., 2013), rat anti-Stbm (Strutt and Strutt, 2008), affinity-purified rat anti-Pk (Strutt et al., 2013), rat anti-Dsh (Strutt et al., 2006), mouse monoclonal anti-βgal (Promega), rabbit anti-βgal (Cappel) and rabbit anti-GFP (Abcam, affinity-purified). Anti-Dgo is a rat antibody directed against amino acids 671-927, and affinity purified with a fusion protein against amino acids 671-780.

Westerns were probed with mouse monoclonal anti-Fmi 74 (DSHB, Usui et al., 1999), affinity-purified rabbit anti-Fz (Bastock and Strutt, 2007), rabbit anti-Stbm (Rawls and Wolff, 2003), affinity-purified rat anti-Pk (Strutt et al., 2013), affinity-purified rabbit anti-Dsh (Strutt et al., 2006) and Actin AC-40 mouse monoclonal (Sigma). We do not have an anti-Dgo antibody suitable for western blotting.

Imaging

Both live and fixed pupal wings were imaged on a Nikon A1R GaAsP confocal microscope using a 60x NA1.4 apochromatic lens, giving a pixel size of 70 nm, and the pinhole was set to 1.2 AU. For fixed samples, 9 Z-slices separated by 150 nm were imaged, and then the 3 brightest slices around puncta were selected and averaged for each channel in ImageJ. For live imaging, a single slice was taken, and the laser power at the lens was monitored to ensure that samples were imaged at constant laser power. Imaging of EGFP solution demonstrated that detection varied linearly with concentration over a wide range of intensities.

For FRAP analysis, images were 256 x 256 pixels, with a pixel size of 100 nm, and a pinhole of 1.2 AU. ROIs of about 2 μm^2 were selected for puncta and non-puncta. Three pre-bleach images were taken, and ROIs were then bleached using a 488 nm Argon laser at 100% with 8 passes (1 sec total time). Immediately following bleaching, 5 images were taken at 5 sec intervals, followed by 10 images at 10 sec intervals, 10 images at 15 sec intervals and 8 images at 30 sec intervals.

Puncta detection and quantitation

For puncta detection on live images, out of focus regions were cropped out and a membrane mask was generated using Packing Analyzer (Aigouy et al., 2010). The mask from Packing Analyzer was dilated and eroded to smooth its edges, before dilating to a final radius of 8 pixels (560 nm). Puncta detection and quantitation was carried out using a MATLAB script (Mathworks). To improve puncta detection, the intensity histogram of the image was stretched so that the top 0.1% and bottom 0.1% of data were saturated. Noise was removed by applying a Gaussian filter followed by a median filter (Supplemental Experimental Procedures Fig 1).

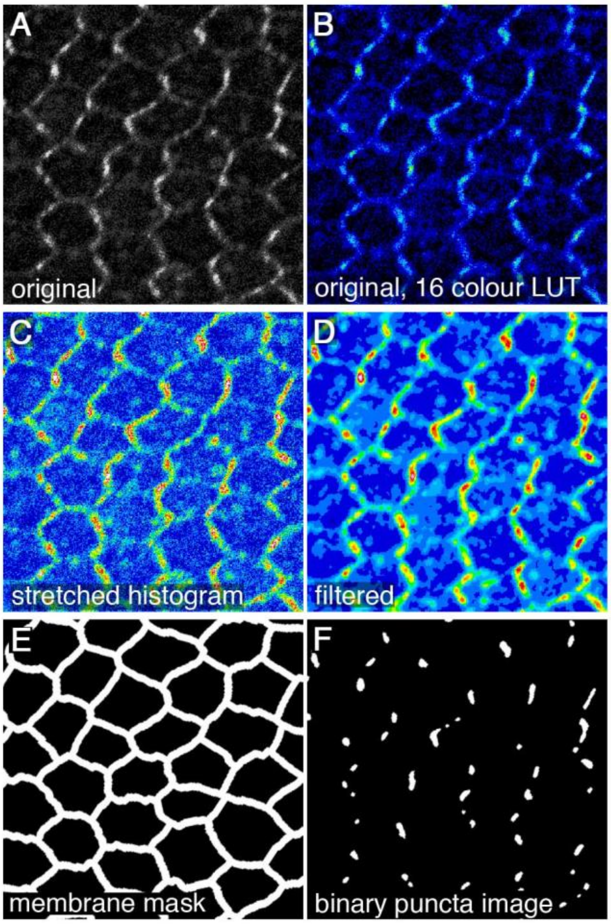

Supplemental Experimental Procedures Fig 1. Detecting puncta.

(A, B) An original live image from a pupal wing expressing *fz-EGFP*, in greyscale (A) or with a 16 colour lookup table (B). (C, D) The same image after stretching the histogram so that the top 0.1% and bottom 0.1% of data are saturated (C), and after applying a Gaussian filter followed by a median filter to reduce noise (D). (E) The membrane mask after being dilated and eroded to smooth its edges, then re-dilated to a radius of 4 pixels. (F) A binary image of detected puncta.

Puncta were detected in the stretched/filtered image using the bwconncomp function of MATLAB, and objects smaller than 9 pixels were then excluded. A simple threshold value for object recognition could not be used, as different genotypes have different overall intensities, and images are different distances from the coverslip (see below). Therefore we made the assumption that puncta in different genotypes would have the same average size, and used MATLAB to find a threshold value for each image whereby puncta occupied 10% of the total area of the junctions. By eye, this appeared to select all the large puncta. The regionprops function was then applied to the original, unstretched, unfiltered image, to measure mean puncta intensity, number and area. The mean intensity of membranes and cytoplasm was also determined. When gene dosage was altered, puncta intensity varied similarly to overall membrane intensity, suggesting that puncta are not significantly larger or smaller, and validating the use of a fixed membrane proportion to define puncta. Increasing or decreasing the area defined as puncta did not significantly alter the relative puncta intensities.

Background due to autofluorescence was determined by imaging wings containing clones of *EGFP-pk*. This value was very low, typically half the value of the laser off background, and was subtracted. After correcting for wing-coverslip distance (see below), the mean puncta intensity was averaged for each wing (1-3 images were taken per wing, depending on the flatness). At least 8 wings were imaged for most genotypes: an initial dataset was used to do a power calculation using G*Power, which suggested that this would be sufficient to distinguish between differences of intensities of 10:7, with a power of 0.8. Mean puncta intensity was then averaged across wings. Unpaired t-tests were used for pair-wise comparisons, and for multiple comparisons, ANOVA with Tukey's test was used to compare all genotypes or with Dunnett's test to compare to a control genotype. For comparing multiple genotypes at different stages or in puncta and non-puncta, 2-way ANOVA with Holm-Šídák correction was used.

For quantitating puncta in clones in fixed images, wild-type and mutant regions were separated, and puncta detection applied to each region separately. Due to antibody staining background and possible non-linearity of staining, measured puncta intensity in wild-type and mutant tissue is unlikely to scale linearly with real protein amounts; however it can be determined whether puncta intensities in mutant tissue are significantly higher or lower than in control tissue. For twin clone experiments comparing tagged to untagged protein (Fig S1 and Table S1), wings containing mutant clones were stained in parallel, and the intensity within the clone was subtracted as background on the twin clone images, leading to more accurate values. Puncta intensity was compared between control and mutant regions in the same wing using paired t-tests.

For measuring puncta asymmetry on the borders of *fmi-EGFP*, *fz-EGFP* and *stbm-EGFP* twin clones, puncta were detected over the whole image on the basis of Fmi staining. Puncta on proximal and distal cell ends were selected manually in ImageJ, and mean intensity and puncta area were measured for Fmi, Fz or Stbm. Proximal and distal puncta intensity were averaged per wing, and compared in the same wing using paired t-tests. For clones of *fz-EGFP* and *stbm-EGFP*, mean puncta area was similar for proximal and distal puncta.

Correcting for wing-coverslip distance

Pupal wings sit inside an impermeable membrane, and during live imaging the distance of the wing from the coverslip varies between wings and between different regions of the same wing. By plotting puncta intensity of different wings of the same genotype against distance from the coverslip, puncta intensity was shown to decrease exponentially with distance from the coverslip according to the light attenuation equation $I = I_0 * \exp(-\alpha * X)$, where I is the light at X distance from the coverslip, I_0 is intensity at the coverslip and α is the attenuation coefficient (Supplemental Experimental Procedures Fig 2). To determine the attenuation coefficient for 28 hr APF pupal wing fluid, an initial dataset of about 20 images from each of 25 genotypes was collected. Prism was used to fit curves for each genotype, assuming that the attenuation coefficient was the same for all genotypes, and the best-fit value was extracted (α=0.0186). This value was then used in subsequent experiments to extrapolate puncta intensity to I_0. The attenuation coefficient was calculated separately for 20 hr APF pupal wings, where the dorsal and ventral wing epithelia are not yet apposed, and the apical wing surface is generally closer to the pupal wing membrane (α=0.0317).

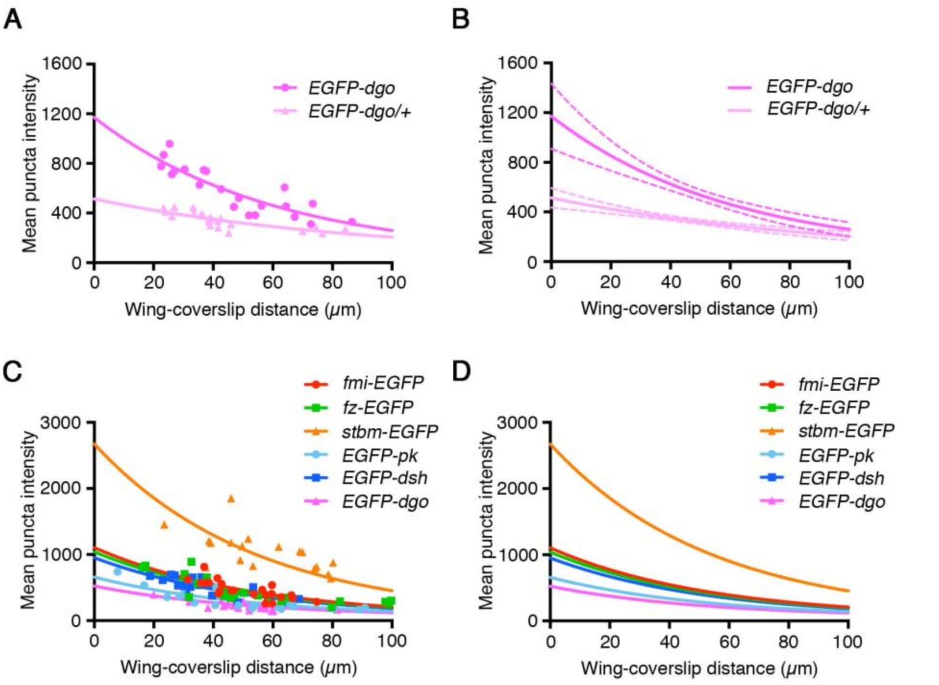

Supplemental Experimental Procedures Fig 2. Correcting for wing-coverslip distance.

(A, B) Mean puncta intensity in live images of pupal wings from *P[acman]-EGFP-dgo dgo*^{380} and *P[acman]-EGFP-dgo dgo*^{380}/+, plotted against distance between the wing and the coverslip. (A) Individual points fit to one-phase exponential decay curves. (B) Exponential decay curves with 95% confidence intervals (dashed lines).

(C, D) Mean puncta intensity in the live images of pupal wings used for stoichiometry in Fig. 1E, plotted against wing-coverslip distance. The exponential decay curve was fitted using an experimentally-determined attenuation coefficient (see Materials and Methods), shown with the individual data points (C) or without (D).

Polarity measurement

To determine polarity using MATLAB, image intensity histograms were stretched and membrane masks were dilated and eroded as for puncta detection. An inverse cytoplasmic mask was generated and each cell identified as an object using bwconncomp. For each cell, the centroid was identified and the perimeter extracted. For each point on the perimeter of a cell a circular mask of 3 pixels was placed and the average pixel intensities and the angle from the centroid was determined. Cell outlines were approximated to a circle, and data placed into 360 bins, depending on their angle. The intensity data in groups of 90 bins were compared, to identify the angles at which the maximum asymmetry was observed (e.g. intensity in bins 0-90 plus 280-270, compared to bins 90-180 plus 270-360). This gave a mean polarity and angle for each cell; this vector polarity was then averaged for all cells in the image, to give a mean vector polarity (asymmetry ratio on plots). The standard deviation in the cell-by-cell polarity angle was used as a measure of the coordination in polarity between cells.

For live images laser off background and autofluorescent background were subtracted prior to polarity measurement. Averaging between wings and statistical tests were similar to those for puncta detection.

For fixed wings, laser off background was subtracted, but an unknown amount of antibody staining background was present in each image, so the resulting degree of asymmetry tended to be lower than for live images. Polarity values were compared between control and mutant regions in the same wing using paired t-tests.

Measuring intensity on lateral junctions

To measure intensity on proximal-distal and lateral junctions, a variant on the MATLAB script for polarity measurement was used. After identifying each cell, the centroid was determined. The cytoplasm was dilated by 8 pixels and the original cytoplasm mask subtracted to give a membrane mask for each cell. The intensity of each pixel within this mask on the original image was determined, together with its angle from the centroid. The 90° bins giving maximum asymmetry for each cell were then determined as for polarity measurement, and these intensities were assigned as proximal-distal junctions, whilst the intensities in orthogonal bins were assigned as lateral junctions.

Supplemental References

Aigouy, B., Farhadifar, R., Staple, D.B., Sagner, A., Röper, J.-C., Julicher, F., and Eaton, S. (2010). Cell flow reorients the axis of planar polarity in the wing epithelium of *Drosophila*. Cell *142*, 773-786.

Bastock, R., and Strutt, D. (2007). The planar polarity pathway promotes coordinated cell migration during *Drosophila* oogenesis. Development *134*, 3055-3064.

Chae, J., Kim, M.J., Goo, J.H., Collier, S., Gubb, D., Charlton, J., Adler, P.N., and Park, W.J. (1999). The *Drosophila* tissue polarity gene *starry night* encodes a member of the protocadherin family. Development *126*, 5421-5429.

Huang, J., Zhou, W., Watson, A.M., Jan, Y.N., and Hong, Y. (2008). Efficient ends-out gene targeting in *Drosophila*. Genetics *180*, 703-707.

Rawls, A.S., and Wolff, T. (2003). Strabismus requires Flamingo and Prickle function to regulate tissue polarity in the *Drosophila* eye. Development *130*, 1877-1887.

Strutt, D.I. (2001). Asymmetric localisation of Frizzled and the establishment of cell polarity in the *Drosophila* wing. Mol Cell *7*, 367-375.

Strutt, H., Price, M.A., and Strutt, D. (2006). Planar polarity is positively regulated by casein kinase Iε in *Drosophila*. Curr Biol *16*, 1329-1336.

Strutt, H., and Strutt, D. (2008). Differential stability of Flamingo protein complexes underlies the establishment of planar polarity. Curr Biol *18*, 1555-1564.

Strutt, H., Thomas-MacArthur, V., and Strutt, D. (2013). Strabismus promotes recruitment and degradation of farnesylated Prickle in *Drosophila melanogaster* planar polarity specification. PloS Genetics *9*, e1003654.

Strutt, H., Warrington, S.J., and Strutt, D. (2011). Dynamics of core planar polarity protein turnover and stable assembly into discrete membrane subdomains. Dev Cell *20*, 511-525.

Usui, T., Shima, Y., Shimada, Y., Hirano, S., Burgess, R.W., Schwarz, T.L., Takeichi, M., and Uemura, T. (1999). Flamingo, a seven-pass transmembrane cadherin, regulates planar cell polarity under the control of Frizzled. Cell *98*, 585-595.

Warrington, S.J., Strutt, H., and Strutt, D. (2013). The Frizzled-dependent planar polarity pathway locally promotes E-cadherin turnover via recruitment of RhoGEF2. Development *140*, 1045-1054.

Wasserscheid, I., Thomas, U., and Knust, E. (2007). Isoform-specific interaction of Flamingo/Starry Night with excess Bazooka affects planar cell polarity in the *Drosophila* wing. Dev Dynamics *236*, 1064-1071.
