# Supplementary material for: Robust Asymmetric Localization of Planar Polarity Proteins Is Associated with Organization into Signalosome-like Domains of Variable Stoichiometry
Source: Cell Rep. 2016 Dec 6;17(10):2660–71. doi: 10.1016/j.celrep.2016.11.021 (PMC5177602; doi:10.1016/j.celrep.2016.11.021)
Supplement: Data S1. MATLAB Scripts, Related to Experimental Procedures — MATLAB scripts for measuring mean puncta intensity, mean intensity on proximo-distal versus lateral membranes, and polarity. [file mmc2.zip › MATLAB scripts/Notes on MATLAB scripts.doc]

**MATLAB scripts**

Robust Asymmetric Localisation of Planar Polarity Proteins Is Associated with Organisation into Signalosome-like Domains of Variable Stoichiometry

Helen Strutt, Jessica Gamage and David Strutt

Cell Reports (2016)

**Puncta_defined_area**

This script is suitable for measuring puncta intensities of different fluorescent proteins, where puncta are expected to be the same size (eg in wild-type tissue).

This script offers the choice of detecting puncta in the entire image, or only in membranes. To detect puncta only in membranes, a membrane mask must also be imported (see below). The radius of the membrane mask in pixels is user-defined, dependent on the resolution of the image.

The script searches for a threshold such that a defined proportion of the membrane or cell is puncta. Puncta have a minimum size (defined in a dialogue box). The proportion of membrane or cell that is puncta can be defined in a dialogue box. For pupal wings, defining 10% of membranes (or 2% of the cell) as puncta selects most visible puncta. The script searches for the defined threshold until a certain (user-defined) tolerance is achieved. We suggest setting tolerance as 1% (eg threshold will be between 9.9 - 10.1). If a threshold in this range is not achieved within 100 loops, the script will proceed with the closest threshold found.

The final mask, and the binary thresholded image are saved to the image folder. A csv file is also saved to the image folder. This lists the input parameters, and the results:-

Parea, Pareastd (Mean puncta area and standard deviation)

Pmean, Pmeanstd (Mean puncta intensity and standard deviation)

Pmax, Pmaxstd (Maximum puncta intensity and standard deviation)

Pmin, Pminstd (Minimum puncta intensity and standard deviation)

Pn (Number of puncta detected)

Carea and Careastd (Mean cytoplasm area and standard deviation)

Cmean, Cmeanstd, Cmax, Cmaxstd, Cmin, Cminstd (cytoplasm mean, maximum and minimum)

Cn (number of cytoplasm areas = number of cells)

Marea (Membrane area)

Mmean, Mmax and Mmin (membrane mean, maximum and minimum)

Mmean-puncta (membrane mean, excluding puncta)

**Polarity**

This script is for measuring asymmetric localisation. A membrane mask must also be imported (see below). The radius of the membrane mask is set at 3 pixels, but this may need to be altered, depending on the resolution of the image.

Saving cell-by-cell angle data for each image, rose plots and nematics is optional.

The output file contains:-

Mean polarity and polarity std (average polarity magnitude and standard deviation)

Mean angle and angle std (average polarity angle and standard deviation)

Mean vector angle

Mean vector polarity

**PD_vs_lateral_intensity**

This script is a variant of the polarity script, for comparing intensities of proximal-distal versus lateral junctions. Proximal-distal junctions are defined as those that give maximum asymmetry.

Polarity data is also included in the output.

**Data formats and organisation**

All scripts expects to open a folder of images, which is located using a pop-up dialogue box. Images can be 8-bit or 16-bit, but must be single colour, single images (not a stack or multichannel image).

For the *Puncta_defined_area* script, images can be in any format opened by the bfopen function. The type of image (eg .tif) is specified in a pop-up dialogue box.

For the *Polarity* and *PD_vs_lateral_intensity* scripts, images should be in .tif format.

**Membrane masks**

Membrane masks are made in Packing Analyzer. Packing Analyszer creates a folder with the same name as the image (minus the file extension). The membrane mask is the file named "handCorrection.png". The folder containing the mask must be inside the folder of images.

**Additional scripts**

Bio-formats MATLAB toolbox: required for *Puncta-defined-area*

<https://downloads.openmicroscopy.org/bio-formats/5.0.3/>

dlmcell: required for all scripts

http://uk.mathworks.com/matlabcentral/fileexchange/25387-write-cell-array-to-text-file/content/dlmcell.m
